# Waned mantle upwelling contributes to a heavily deformed northern smooth plains on Mercury

**DOI:** 10.1038/s41467-026-74975-0

**Published:** 2026-08-26

**Authors:** Jingchun Xie, Yan Zhan, Shengxia Gong, Chengli Huang, Jian Zhang

**Affiliations:** 1 Department of Earth and Environmental Sciences, The Chinese University of Hong Kong, Hong Kong SAR, China; 2 Shanghai Astronomical Observatory, Chinese Academy of Sciences, Shanghai, China; 3 School of Astronomy and Space Science, University of Chinese Academy of Sciences, Beijing, China; 4 NWU-HKU Joint Centre of Earth and Planetary Sciences, Department of Earth and Planetary Sciences, The University of Hong Kong, Hong Kong SAR, China

**Keywords:** Geodynamics, Tectonics

## Abstract

Recent advancements in mapping and interpretation have revealed both numerous contractional landforms and evidence that tectonic activity has persisted into geologically recent times in Mercury’s northern smooth plains (NSP). To investigate the mechanisms behind these observations, we conduct a comprehensive assessment of contractional strain across the NSP. Our analysis reveals that (1) the NSP exhibits higher strain than other terranes on Mercury, and (2) the surface deformation pattern is spatially correlated with the Northern Rise (NR) at the center of the NSP, suggesting an additional driving force. We propose that this elevated strain and its distribution result from waned mantle upwelling induced by deep mantle flow beneath the NR. Sustained variations in dynamic topography likely fueled the long-standing tectonism in the NSP. These findings provide a complementary perspective on Mercury’s evolutionary history and may help illuminate similar processes on other terrestrial planets.

## Abstract (web summary)

Mercury’s vast northern plains are way more cracked and wrinkled than expected! Scientists say a hidden plume of rising mantle pushed up the surface ∼3.7 billion yrs ago - indicating Mercury's interior stayed active far longer than we thought.

## Introduction

Mercury, the smallest and innermost planet in our solar system, offers a unique window into planetary tectonics and thermal evolution. Among its geological features, the northern smooth plains (NSP), formally named Borealis Planitia, stand out as a vast volcanic expanse covering approximately 7% of Mercury’s surface^1–3^. Dated to about 3.7 Ga^4^, the NSP preserves well-defined contractional landforms, such as lobate scarps and wrinkle ridges^5,6^, making it a key area for understanding the tectonic forces shaping Mercury’s surface over time.

**Fig. 1:**
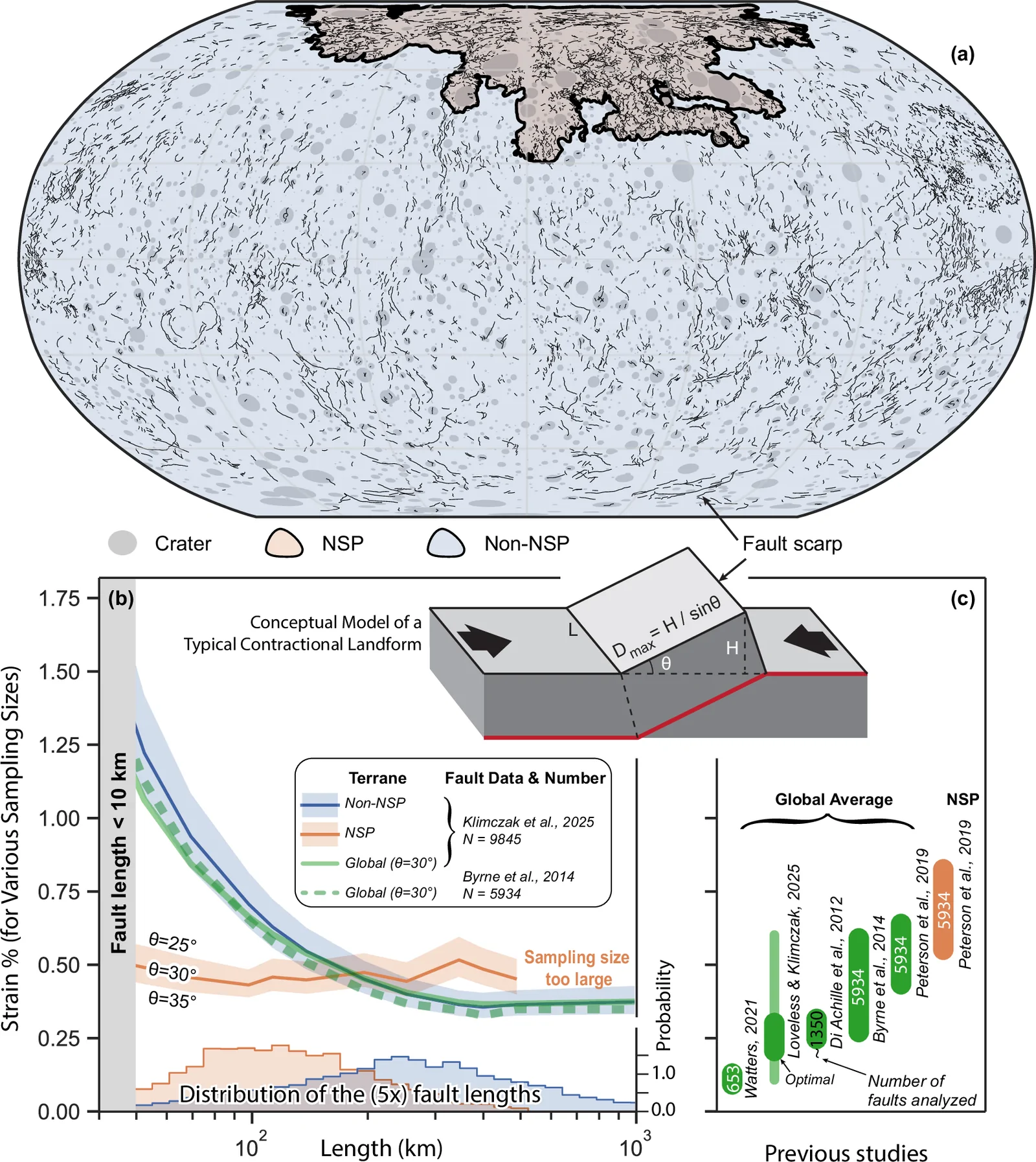
Global mapping of contractional landforms and convergence-tested strain estimation with cross-study comparison for Mercury. **a** Global distribution of contractional landforms and craters across Mercury. **b** Contractional strain for various sampling sizes, which converges toward a stable value representative of the background contraction of each terrane. The distribution of 5 × fault lengths suggests that the largest sampling windows adequately capture the mapped fault population. The inset depicts a conceptual model of a typical contractional landform, where *H*, *θ,* and *L* are the fault relief, dip, and length, respectively, and *D*_*max*_ is the maximum displacement. **c** Comparison of Mercury’s contractional strains from previous studies. Contractional structures are identified from a recently published global tectonic map of Mercury^23^, and the crater database is after^5,62^. Loveless and Klimczak^13^ calculated global contraction using three different datasets^8,12,18^, each with different sample sizes of contractional structures (*n* ≈ 6000, 600, and 100, respectively).

Contractional landforms, most notably thrust fault-related scarps, dominate the tectonic landscape of the NSP (Fig. 1a) and have also been considered to be the product of Mercury’s global contraction^5,7^. However, the NSP preserves a disproportionately large number of contractional landforms^7,8^, contradicting the conventional paradigm that much of global contraction had already ceased prior to the formation of the NSP^5,9^. There may be two possibilities: (1) the contractional strain of the NSP has been overestimated because the common method calculates strain using measurements (e.g., displacement-length ratios) derived from limited populations of representative structures^7,10–12^, with the outcomes sensitive to the measurements and fault number. This concern is supported by a recent fault-number-independent analysis that yields lower estimates of Mercury’s global contraction^13^ than earlier assessments^7,8^, or (2) mechanisms other than global contraction have also contributed to the contractional deformation in this region. If the former is true, it remains difficult to explain the persistence of contractional activity into recent geological times, as evidenced by the identified young grabens (~300 Myr)^14^ and small-scale scarps (~50 Myr)^15^ in the NSP. If the latter stands, one must then ask, what might these mechanisms be?

Previous studies have proposed volcanic loading as another primary driving mechanism^7,12^. However, analysis of fault geometries indicates that the faulting depths of most contractional structures in the NSP exceed expectations for loading-dominated deformation and far surpass the thickness of the volcanic infill that makes up the NSP^3,16–18^. This implies that loading alone could not have provided sufficient horizontal compressive stress to initiate the observed faulting. Furthermore, the pronounced spatial clustering of contractional landforms in the NSP^5,19^ is inconsistent with the expected uniform deformation from volcanic loading, as is the absence of significant spatial variations in lava flow thickness^16^ that would be required to localize loading and associated tectonic deformation^20^. Most importantly, volcanic loading would have been most feasible during Mercury’s hotter, ancient times because the progressively cooled and stiffened lithosphere makes it difficult to support recent geological activity^14,21^. Nevertheless, while we acknowledge that volcanic loading may have contributed to the deformation in the NSP, the available evidence does not support it as the primary driver of the regional contractional structures. Consequently, previously unrecognized mechanisms must be considered, which underscores the substantial gap that remains in our understanding of the evolution of the NSP and of Mercury.

In this research, we comprehensively evaluate contractional strains across Mercury and find that the NSP has experienced stronger contractional deformation than other terranes. We propose that the waned mantle upwelling beneath the Northern Rise (NR) has contributed to the elevated contractional strain and its spatial distribution in the NSP. This process likely provided a sustained force driving surface deformation in this region. Our findings introduce an additional mechanism driving the prolonged tectonism on Mercury and explore the role of regional mantle processes in shaping planetary surfaces. These results provide a complementary perspective on Mercury’s evolutionary history and may help in understanding similar processes on other terrestrial planets.

## Results and discussion

### Greater contractional strain in Mercury’s northern smooth plains

To test whether the NSP experienced greater shortening than the rest of Mercury, we quantify the global contractional strain from mapped thrust fault-related landforms (Fig. 1a). Existing estimates of planetary contraction relate horizontal shortening to fault dip (*θ*), relief (*H*), and length (*L*)^8,10,12^ (Fig. 1b). Building on this framework, we develop a relief-extraction algorithm and combine it with a spatial sampling scheme that evaluates strain over a wide range of length scales (see “Methods” and Supplementary Fig. 1). We spatially aggregate standard fault-based shortening estimates within equal-area sampling circles to compare regional contraction patterns, rather than to define a new physical measure of strain.

To estimate contractional strain, we first extract fault relief from topographic profiles. Second, we convert those measurements into contractional strain estimates within equal-area sampling circles across Mercury using a Fibonacci-sphere framework, which minimizes latitude-dependent area distortion^22^. Third, we examine how strain varies with sampling size to distinguish local structural variability from the large-scale shortening signal. Contractional landforms are taken from a recently published global tectonic map^23^. Faults shorter than 10 km are excluded because their relief is poorly constrained and the strain they represent has a minor influence on final results^7^. Regions without mapped contractional landforms are also excluded, as the absence of structures may reflect either low shortening or resurfacing by large impacts.

We compute strain over a broad range of sampling sizes using assumed fault dips of 25°–35° ^8,12^. The strain estimates are approximately log-normally distributed (Supplementary Fig. 2), indicating that our sampling framework captures the main variation in the data. Assuming global contraction is the primary driver of Mercury’s contractional deformation^7,8,24^ and ignoring factors, such as crustal heterogeneity^25^, the associated large-scale compressive stress field should be approximately uniform across the planet. We therefore use the median strain to characterize the representative background contractional deformation. At small sampling sizes, the estimates are highly variable, especially outside the NSP, where structures are more heterogeneous in scale and distribution (Fig. 1b and Supplementary Fig. 3). Variability within the NSP is lower. As sampling windows increase, median strain for both the NSP and non-NSP terrane decreases and approaches a stable value (Fig. 1b). We interpret these stable, large-scale values as representative of background regional shortening because large sampling windows integrate many structures over broad areas and are less sensitive to local clustering.

Using this framework, strain estimates for the NSP consistently converge to a higher contractional strain than the non-NSP terrane (Fig. 1b). The contrast is clearest at large sampling sizes, where the influence of individual structures is minimized. The distribution of 5 × fault length further suggests that the largest windows adequately capture the mapped fault population, supporting the interpretation that the stable median values reflect integrated background deformation rather than incomplete sampling.

To test robustness, we apply the same sampling framework to previously published global fault datasets^8,12,13^ (Supplementary Fig. 3). Despite a difference of nearly 4000 mapped faults between the dataset utilized here and that of Byrne et al.^8^. The resulting global strain estimates are similar and fall within the range of earlier research^7,8,13^ (Fig. 1c and Supplementary Fig. 4). When evaluated within our Fibonacci-sampling framework, the datasets of Watters^12^ and Loveless and Klimczak^13^ do not show stable convergence, likely because our method is designed for densely mapped fault populations. In these cases, once the sampling size exceeds the lengths of the vast majority of faults (e.g., 85th percentiles of fault length) (Supplementary Fig. 4), the strain continuously decreases toward zero, as the sampling area grows much faster than the cumulative weighted fault length per unit area. This pattern is clearly captured by the relationship between strain estimates and sampling coverage, defined as the fraction of sampling areas that contain at least one fault. Convergence, therefore, depends not only on fault number but also on whether the mapped structures sample Mercury densely enough to resolve long-wavelength shortening. For the datasets with limited faults, an uneven sampling scheme is preferable; we therefore present the estimates and argue that they are consistent to first order (Supplementary Fig. 5). Overall, these comparisons support the robustness of our approach while clarifying the conditions required for convergence and representativeness.

Absolute shortening remains uncertain because key parameters (e.g., fault dip) and the partitioning between folding and fault slip at depth are not adequately constrained for individual landforms. Forward modeling indicates overlapping folding-accommodated shortening across lobate scarps and wrinkle ridges, and larger cumulative strain in lobate scarps likely reflects size or maturity rather than distinct partitioning^18^. Given these non-unique parameterizations, a fault-by-fault revision remains beyond our scope. We therefore retain a standard fault-based framework as a first-order estimator and focus on relative differences between terranes. Under these assumptions, the conclusion is robust: the NSP experienced stronger contractional deformation than the non-NSP terrane.

This finding is unexpected. The NSP formed at approximately 3.7 Ga^4^, after much of Mercury’s global contraction had likely already occurred^9,26^, and its thick volcanic infill may have partly decoupled the surface from deeper shortening^5^. If global contraction were the only driver, the NSP should record less shortening than older terranes. Instead, it records more, implying an additional regional contribution.

### Spatially variable contraction within the northern smooth plains

**Fig. 2:**
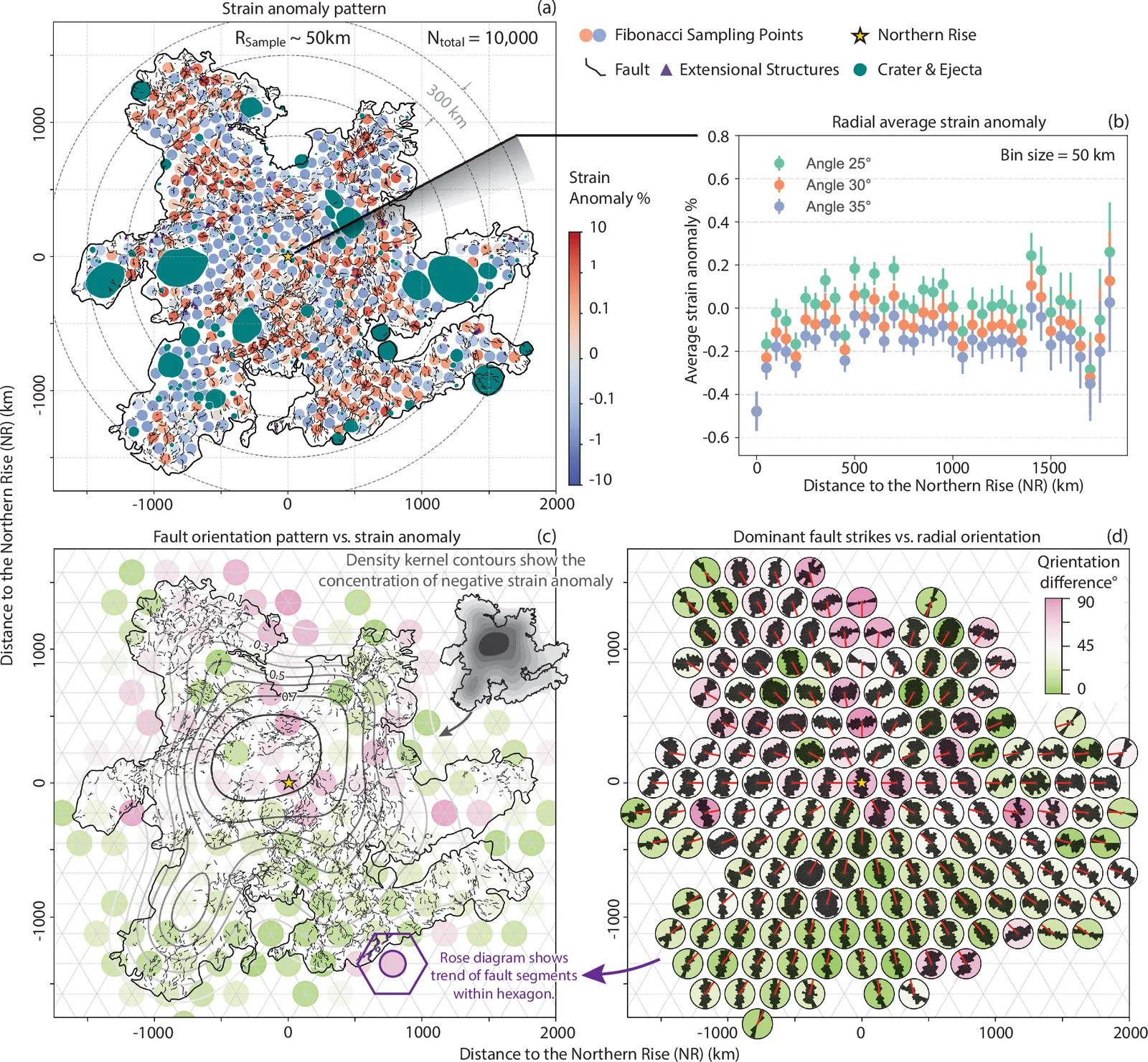
Strain anomalies and fault patterns centered on the Northern Rise. **a** Strain anomaly map of the NSP for Fibonacci sampling points of 10,000. **b** Corresponding radial average strain anomalies at different fault dip angles with a bin size of 50 km. **c** Strain anomaly versus fault orientation pattern, and **d** dominant fault strikes relative to the radial direction from the center of the Northern Rise. A positive strain anomaly indicates stronger-than-average contraction. Maps are reprojected into a local coordinate reference system centered on the Northern Rise. Extensional structures are indicated by equilateral triangles^14^; crater and ejecta distributions follow^5,62^.

Using the same fault-based and equal-area sampling framework, we investigate how shortening is distributed within the NSP via the strain anomaly, defined as the strain at each sampling point minus the NSP average contractional level (~0.5%; Fig. 1). Accordingly, positive anomalies indicate stronger-than-average contraction, whereas negative anomalies indicate weaker-than-average contraction. If the plains simply recorded a uniform response to global contraction, strain should vary only modestly across the province. Instead, the mapped landforms reveal a strongly heterogeneous pattern (Fig. 2).

Contractional strain is comparatively low in the central NSP (i.e., radius ≤300 km), coincident with the NR, and increases outward into the surrounding plains, where contractional structures are denser (Fig. 2a). This produces a broad annular pattern of reduced central shortening and enhanced peripheral contraction, which is further confirmed by the density kernel contours showing a concentration of negative strain anomalies over the NR (Fig. 2c).

The structural distribution supports the same interpretation. Contractional landforms are less developed across the crest of the NR, whereas the adjacent plains contain a denser population of shortening structures. The concentration of stronger contraction around, rather than directly over, the NR is difficult to reconcile with global contraction alone.

**Fig. 3:**
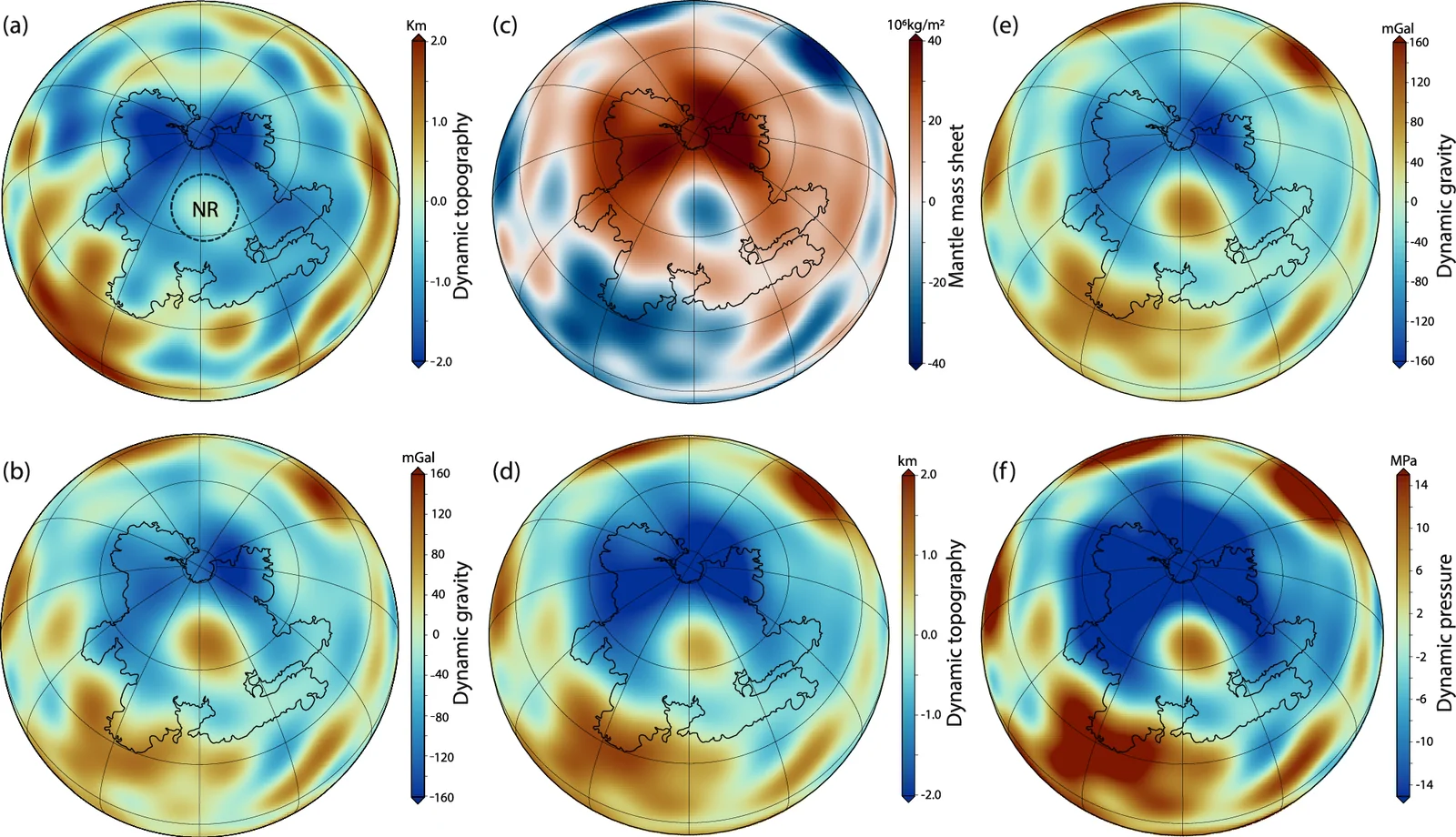
Geophysical evidence for deep support beneath the Northern Rise. **a** Topography and **b** free-air gravity anomalies at spherical harmonic degrees of 15. Results from the mantle dynamic loading model in orthographic projection centered on the Northern Rise (NR) (30^∘^E, 70^∘^N), showing **c** mantle mass sheet, **d** predicted dynamic topography, **e** predicted dynamic gravity, and **f** predicted dynamic pressure. The black solid and dashed lines, respectively, frame the boundary of the northern smooth plains and the NR.

Additional support comes from extensional or non-contractional structures near the rise. Although contraction dominates the NSP overall, these structures indicate that the local stress regime near the NR differed from that of the surrounding plains. Their spatial association with reduced contraction (Fig. 2c) is consistent with uplift above a long-wavelength positive anomaly (Fig. 3), superposed on Mercury’s background contraction.

We further visualize the dominant thrust-fault strikes and their angular differences from the radial trend centered on the NR (Fig. 2c, d). Proximal to the NR, strikes are roughly perpendicular to the radial direction. With distance, strikes tend to align subparallel to the radial direction, except in heavily impact-modified areas and north of the NR, which likely records a distinct tectonic overprint unrelated to NSP formation. We interpret this pattern as consistent with deformation expected from an approximately concentric horizontal compressive stress field around the NR, although the observations directly constrain contractional strain and structural orientations rather than the stress itself.

These spatial patterns are therefore as important as the elevated strain magnitude. The excess shortening in the NSP is not uniformly distributed, but organized around the NR, suggesting a regional source centered on the rise.

### Geophysical evidence for deep support beneath the Northern Rise

The strain pattern implies that the NR is linked to a deeper process rather than being a purely surface feature. We therefore examine its topographic and gravity characteristics using the latest gravity and topography data^27,28^.

The NR is a broad, long-wavelength (e.g., spherical-harmonic degrees ≤15) topographic high superposed on the NSP (Fig. 3a). Its scale and smooth morphology distinguish it from shorter-wavelength volcanic or tectonic edifices and instead suggest support at depth^29^. Gravity data likewise shows positive anomalies of similar scale (Fig. 3b). This coincidence of broad uplift and low-wavelength gravity support is hard to explain by shallow loading alone^27,28^, particularly in the absence of a sufficiently localized surface construct. Instead, a deep-seated buoyancy more naturally generates regionally coherent uplift and gravity anomalies^30^, consistent with the NR.

This interpretation is also consistent with prior evidence that the northern plains overlie an anomalous mantle domain^1,3^. Geochemical observations indicate that the NSP is compositionally distinct from older surrounding terrains^31^, suggesting unusually extensive or subsequent mantle melting. Other authors have argued for prolonged magnetic activity^32^ and late-state tectonism^14,15^ in the northern hemisphere. Together, these observations are consistent with a long-lived thermochemical anomaly beneath the NSP.

We apply a mantle dynamic loading model (see “Methods” and Supplementary Table 1) to examine how a deep buoyant source reshapes the geological context. For a mass body seated at a depth of 350 km, we estimate its horizontal extent to about 730 km, with a density contrast of 8 × 10^6^ kg/m^2^ (Fig. 3c). This low-density body induces mantle upwelling that deforms the core-mantle and lithosphere-mantle boundaries, resulting in topography (Fig. 3d) and gravity (Fig. 3e) anomalies while generating upward pressure at the surface (Fig. 3f). An alternative model, which assumes topography is fully supported by mantle dynamics^33^, produces nearly identical results (Supplementary Fig. 6) and aligns with prior research^34^.

A deep buoyant source provides a plausible explanation for the observed tectonic pattern in the NSP. Uplift above the smooth plains would reduce horizontal contraction, or even generate local extension, while enhancing shortening in the surrounding plains as the lithosphere adjusted to the positive anomaly. The observations, therefore, point to a mantle-supported rise that perturbed Mercury’s background compressive stress field.

Geophysical data alone, however, cannot uniquely determine the longevity, amplitude, or exact nature of the anomaly. They do not distinguish whether the buoyancy is thermal, compositional, or thermochemical, nor do they show how it interacts with Mercury’s cooling lithosphere through time. We therefore turn to numerical models.

### Mantle upwelling reproduces the uplift and contraction pattern of the northern smooth plains

To investigate whether a buoyant anomaly beneath the NR can explain the observations, we perform thermo-mechanical simulations of mantle upwelling beneath Mercury’s northern lithosphere. Our goal is not to reproduce every mapped structure, but to test whether a plausible upwelling can generate the first-order pattern: broad uplift, reduced contraction or local extension above the topographic rise, and enhanced shortening in the surrounding NSP.

Each simulation spans 3.7 Ga, assuming that the buoyant mantle anomaly formed coevally with the NSP^4,35^ and that the lithosphere behaved viscously at that time. The simulations, therefore, aim to reproduce the first-order large-scale deformation field rather than individual brittle structures. We explore a range of parameters describing anomaly strength, geometry, duration, and lithospheric properties (see “Methods” and Supplementary Table 2).

**Fig. 4:**
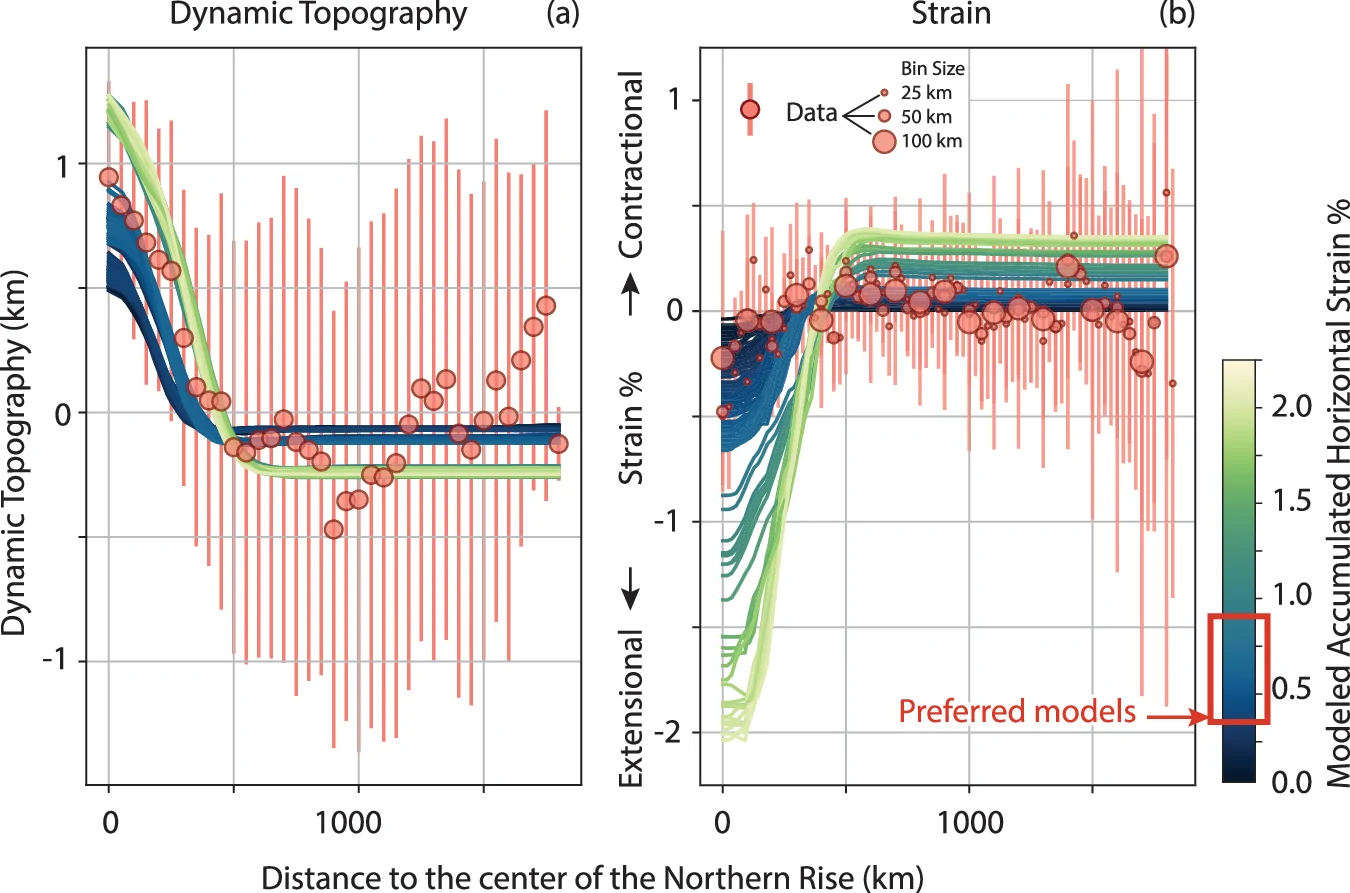
Results from two-dimensional axisymmetric numerical simulations. **a** Final dynamic topography compared with the mean observed elevation of the NSP (vertically offset by 500 m to set far-field topography to zero). **b** Modeled accumulated horizontal strain compared with the mean strain anomaly of the NSP. All quantities are averaged radially about the Northern Rise (NR). Red lines mark ±1 standard deviation.

In all simulations (Supplementary Fig. 7), the upwelling consistently rises toward the base of the lithosphere. Confronted with the lithosphere’s mechanical resistance, it then spreads laterally along this boundary. Over time, this process molds the anomaly into a thin, laterally extensive sheet, consistent with our geophysical inversions. Early in the upwelling, the lithosphere arches upward to form a long-wavelength rise, followed by gradual subsidence after several hundred million years (Fig. 4 and Supplementary Fig. 8). Horizontal shortening above the center is reduced, while contraction is concentrated around the flanks (Fig. 4). This closely matches the observed strain distribution pattern in the NSP.

Mantle upwelling likely generated the melts that formed the NSP lavas, but the relative timing of contractional deformation and lava emplacement remains uncertain. Because the mapped contractional structures likely record cumulative deformation and may include reactivated pre-existing structures^18^, the observed strain field is interpreted as a time-integrated measure of regional shortening rather than the product of a single instantaneous stress. We therefore integrate the horizontal strain under the assumption that most deformation postdates lava emplacement, marked by the onset of the topographic rise decline. Across all simulations, a sufficiently broad and persistent upwelling produces a consistent deformation pattern (Fig. 4a, b).

In models with stronger or longer-lived buoyancy, tensile or low-compressive conditions develop above the rise, allowing limited extension or suppression of contraction in the uplifted region (Supplementary Fig. 7). At the same time, contraction intensifies around the margins, where flexural adjustment and lateral stress transfer focus contractional strain. The upwelling therefore redistributes, rather than eliminates, the effects of global contraction.

A key finding is that this tectonic signature can outlast active upwelling. After buoyant support wanes, the uplifted lithosphere relaxes and subsides, but the inherited topography and stress gradients continue to influence deformation for an extended period (Supplementary Fig. 8). Preferred models are those that reproduce both the wavelength and amplitude of the NR topography together with the observed radial pattern of reduced central contraction and enhanced peripheral contraction. In the preferred model, elevated shortening in the surrounding plains persists for billions of years, consistent with continued deformation after emplacement of the NSP^14,15^.

This behavior helps explain why the relatively young NSP records unexpectedly high contractional strain. If mantle upwelling began during or shortly before plains emplacement, it could have established a long-lived tectonic pattern that persisted into later stages of Mercury’s cooling. The present fault distribution would then record both the direct effects of uplift and the subsequent adjustment of the lithosphere.

Model success depends strongly on anomaly scale and lithospheric strength. Narrow or short-lived anomalies produce deformation that is too localized and fail to reproduce the wavelength of the NR or the regional extent of shortening. Broad, sustained anomalies perform much better. The preferred models also require a lithosphere capable of transmitting stresses over regional distances. Together, these results support a mechanically coherent lithosphere above a mantle source of regional extent.

Although the simulations reproduce the observed topography and strain pattern (Fig. 4), they do not, by themselves, explain the radial fault orientations around the NR (Fig. 2d). We therefore perform an additional three-dimensional elastic analysis to determine the stress field induced by NR loading (see “Methods” and Supplementary Fig. 9). Outside the NR, the maximum horizontal compressive stress is concentric about the load center, implying radial contractional faults because reverse-fault strike is expected to be perpendicular to the maximum compression. This stress pattern is controlled primarily by the geometry of the NR load and is only weakly sensitive to the position of the imposed outer constraint, indicating that the predicted fault orientation is a robust first-order consequence of NR-centered loading.

### Controls on the upwelling-induced surface deformation

The surface deformation induced by mantle upwelling depends on both the properties of the buoyant mantle anomaly and Mercury’s interior structure, including parameters such as lithospheric thickness, viscosity, and thermal state.

**Fig. 5:**
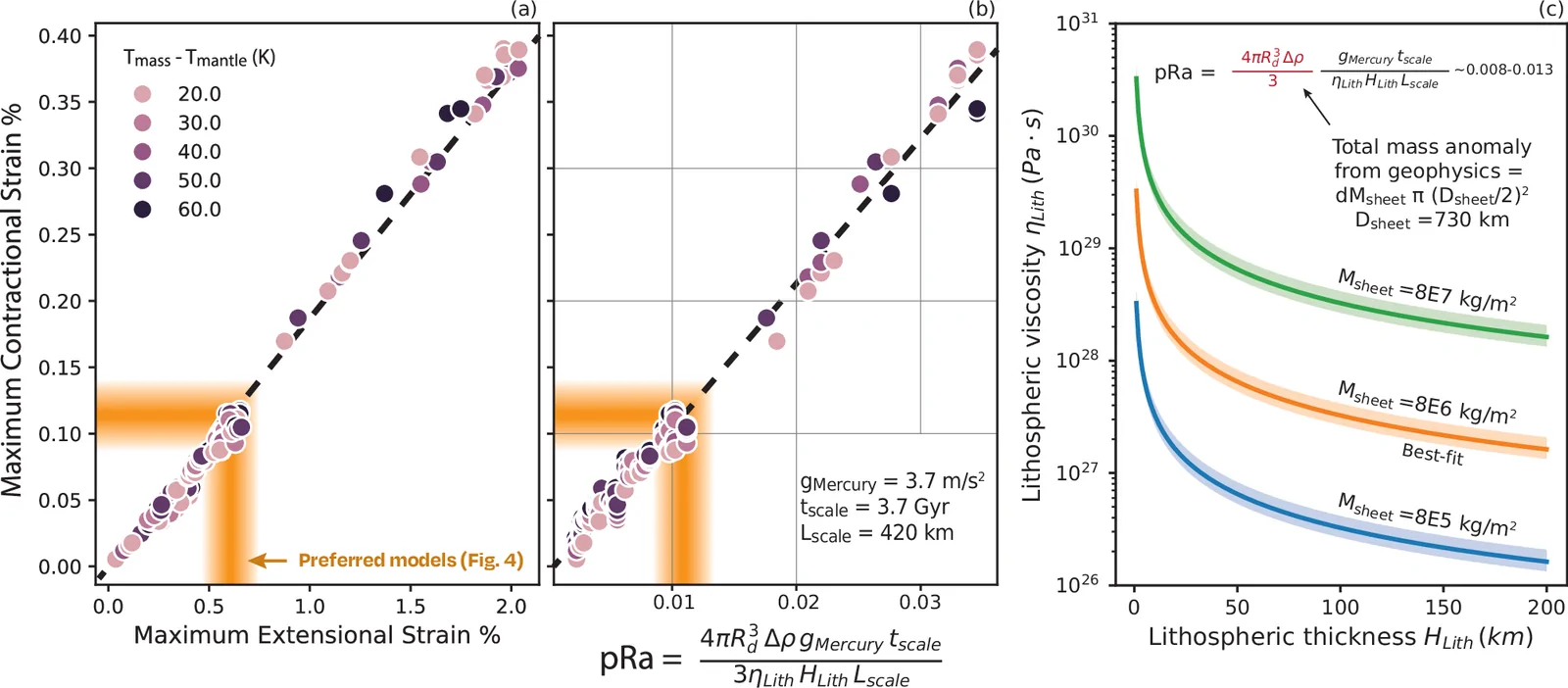
Strain scaling and lithospheric constraints via the pseudo-Rayleigh number. Comparative relationships between **a** the maximum contractional strain and the maximum extensional strain. **b** The maximum contractional strain and nondimensionalized maximum extensional strain by pseudo-Rayleigh number (*pRa*). The *pRa* is defined as $pRa=\frac{4\pi {R}_{d}^{3}{{\Delta }}\rho {g}_{Mercury}{t}_{scale}}{3{\eta }_{lith}{H}_{lith}{L}_{scale}}$, where *R*_*d*_ is the radius of initial mass anomaly, *η*_*lith*_ and *H*_*lith*_ are respectively the lithospheric viscosity and thickness. *g*_*Mercury*_ is the surface gravitational acceleration of Mercury, *t*_*scale*_ is the time scale of the simulation, and *L*_*scale*_ is the thickness of Mercury’s silicate shell^38^. **c** The lithospheric viscosity and thickness derived from the surface strain and geophysical inversion through *pRa*.

Surface uplift is controlled mainly by buoyancy strength. Larger density contrasts, stronger thermal anomalies, or more vigorous upwelling generate greater uplift and stronger stress reorganization (Fig. 5a). As uplift increases, contraction above the NR weakens and may transition to extension, while contraction intensifies around the flanks. By contrast, weaker anomalies produce only modest topography and little stress redistribution.

The wavelength of deformation depends chiefly on the lateral extent of the anomaly and the flexural stiffness of the lithosphere. Broad anomalies generate long-wavelength uplift and distributed contraction comparable to that observed in the NSP (Fig. 5b), whereas narrow anomalies localize deformation too strongly. Lithospheric thickness exerts a similar control: a stronger lithosphere transmits stresses over greater distances, while a weaker one concentrates stress close to the upwelling center.

Longevity reflects the combined timescales of buoyancy decay and lithospheric relaxation. Rapid anomaly dissipation diminishes uplift and stress anomalies, limiting long-term shortening, while gradual decay or initially large uplift leaves a long-lived topographic and mechanical imprint that persists after active ascent ceases.

These trade-offs define the class of successful models. They require an anomaly broad enough to match the wavelength of the NR, strong enough to reduce contraction above the rise and enhance it in the surrounding plains, and persistent enough to leave a long-lived tectonic imprint. The lithosphere must also be strong enough to transmit these stresses regionally, but not so rigid that deformation is suppressed. The preferred solutions, therefore, involve a broad, long-lived mantle anomaly beneath a mechanically coherent lithosphere.

This parameter dependence also implies the nature of this dynamic system, namely, the interplay between the buoyancy force and lithospheric resistance. To characterize this interaction, we introduce a dimensionless parameter, the pseudo-Rayleigh number (*pRa*; Fig. 5), which quantifies the ratio of the buoyant force from the mantle anomaly to the lithospheric resistance. The modeled maximum contractional and extensional strains scale proportionally with *pRa* (Fig. 5), demonstrating that surface deformation reflects the dynamic balance between these two forces.

To reproduce the observed topography and strain distribution across the NSP, the maximum extensional strain at the NR is estimated to be ~0.5–0.7%, corresponding to a *pRa* of approximately 0.01. This constrained *pRa* establishes a critical connection between the geophysical inversion results (Fig. 3) and the surface deformation recorded in geological structures, in a manner consistent with conservation of the mantle mass anomaly. Combining the inversion-derived areal density of the mass sheet with surface strain estimates, *pRa* can further constrain Mercury’s lithospheric properties.

Given uncertainties in the areal density estimate, we explore several orders of magnitude to infer the corresponding lithospheric thicknesses and viscosities (Fig. 5c). For example, for an areal density of 8 × 10^6^ kg/m^2^ and *pRa* ≈ 0.01, a lithospheric thickness of 50–200 km implies a viscosity on the order of 10^27^–10^28^ Pa s. These results highlight the potential of combining topography, gravity, and surface deformation data to jointly infer the rheological and thermal structure of Mercury’s lithosphere.

### Tectonic evolution of the NSP and its implications to Mercury’s interior

Our results show that deformation in the NSP is unlikely to be explained by Mercury’s global contraction alone. The NSP records greater contractional strain than surrounding terranes, even though it is younger and formed after much of Mercury’s secular cooling had already occurred. It also shows a distinct spatial distribution pattern: contraction is weaker over the NR and stronger in the surrounding plains. Together, these observations require a regional process superposed on the background effect of planetary contraction.

**Fig. 6:**
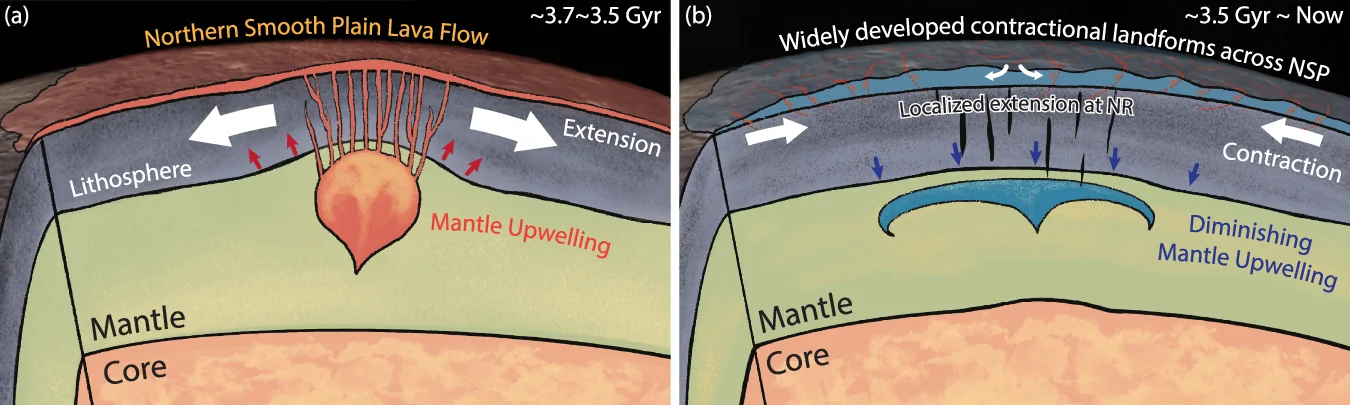
Schematic illustration of mantle-upwelling-driven deformation in the NSP. **a** Extensional deformation and volcanic activity during NSP formation. **b** Transition to contractional deformation following the decline of mantle upwelling.

The broad topography and gravity signature of the NR are most consistent with support by a deep buoyant anomaly, and thermo-mechanical simulations show that upwelling initially generates the uplift and reduced central contraction (Fig. 6a). As Mercury cools and the upwelling wanes, the dynamic topography relaxes and enhances peripheral contraction (Fig. 6b).

The preferred models (Fig. 4) require a broad, long-lived mantle anomaly beneath a mechanically coherent lithosphere. This interpretation implies that the NSP is not simply a passive site of lava emplacement on a cooling planet. Instead, plains emplacement and later deformation are likely linked to the same mantle process, rooted in a global volcanic episode^4^ potentially triggered by large impacts comparable in scale to those that formed Caloris or Rembrandt basins^36,37^. A long-lived buoyant anomaly could have promoted resurfacing while also reorganizing the regional stress field. In that sense, the NSP preserves coupled evidence for both extensive magmatism and anomalous tectonism.

More broadly, the results imply that Mercury’s mantle remains regionally dynamic later in its history. This inference challenges our prior understanding of an inactive mantle because of its relatively thin thickness^38–41^. This does not require vigorous global or large-scale convection after 3.7 Ga, but it does indicate that regional thermochemical anomalies survived long enough to modify late lithospheric evolution. The northern hemisphere may therefore preserve evidence of localized interior activity superposed on long-term secular contraction.

These results also affect how fault-based shortening is interpreted on Mercury. Such estimates are often treated as records of global contraction alone, but the NSP shows that regional mantle processes can modify both the magnitude and spatial distribution pattern. Heterogeneity in Mercury’s tectonic record may thus reflect localized interior dynamics as well as resurfacing, preservation bias, or lithologic differences.

Uncertainties remain. Our deformation estimates depend on assumptions about fault dip, structural geometry, and strain partitioning among contractional landforms. The models and geophysical inversions strongly favor deep support beneath the NR but do not uniquely resolve whether the buoyancy is thermal, compositional, or both. These uncertainties affect the details of the mechanism. Future work should incorporate more comprehensive treatments containing lithospheric yielding^42–44^ and melt migration^45^, combined with geochemical constraints and advanced simulation techniques, to better assess the coupling between surface tectonism and mantle dynamics. Nevertheless, our main conclusion remains robust: the NSP preserves a regional tectonic overprint inconsistent with global contraction alone.

Taken together, our study supports a model in which the NSP records the interaction of regional mantle-driven deformation with Mercury’s long-term contraction. The NR is best interpreted as the surface expression of a broad, long-lived buoyant anomaly that reorganized the regional stress field and amplified contraction in the surrounding plains. Mercury’s late tectonic evolution is therefore shaped not only by planetary cooling but also by localized mantle dynamics. This coupled mechanism may also operate on other rocky planets, particularly those that likely preserve active or residual mantle convection, such as Mars^46^ and Venus^47–49^.

## Methods

### Expression of contractional strain

The lithospheric mechanical behavior can be classified into two idealized deformation modes: the upper lithosphere undergoes pressure-dependent brittle deformation, whereas the lower lithosphere is governed primarily by strain rate- and temperature-dependent ductile deformation^42^. This classification arises from the progressive increase in ambient temperature and confining pressure with depth, which significantly influences lithospheric rheology. The transition between these two distinct deformation regimes occurs at a critical depth known as the brittle-ductile transition (BDT) zone, where the dominant deformation mechanism shifts from brittle fracturing to plastic flow^42,50^. This zone marks the maximum faulting depth, *T*, at which brittle failure and seismic rupture can occur. Beyond this depth, further fault growth is primarily along strike, while down-dip height (*h*_*h*_) remains constant (i.e., *h*_*h*_ ≈ *T*). This expression is valid if dip corrections are neglected for simplicity^51–53^. These through-going faults, here defined as large-scale faults, accommodate a disproportionately large fraction of total strain and seismic moment, featuring *L* > *h*_*h*_ ≈ *T*.

The contractional strain (*ϵ*) accommodated by large-scale structures can be estimated by ref. ^12^:

1 $$\epsilon=\frac{cos(\theta ){\sum }_{i}^{n}DL}{A}$$

Where *θ* is the fault’s dip angle, *A* is the area of the faulted region. *D* and *L* are the fault’s maximum displacement and length, respectively. Usually, we can define a scaling factor (*γ*) to be related to *D* and *L* with the following form:

2 $$\gamma=\frac{D}{L}=\frac{H}{Lsin(\theta )}$$

Where *H* is the fault’s maximum relief. Substituting Eq. 2 into Eq. 1, we can yield:

3 $$\epsilon=\frac{\gamma cos(\theta )\mathop{\sum }_{i}^{n}{L}^{2}}{A}$$

Since large-scale tectonic structures are known to accommodate substantial strain, Eq. 3 provides a rough estimation of areal strain in faulted regions or across the entire planet. However, recent advancements in the interpretation of Mercury’s remote sensing data have revealed numerous small-scale shortening landforms^5,19^, highlighting the need to incorporate these small structures when comparing areal strain intensities across different terranes.

For small-scale structures, the cumulative contractional strain is derived from the Kostrov’s relation with the following form^52^:

4 $$\epsilon=\frac{1}{AT}\mathop{\sum }_{i}^{n}{h}_{h}LDsin(\theta )cos(\theta )$$

Where *T* is the maximum faulting depth (BDT depth), *h*_*h*_ is fault’s down-dip height. The remaining parameters are defined as the same as above. Additionally, structural analyses suggest that most faults display elliptical geometries, with their aspect ratios (*L*/*h*_*h*_) typically constrained within the range of 2–3 (e.g.,^54^). We use this observation only in the small-scale structures as a device to eliminate the down-dip height (*h*_*h*_); it is not imposed on large, through-going faults for which *h*_*h*_ ≈ *T*.

Assuming the aspect ratio is 2 and substituting Eq. 2 into Eq. 4, we can return the areal contractional strain for small-scale structures through:

5 $$\epsilon=\frac{sin(\theta )}{2T}\frac{\gamma cos(\theta )\mathop{\sum }_{i}^{n}L\cdot {L}^{2}}{A}$$

The estimation of contractional strain accommodated by thrust fault-related landforms requires four key parameters: maximum faulting depth (*T*), dip angle (*θ*), length (*L*), and relief (*H*). Following previous estimates^55,56^, we set the BDT depth to 30 km in Mercury’s lithosphere. This number shows consistency with (1) the average crustal thickness derived from geophysical inversions^29,57^, (2) faulting depths predicted by the lithospheric mechanical model^58^, and (3) also falls within the range of penetration depth estimated from analyses of fault underlying geometries^17,18^. We consider dip angles of 25°–35°, consistent with prior work^8,12^. Consequently, the precise determination of reliefs becomes critical for accurate strain calculation.

### Extraction of the thrust fault’s relief

The general method for computing fault’s relief involves extracting topographic profiles within a specified distance on both sides of the fault trace and selecting representative profiles that capture the key morphological features of those structures. We develop a refined relief extraction algorithm including the following steps:

1. For each fault, we first establish a local coordinate reference system centered at its midpoint to minimize projection distortions and permit an accurate calculation of its local length *L*_*c*_. Under the assumption of a uniform local stress field oriented orthogonally to the fault’s strike (defined by its end-to-end azimuth), we then generate a series of equally spaced parallel transects, where the length of each transect is defined as 1.5 × min(*L*_*c*_, 50) km, ensuring systematic and representative sampling of deformation features on both sides of the fault structure.
2. To minimize the biasing effects of impact craters on our measurements, we build a comprehensive classification scheme for fault-crater spatial relationships, categorizing them into five distinct topological types: (1) within, (2) crosses, (3) touches, (4) intersects, and (5) disjoint. Using this scheme, we systematically filter the sampling points along each transect depending on their spatial relationship to nearby craters. For example, in cases where the fault intersects a crater rim (i.e., relationship type 4), we keep only those sampling points located within the crater rim. To maintain statistical robustness while implementing this filtering approach, we apply a conservative threshold that balanced data quality with sample size preservation. For transects requiring data interpolation after filtering, we apply the inverse distance weighting method (IDW) to reconstruct complete topographic profiles, thus ensuring consistent transect lengths across all measurements while preserving the original topographic signal.
3. Following the above preprocessing steps, each transect profile is treated as an input signal. We concatenate all profiles into a composite signal for batch processing and then employ a bandpass filter to isolate waveforms within a specific frequency range. For alignment, we implement cross-correlation analysis using the first profile as the reference waveform. The aligned profiles undergo ensemble averaging to derive the characteristic waveform, with uncertainty bounds quantified as ±2*σ* (two standard deviations).
4. For each characteristic waveform of distinct frequency bands, we quantify the maximum topography difference near its midpoint as the relief. The derived relief values are then subjected to statistical analysis.

The flow chart of this process is shown in Supplementary Fig. 1.

### Calculation of areal strain

The conventional approach uses Eq. 3 to estimate strains accommodated by large-scale structures from fundamental fault morphological parameters. While this approach provides a first-order estimate of Mercury’s global or regional contractional strain, it lacks the spatial resolution needed to characterize strain distribution patterns. To address this limitation, we reformulate Eqs. 3 and 5 into a line-density-like form:

6 $$\epsilon=\frac{\mathop{\sum }_{i}^{n}{w}_{i}{L}_{i}}{A}$$

where *w* is the weight assigned to each fault according to its scale. For small structure, the expression becomes: $w=\frac{\gamma sin(\theta )cos(\theta ){L}^{2}}{2T}$, while for large-scale fault, *w* = *γcos*(*θ*)*L*. A large search area (*A*) reflects the overall deformation, whereas a small one captures the local deformation.

We model Mercury as a Fibonacci sphere to divide the surface into multiple search areas of equal area, which is inversely proportional to the number of Fibonacci points distributed on the sphere. Accordingly, the larger the points, the smaller the search area. In this case, the distance (*d*) between any two adjacent points on the sphere is approximated as:

7 $${d}_{i,i+1}=2{R}_{m}\sqrt{\frac{\pi }{N\sqrt{3}}}$$

where *R*_*m*_ is the mean radius of Mercury, *N* is the number of Fibonacci points. Here, we define the radius of the sub-region as a circular area with a radius of 1.5 *d*_*i*,*i *+ 1_, also referred to as the search radius.

For each circular area (*C*), we only consider the fault lines that intersect the circle. Therefore, the corresponding areal strain is:

8 $$\forall {\epsilon }_{c}=\frac{\mathop{\sum }_{i}^{n}{w}_{i}{L}_{i}\cap C}{{A}_{c}}$$

where the parameters are defined above.

The areal-strain values reported here do not represent a new mechanical definition of strain. Rather, they are obtained by spatially reformulating conventional fault-based shortening estimates: strain contributions inferred from mapped fault relief are integrated within equal-area sampling circles so that regional patterns can be compared consistently across the surface.

### Mantle dynamic loading model

We employ the framework developed by ref. ^59^ to analytically study dynamic flows within a rocky planet. In this methodology, the mantle is considered to be an incompressible Newtonian fluid, and the flow is driven by a density anomaly in the mantle with certain viscosity structures. Following their approach, the viscous flow at radius *r* in a sphere is expressed as a set of first-order differential equations:

9 $$\frac{{{{\rm{d}}}}{{{\bf{u}}}}}{{{{\rm{d}}}}\nu }={{{\bf{A}}}}{{{\bf{u}}}}+{{{\bf{a}}}}$$

Here $\nu=\ln (r/R)$ is a defined variable, and **u** is a vector that contains four components expressed as:

10 $${{{{\bf{u}}}}}_{lm}(r)=\left[\begin{array}{c}{v}_{lm}^{r}(r)\\ {v}_{lm}^{\theta }(r)\\ \left[{\tau }_{lm}^{rr}(r)+\rho (r)g(r){N}_{lm}(r)\right]\frac{r}{{\mu }_{0}}\\ {\tau }_{lm}^{r\theta }(r)\frac{r}{{\mu }_{0}}\end{array}\right]$$

in which, the first two components, ${v}_{lm}^{r}$ and ${v}_{lm}^{\theta }$, are the spherical harmonic coefficients at degree *l* and order *m* of radial and poloidal velocities, ${\tau }_{lm}^{rr}$ and ${\tau }_{lm}^{r\theta }$ are those of normal and shear stresses, and *N*_*lm*_ is the local equipotential surface, respectively. **A** is a dimensionless matrix that correlates to the mantle viscosity, and **a** is a vector that is associated with the mantle density anomalies.

We hypothesize that the mass anomalies are constrained within a thin mass sheet located at a given depth *R*_*Φ*_. The matrix **A** and vector **a** are:

11 $${{{\bf{A}}}}=\left[\begin{array}{cccc}-2&l(l+1)&0&0\\ -1&1&0&\frac{{\mu }_{0}}{\mu }\\ 12\frac{\mu }{{\mu }_{0}}&-6l(l+1)\frac{\mu }{{\mu }_{0}}&1&l(l+1)\\ -6\frac{\mu }{{\mu }_{0}}&[4l(l+1)-2]\frac{\mu }{{\mu }_{0}}&-1&-2\end{array}\right]\quad \quad {{{\rm{and}}}}\quad \quad {{{\bf{a}}}}=\left[\begin{array}{c}0\\ 0\\ g({R}_{\Phi }){\Phi }_{lm}\frac{{R}_{\Phi }}{{\mu }_{0}}\\ 0\end{array}\right]$$

where *μ* and *μ*_0_ are the viscosity and the referenced viscosity, respectively. *Φ*_*lm*_ is the surface density of the thin mass anomaly.

Assuming a constant viscosity structure within the mantle (in this case, *A* would be independent of *r*), a free slip condition at the core-mantle boundary *R*_*C*_ (the radial velocity is zero and there’s no shear stress), and a no-slip boundary condition at the Mercury’s surface *R* (thus the velocities are both zero), Eq. 9 becomes:

12 $$\left[\begin{array}{c}0\\ 0\\ {\tau }_{lm}^{rr}(R)+\rho (R)g(R){N}_{lm}(R)\\ {\tau }_{lm}^{r\theta }(R)\end{array}\right]={e}^{-A\ln \frac{{R}_{C}}{R}}\left[\begin{array}{c}0\\ {v}_{lm}^{\theta }({R}_{C})\\ {{\Delta }}\rho ({R}_{C})g({R}_{C}){C}_{lm}\frac{{R}_{C}}{R}\\ 0\end{array}\right]+{e}^{-A\ln \frac{{R}_{\Phi }}{R}}\left[\begin{array}{c}0\\ 0\\ g({R}_{\Phi }){\Phi }_{lm}\frac{{R}_{\Phi }}{R}\\ 0\end{array}\right]$$

There are six unknowns, that is, ${\tau }_{lm}^{rr}(R)$, *N*_*lm*_(*R*), ${\tau }_{lm}^{r\theta }(R)$, ${v}_{lm}^{\theta }({R}_{C})$, *C*_*lm*_, and *Φ*_*lm*_, in the equations, and *C*_*lm*_ is the shape of the core-mantle boundary. Since ${\tau }_{lm}^{rr}(R)$ is related to the dynamic topography by:

13 $${\tau }_{lm}^{rr}(R)=({H}_{lm}^{dyn}-{N}_{lm}(R))\rho (R)g(R)$$

and

14 $${N}_{lm}(R)=\frac{4\pi GR}{g(R)(2l+1)}\left[\rho (R){H}_{lm}^{dyn}+{\Phi }_{lm}{\left(\frac{{R}_{\Phi }}{R}\right)}^{l+2}\right]$$

Accordingly, the unknowns could be easily solved for a unit surface density (i.e., *Φ*_*lm*_ = 1), from which we get the so-called dynamic kernels.

Following^34^, our model incorporates the following key parameters: a mean crustal thickness of 35 km with a bulk density of 2900 kg/m^3^ (representing a less porous mafic composition), and a mantle-crust density contrast of 250 kg/m^3^. Model configurations are listed in Supplementary Table 1.

Assuming the dynamic flow in our model is driven by a mass sheet anomaly located at 350  km depth, immediately above Mercury’s core-mantle boundary^38^. Within an iso-viscous mantle framework, we construct depth-dependent dynamic loading kernels for unit mass sheet anomalies. The boundary conditions are specified as follows: a free-slip condition at the core-mantle boundary and a non-slip condition at the planetary surface.

Through these configurations, we infer the distribution of mantle mass anomalies by assuming that the observed long-wavelength topography and gravity signals around the NR result from the combined effects of airy isostatic compensation and mantle dynamics. The resulting dynamic topography, gravity, and pressure are then calculated by multiplying the dynamic kernels with the derived mantle mass anomalies (refer to ref. ^29^ and ref. ^33^ for more details).

### Mantle convection numerical simulation

We consider a simplified axisymmetric incompressible mantle convection model under the assumption that the lithosphere behaved viscously at the time of upwelling. The axisymmetric formulation is intended to capture the first-order radial structure of the deformation field and does not reproduce all local azimuthal variations. The basic equations governing the highly viscous fluid motion contain the conservations of mass, momentum, and energy with the following form:

15 $$\left\{\begin{array}{l}\nabla \cdot u=0\hfill\\ -\nabla \cdot [2\eta (\dot{\epsilon }(u)-\frac{1}{3}(\nabla \cdot u))]+\nabla p=\rho g\\ \rho c(\frac{\partial T}{\partial t}+u\cdot \nabla T)-\nabla \cdot k\nabla T=0\end{array}\right.$$

where *u* is the velocity, *η* is the viscosity, and $\dot{\epsilon }(u)$ is the strain rate given by $\frac{1}{2}(\nabla u+\nabla {u}^{tr})$, with *tr* representing transpose. *p* is the pressure, *ρ* is the density, *c* is the specific heat capacity, *k* is the thermal conductivity, and *T* is the temperature.

The viscosity is formulated in a Arrhenius form^37^:

16 $$\eta={\eta }_{0}{e}^{\frac{E+pV}{R}(\frac{1}{T}-\frac{1}{{T}_{ref}})}$$

where *η*_0_ is the reference viscosity, *E* is the activation energy, *p* is the pressure, and *V* is the activation volume. *T*_*ref*_ is the reference temperature. In addition, the density also changes with temperature variations through:

17 $$\rho={\rho }_{0}[1-\alpha (T-{T}_{ref})]$$

where *ρ*_0_ is the reference density, and *α* is the thermal expansion coefficient.

Our study domain is not large enough to consider the planetary curvature; therefore, we use a rectangular domain measuring 3600 × 420 km, consisting of a lithosphere (i.e., crust + stagnant lid) and a convecting mantle. The vertical structure of our geometry is similar to previous research^40,60^. The top boundary is prescribed as a free surface, while the bottom boundary is a non-slip condition. Both lateral boundaries are thermally insulated and are assigned tangential velocities. No basal heating is applied in this model. At the initiation of the simulation, a circular mass anomaly is introduced near the base of the mantle, accompanied by a thermal anomaly of identical geometry.

Other configurations are referenced from prior thermodynamic studies of Mercury and the mantle dynamic loading model listed in Supplementary Table. 2.

### Three-dimensional elastic model

We use COMSOL Multiphysics 6.2 to construct a three-dimensional quasi-static elastic model of Mercury’s lithosphere. The model domain is a sphere of radius of 2440 km, with material properties representative of silicate lithosphere (the parameters do not affect the stress field orientation) (Supplementary Table 3). A localized circular surface load of radius *R*_*NR*_ is imposed at NR as a follower pressure:

18 $$p=\rho {g}_{M}h$$

where *ρ* is the density, *g*_*M*_ is the surface gravitational acceleration of Mercury, and *h* is the average height of the load at the NR.

Because our aim is to examine stress orientation rather than stress magnitude, the exact elastic parameters and load amplitude are not expected to affect the first-order pattern of stress trajectories, whereas the load geometry is expected to be important. We therefore vary the radius of the central load from 200 to 600 km and the distance to the fixed outer constraint from 1000 to 2000 km. Across all models, we analyze the orientation of the maximum horizontal compressive stress.

## Supplementary information

Supplementary Information

Transparent Peer Review File

## Data Availability

The results-related data generated in this study have been deposited in Figshare.

## Code availability

The Python script developed to reproduce the results for this study has been deposited in Zenodo^61^.
