# Supplementary material for: Waned mantle upwelling contributes to a heavily deformed northern smooth plains on Mercury
Source: Nat Commun. 2026 Aug 26;17:9064. doi: 10.1038/s41467-026-74975-0 (PMC13519025; doi:10.1038/s41467-026-74975-0)
Supplement: Supplementary file 1 [file 41467_2026_74975_MOESM1_ESM.pdf]

Supplementary to "Waned mantle upwelling contributes to a heavily deformed northern smooth plains on Mercury"

Jingchun Xie[1], Yan Zhan[1*], Shengxia Gong[2], Chengli Huang[2,3], and Jian Zhang[4]

[1]*Department of Earth and Environmental Sciences, The Chinese University of Hong Kong, Shatin, NT, Hong Kong SAR, China*
[2]*Shanghai Astronomical Observatory, Chinese Academy of Science, 80 Nandan Road, Shanghai, 200030, Shanghai, China*
[3]*School of Astronomy and Space Science, University of Chinese Academy of Sciences, No.19(A) Yuquan Road, Beijing, 100049, China*
[4]*Department of Earth and Planetary Sciences, The University of Hong Kong, James Lee Building, The University of Hong Kong, Pok Fu Lam, Hong Kong SAR, China*
[*]*Corresponding author: yanzhan@cuhk.edu.hk*

Introduction

This supplementary file introduces supplementary files to the main text, including three tables and nine figures.

1. Supplementary Tab.1 Model configurations for the mantle dynamic loading model
2. Supplementary Tab.2 Model configurations for 2-D numerical simulations
3. Supplementary Tab.3 Model configurations for the 3-D elastic model
4. Supplementary Fig.1 Flowchart of the fault's relief extraction processes
5. Supplementary Fig.2 The distribution of areal strains in logarithmic scale of the NSP and other terranes for various Fibonacci sampling points and dip angles
6. Supplementary Fig.3 Tectonic maps of contractional structures
7. Supplementary Fig.4 Results comparison
8. Supplementary Fig.5 Comparison of contractional strain across different fault datasets
9. Supplementary Fig.6 Mantle dynamic loading model under the assumption of fully supported by mantle dynamics
10. Supplementary Fig.7 Example of the 2-D numerical simulation
11. Supplementary Fig.8 Example of the influences of 2-D numerical simulation on surface deformation over time
12. Supplementary Fig.9 Predicted maximum principal stress orientations under the pressure loading at the Northern Rise

References

[1] Mikael Beuthe, Bernard Charlier, Olivier Namur, Attilio Rivoldini, and Tim Van Hoolst. Mercury's crustal thickness correlates with lateral variations in mantle melt production. *Geophysical Research Letters*, 47(9):e2020GL087261, 2020. doi: https://doi.org/10.1029/2020GL087261.

[2] Paul K Byrne, Christian Klimczak, AM Celâl Şengör, Sean C Solomon, Thomas R Watters, and Steven A Hauck, II. Mercury's global contraction much greater than earlier estimates. *Nature Geoscience*, 7(4):301–307, 2014. doi: https://doi.org/10.1038/ngeo2097.

[3] Fabio Crameri and Boris J. P. Kaus. Parameters that control lithospheric-scale thermal localization on terrestrial planets. *Geophysical Research Letters*, 37(9), 2010. doi: https://doi.org/10.1029/2010GL042921.

[4] M Grott, D Breuer, and M Laneuville. Thermo-chemical evolution and global contraction of mercury. *Earth and Planetary Science Letters*, 307(1-2):135–146, 2011. doi: https://doi.org/10.1016/j.epsl.2011.04.040.

[5] Steven A. Hauck II, Jean-Luc Margot, Sean C. Solomon, Roger J. Phillips, Catherine L. Johnson, Frank G. Lemoine, Erwan Mazarico, Timothy J. McCoy, Sebastiano Padovan, Stanton J. Peale, Mark E. Perry, David E. Smith, and Maria T. Zuber. The curious case of mercury's internal structure. *Journal of Geophysical Research: Planets*, 118(6):1204–1220, 2013. doi: https://doi.org/10.1002/jgre.20091.

[6] Christian Klimczak. Limits on the brittle strength of planetary lithospheres undergoing global contraction. *Journal of Geophysical Research: Planets*, 120(12):2135–2151, 2015. doi: https://doi.org/10.1002/2015JE004851.

[7] Jurriën Sebastiaan Knibbe and Wim van Westrenen. The thermal evolution of mercury's fe–si core. *Earth and Planetary Science Letters*, 482:147–159, 2018. ISSN 0012-821X. doi: https://doi.org/10.1016/j.epsl.2017.11.006.

[8] Stephan R. Loveless and Christian Klimczak. Several kilometers of global contraction on mercury: A sample-size independent assessment of fault strain. *AGU Advances*, 6(4):e2025AV001715, 2025. doi: https://doi.org/10.1029/2025AV001715.

[9] Sebastiano Padovan, Mark A. Wieczorek, Jean-Luc Margot, Nicola Tosi, and Sean C. Solomon. Thickness of the crust of mercury from geoid-to-topography ratios. *Geophysical Research Letters*, 42(4):1029–1038, 2015. doi: https://doi.org/10.1002/2014GL062487.

[10] Falko Schulz, Nicola Tosi, Ana-Catalina Plesa, and Doris Breuer. Stagnant-lid convection with diffusion and dislocation creep rheology: Influence of a non-evolving grain size. *Geophysical Journal International*, 220(1):18–36, 09 2019. doi: 10.1093/gji/ggz417.

[11] Sean C. Solomon, Larry R. Nittler, and Brian J. Anderson, editors. *Mercury: The View after MESSENGER*. Cambridge Planetary Science. Cambridge University Press, Cambridge, 2018.

[12] Nicola Tosi, M Grott, A-C Plesa, and D Breuer. Thermochemical evolution of mercury's interior. *Journal of Geophysical Research: Planets*, 118(12):2474–2487, 2013. doi: https://doi.org/10.1002/jgre.20168.

[13] Thomas R Watters. A case for limited global contraction of mercury. *Communications Earth & Environment*, 2(1):9, 2021. doi: https://doi.org/10.1038/s43247-020-00076-5.

[14] Thomas R Watters, Peter B James, and Michelle M Selvans. Mercury's crustal thickness and contractional strain. *Geophysical Research Letters*, 48(17):e2021GL093528, 2021. doi: https://doi.org/10.1029/2021GL093528.

Supplementary Tab. 1: Model configurations for the mantle dynamic loading model

Depth	Viscosity*	
0-50 km	1	
50-300 km	1×10^{-3}	
300-400 km	1×10^{-7}	
**Parameters**	**Value**	**Ref.**
Mean planetary radius (R)	2439.36 km	[11]
Radius of the core	2039.36 km	[5]
Density of the crust	2900 kg/m^3	[1]
Density of the upper mantle	3150 kg/m^3	[1]
Density of the lower mantle	3800 km/m^3	[14]
Density of the core	7000 kg/m^3	[12]
Thickness of the crust	35 km	[9]
Elastic thickness	50 km	
Mantle mass sheet loading depth	$R - 350$ km	

All viscosity values are normalized to a reference viscosity of 1×10^{23} Pa · s.

Supplementary Tab. 2: Model configurations for 2-D numerical simulations

Layers	Thick./Depth (km)	Ref.density (kg/m^3)	Ref.viscosity ($Pa \cdot s$)	Radius (km)	ΔTemp (K)
Lithosphere	160∼310	3400	$(4 \sim 100)\times10^{25}$	-	-
Mantle	260∼110	3500	1×10^{21}	-	-
Mass anomaly	350	2300∼3000	$(0.01\sim1)\times10^{21}$	18∼50	20∼60
**Parameters**	**Physical meaning**	**Values**	**Ref.**		
R	Gas constant	8.314 $J/(mol \cdot K)$	[7]		
E	Activation energy	3.75×10^5 J/mol	[3, 10]		
V	Activation volume	8.2×10^{-6}	[3, 10]		
α	Thermal expansion	2×10^{-5} K^{-1}	[7]		
T_{ref}	Reference temperature	440 K	[7]		
c_p	Specific heats	1212 $J/(kg \cdot K)$	[7]		
k	Thermal conductivity	2.0 (Lithosphere) $W/(m \cdot K)$<br>3.5 (Mantle) $W/(m \cdot K)$<br>3.5(Mass anomaly) $W/(m \cdot K)$	[4]		

Supplementary Tab. 3: Model configurations for the 3-D elastic model

Symbol	Physical meaning	Value	References
R	Mean planetary radius	2440 km	[11]
ρ	Density of the silicate lithosphere	3000 kg/m^3	[6]
g	Surface gravitational acceleration of Mercury	3.7 m/s^2	[11]
ν	Poisson's ratio	0.25	[6]
E	Young's modulus	10 GPa	
h	Average height of the load	0.5 km	

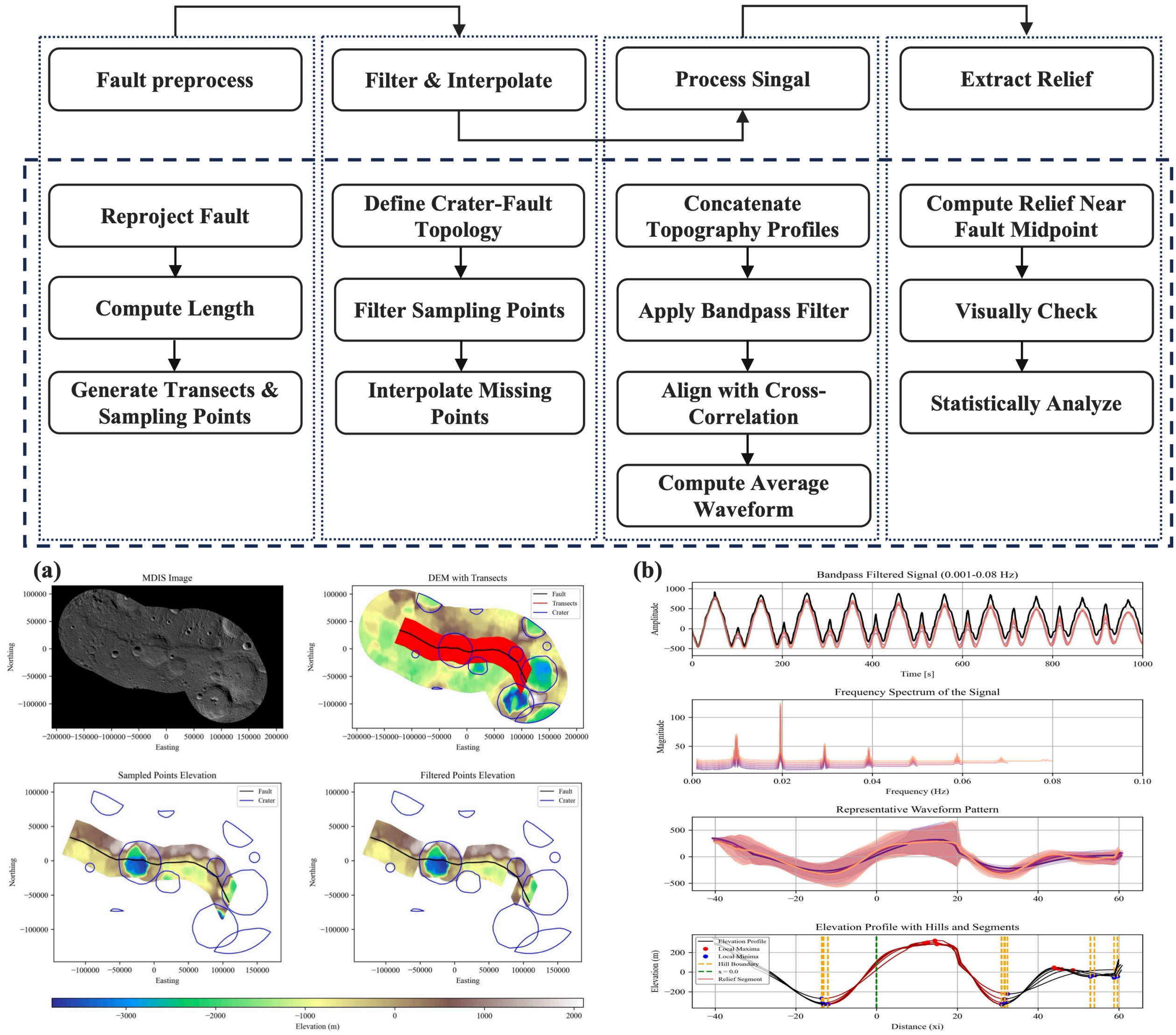

Supplementary Fig. 1: Flowchart of the fault's relief extraction processes. (a) Example of fault preprocessing and filtering. (b) Example of processing topographic profiles and fault-relief extraction.

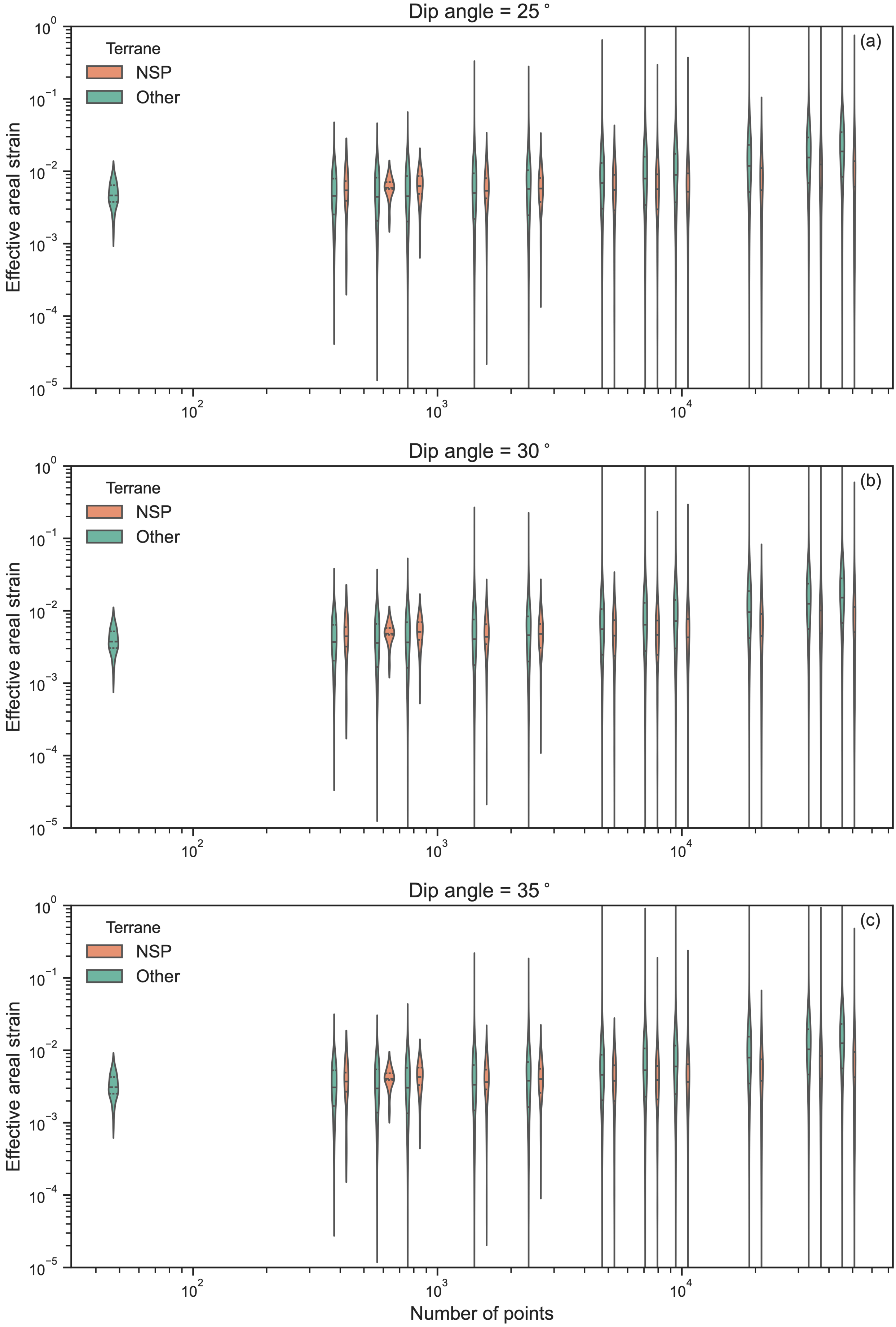

Supplementary Fig. 2: The distribution of areal strains in logarithmic scale of the NSP and other terranes for various Fibonacci sampling points and dip angles.

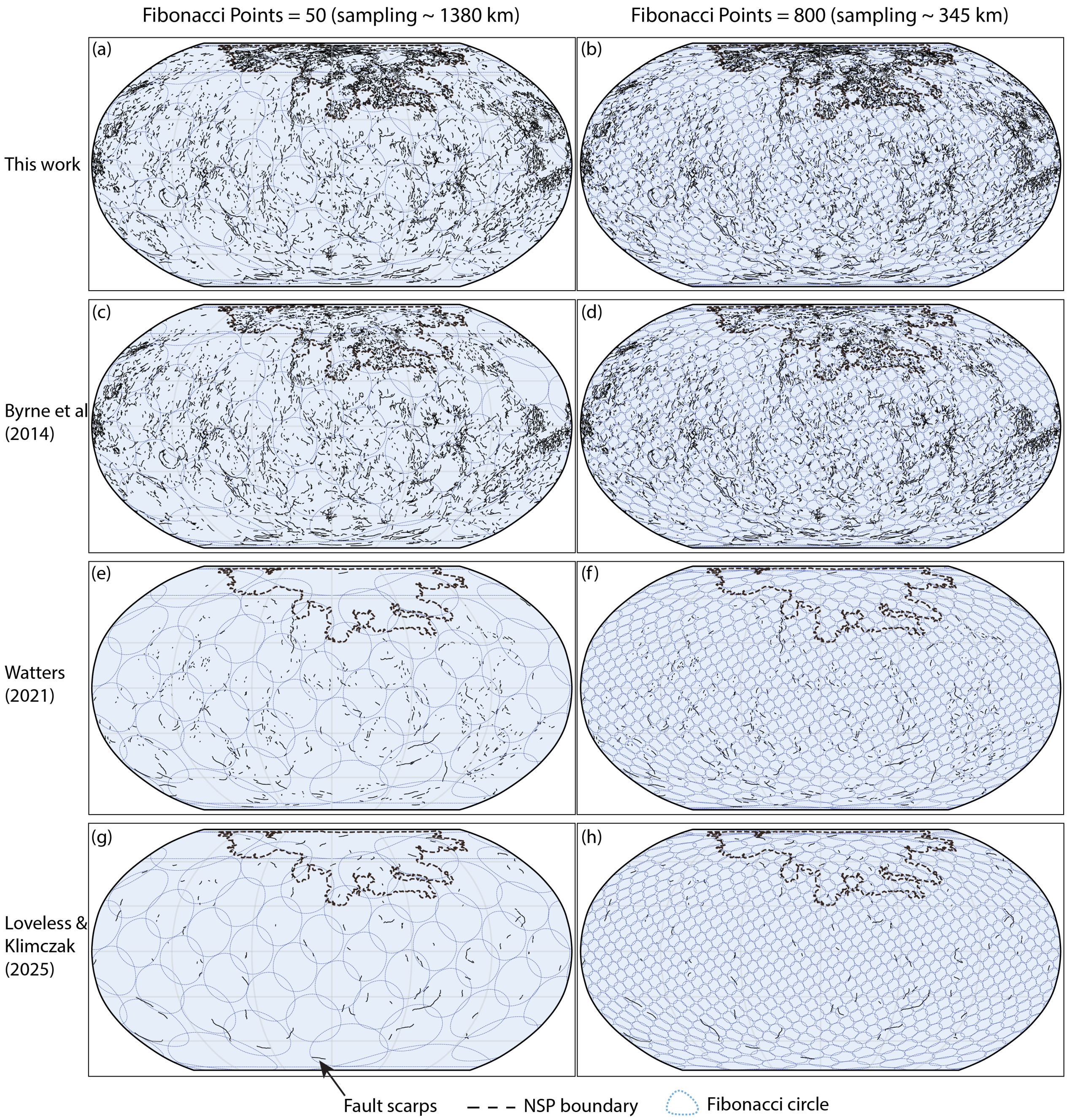

Supplementary Fig. 3: Tectonic maps of contractional structures of this research (a,b) and previous studies (c-h) [2, 8, 13], and examples of Fibonacci circles corresponding to different sampling points.

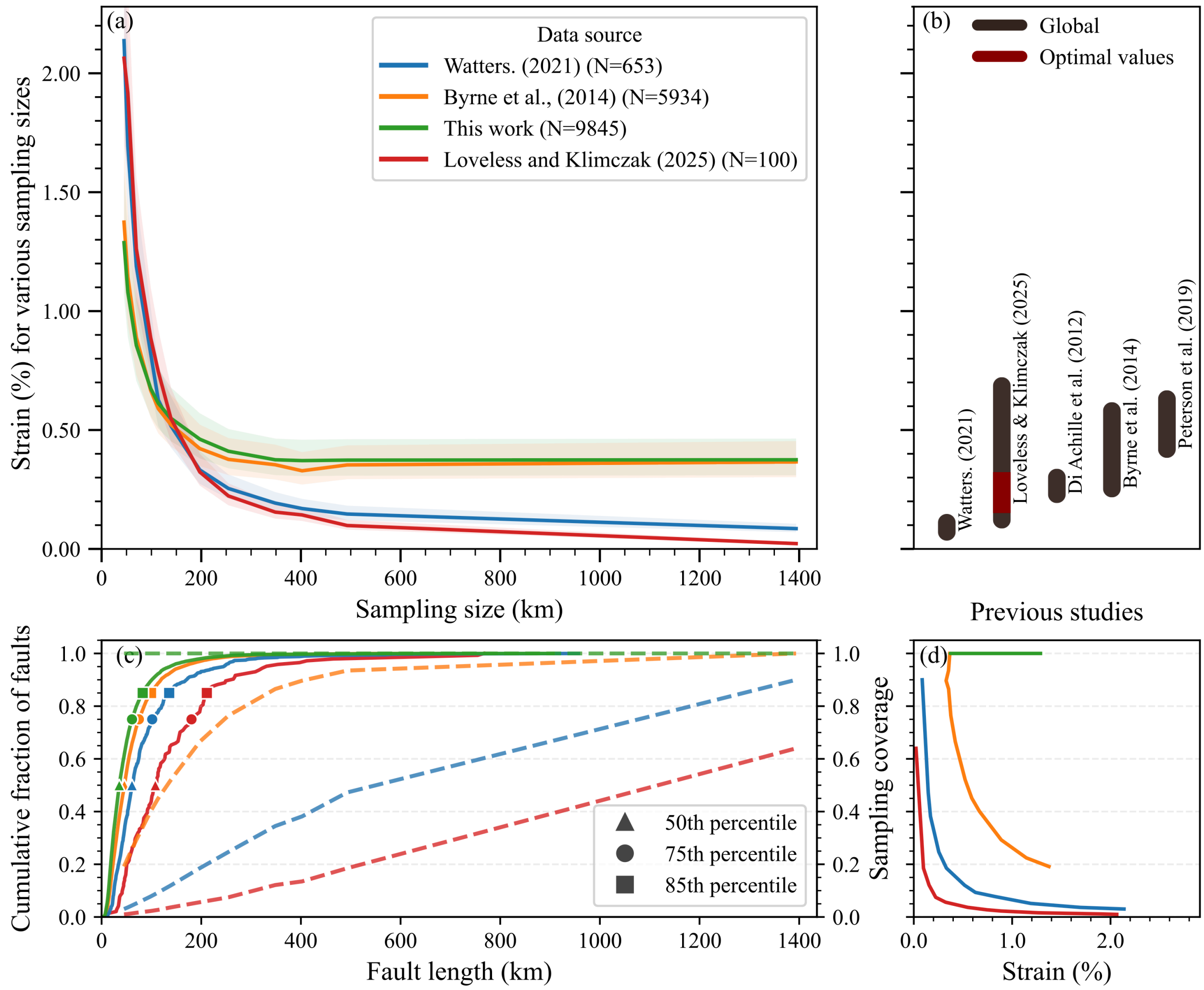

Supplementary Fig. 4: (a) Mercury's global contractional strain across different fault datasets for various sampling sizes with a dip of 30°. (b) Previous results of Mercury's global contraction. (c) Fault length distributions with sampling coverage. Step curves show the fraction of faults shorter than a given length, with markers indicating the 50th, 75th, and 85th percentiles of fault length. Overlaid dashed-lines (right y-axis) give sampling coverage–the share of sampling circles with non-zero contractional strain–plotted against sampling size. (d) Sampling coverage versus non-zero contractional strain. Each curve is constructed by evaluating the coverage at the sampling sizes that define the strain estimates shown in panel (a). This panel illustrates how sampling completeness varies with the inferred strain across different sampling sizes.

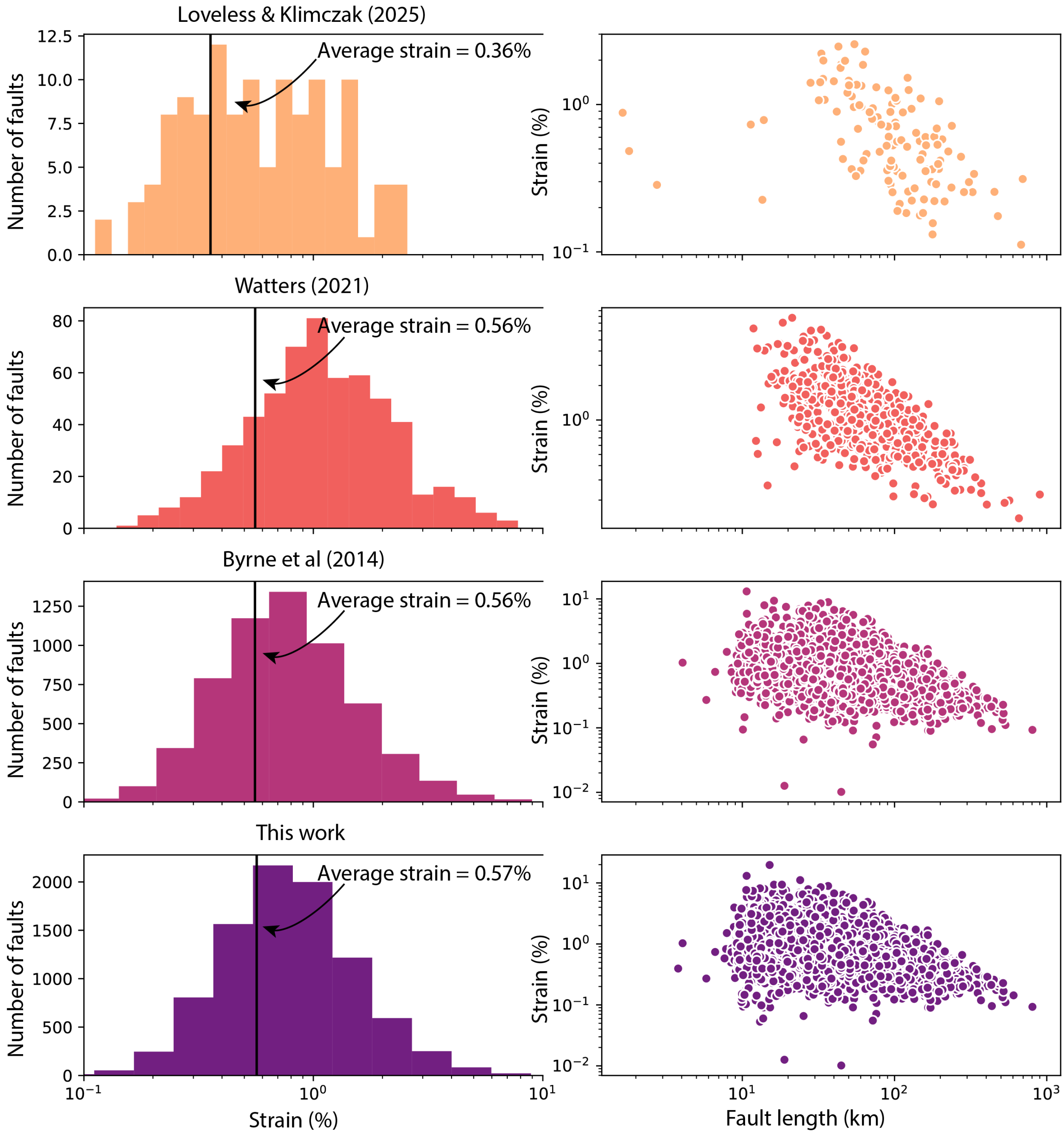

Supplementary Fig. 5: Comparison of contractional strain across different fault datasets assuming a dip of 30°. Left column: Histograms showing the distribution of strain on a logarithmic scale. The black vertical line marks the area-weighted average strain for each dataset. Right column: Scatter of strain versus fault length on logarithmic axes, with each point representing an individual fault. Contractional strain is treated as first-order estimate, calculated by $\epsilon = \frac{HL}{sin(30°)A}$, where H is the fault relief, L is the fault length. A is the faulted region for each fault, defined as $A = \pi L^2$. Accordingly, the area-weighted average contractional strain is obtained by $\epsilon_a = \sum \epsilon_i A_i / \sum A_i$.

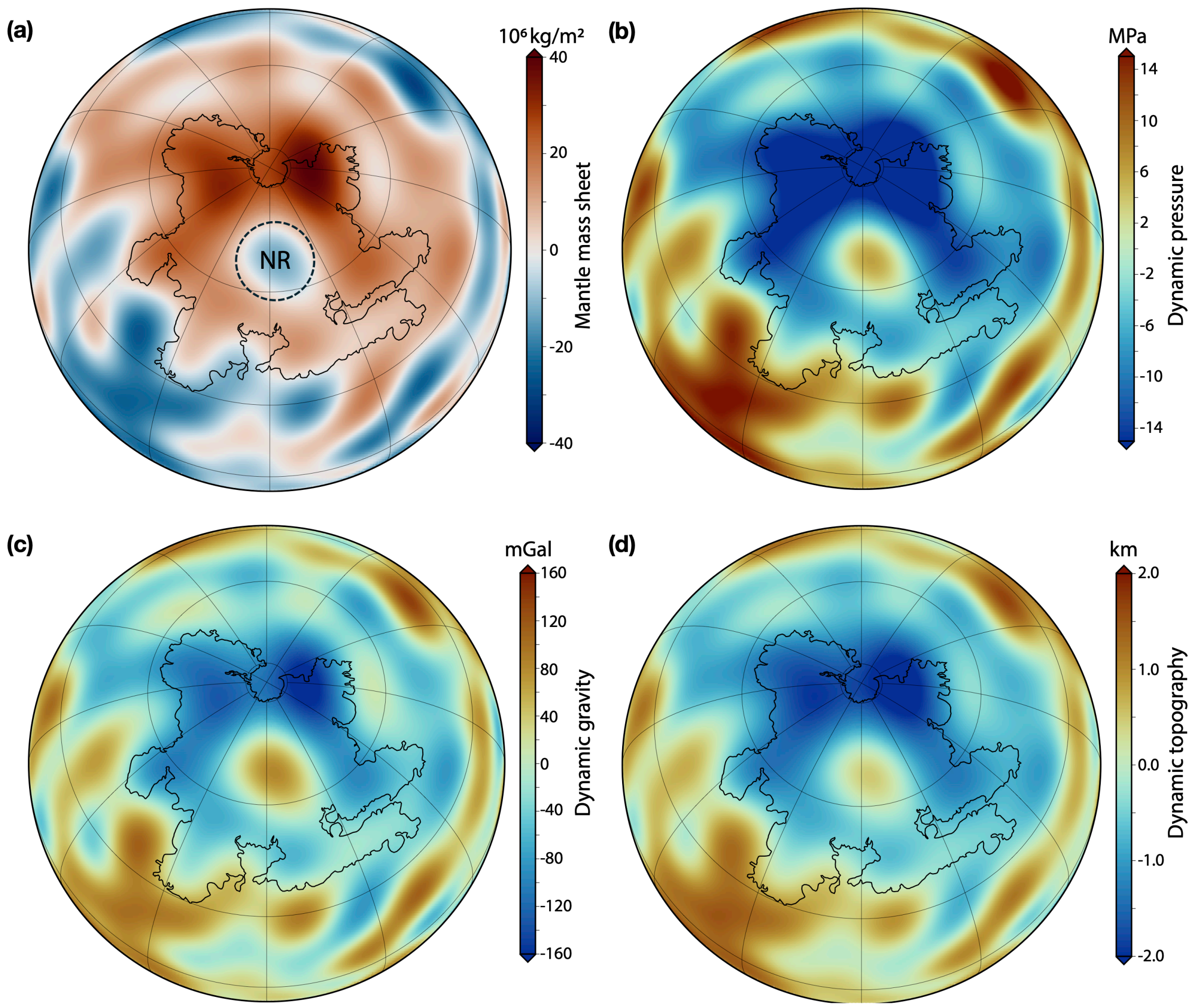

Supplementary Fig. 6: Results from the mantle dynamic loading model under the assumption of fully supported by mantle dynamics in orthographic projection centered on the Northern Rise (NR), showing (a) Mantle mass sheet, (b) Predicted dynamic pressure, (c) Predicted dynamic gravity, and (d) Predicted dynamic topography. Solid and dashed black lines respectively delineate the boundaries of the northern smooth plains and NR.

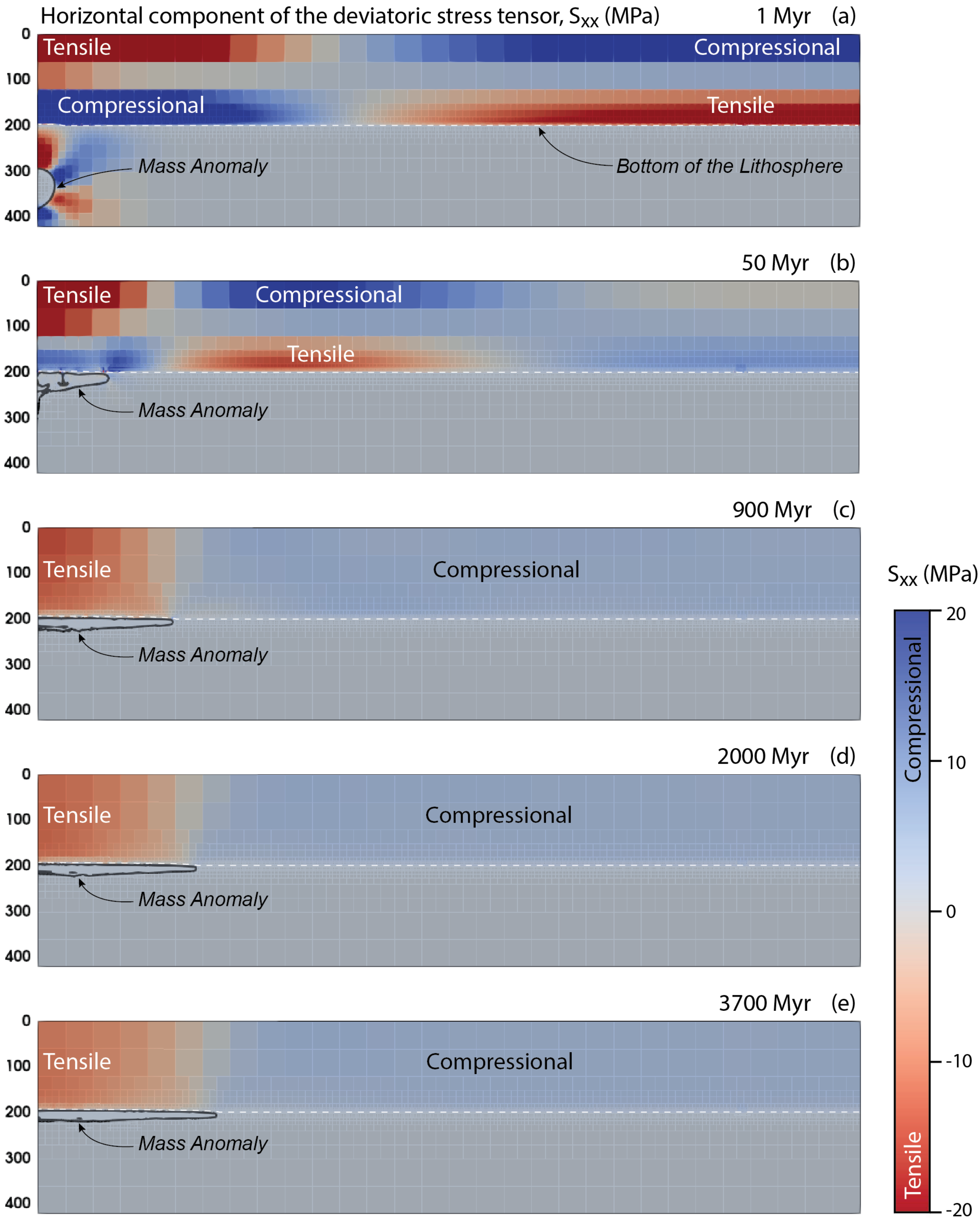

Supplementary Fig. 7: Example of the 2-D numerical simulation. This figure depicts the horizontal component of the deviatoric stress tensor (S_{xx}). The white dash line delineates the boundary between the lithosphere and mantle, while the black line outlines the contours of the mass anomaly.

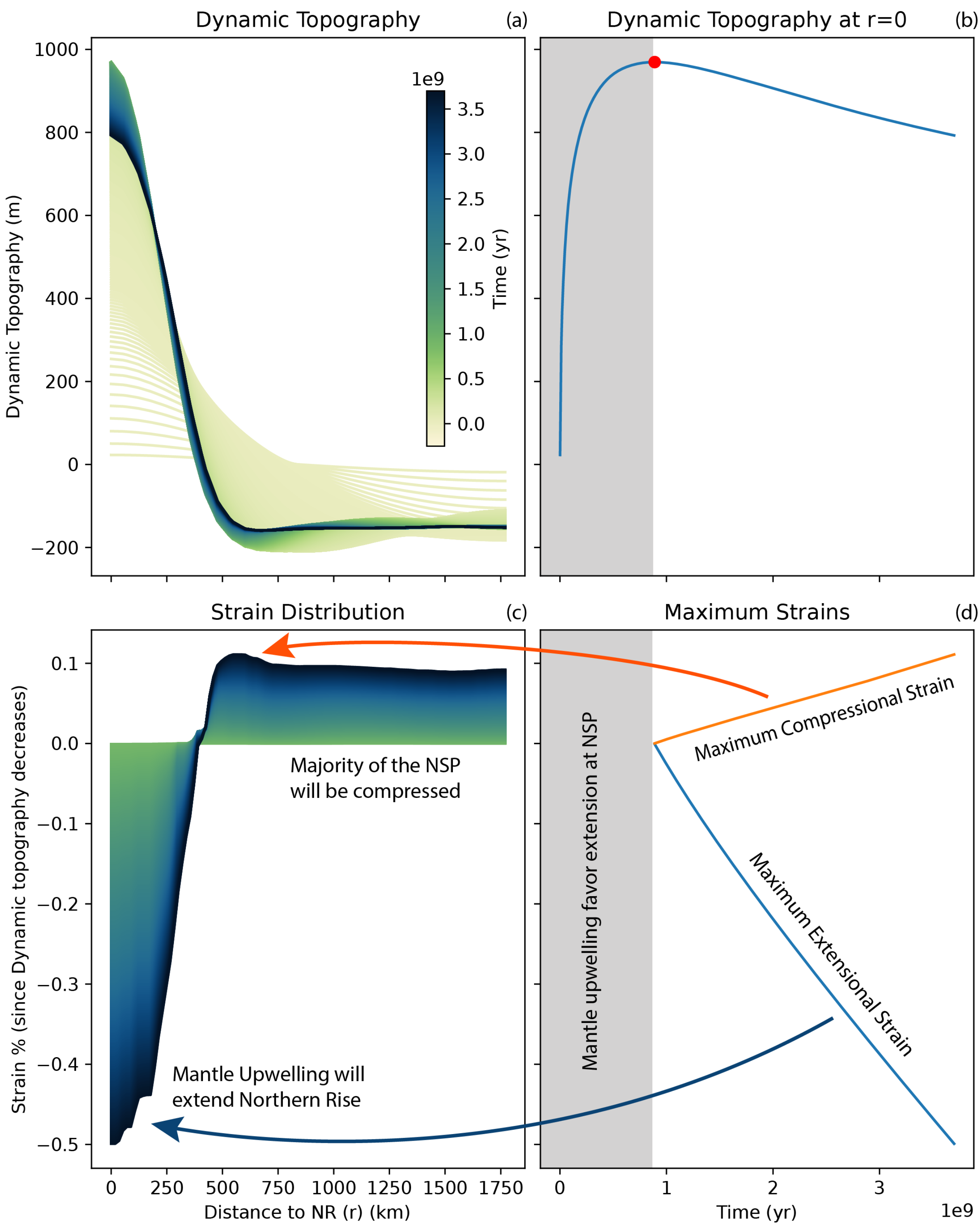

Supplementary Fig. 8: Example of the influences of 2-D numerical simulation on surface deformation over time, showing (a) Dynamic topography, (b) Dynamic topography at the distance to the NR equals zero, (c) Strain since dynamic topography decreases, and (d) Induced geological context of the NSP.

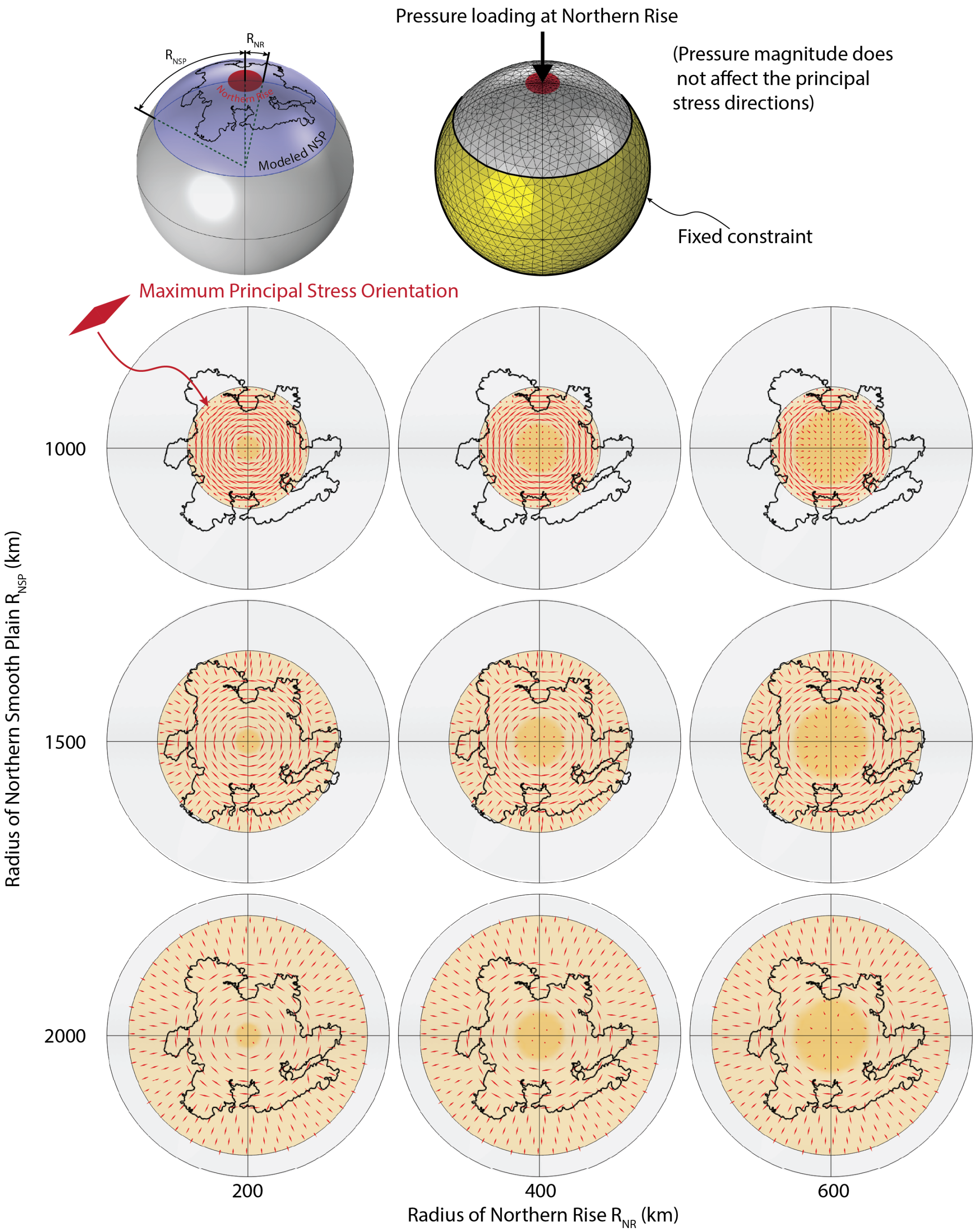

Supplementary Fig. 9: Predicted maximum principal stress orientations under the pressure loading at the Northern Rise.
