# Supplementary material for: Waned mantle upwelling contributes to a heavily deformed northern smooth plains on Mercury
Source: Nat Commun. 2026 Aug 26;17:9064. doi: 10.1038/s41467-026-74975-0 (PMC13519025; doi:10.1038/s41467-026-74975-0)
Supplement: Supplementary file 2 [file 41467_2026_74975_MOESM2_ESM.pdf]

nature portfolio

Peer Review File

Waned mantle upwelling contributes to a heavily deformed northern smooth plains on Mercury

Corresponding Author: Professor Yan Zhan

This file contains all reviewer reports in order by version, followed by all author rebuttals in order by version.

Attachments originally included by the reviewers as part of their assessment can be found at the end of this file.

Version 0:

Reviewer comments:

Reviewer #1

(Remarks to the Author)

[Editorial Note: Please see PDF at end of file]

(Remarks on code availability)
I have accessed the website which allows one to download the relevant data.

Reviewer #2

(Remarks to the Author)
This manuscript is very interesting. It presents a novel, paradigm-changing hypothesis for the tectonic evolution of NSP, a new perspective on Mercury. The authors employ a powerful combination of geophysical and tectonic modeling and observations.

I have extensive line-by-line comments below but believe the fundamental conclusions that are presented here. I think most of my comments can be addressed with minor edits to the manuscript (e.g., changes to wording, editing existing figures, paragraph organization, etc.)

I have two major comments:

- Inclusion of sample-size independent contraction estimates: The manuscript should incorporate the recently published sample-size independent contraction estimates by Loveless and Klimczak (2025), particularly in Figure 1c and the related discussion. Explicitly clarifying how these results integrate with the authors' methodology will strengthen the interpretation of NSP strain anomalies. See the line-by-line comment for lines 14 – 17.

- Visualization of strain anomalies and structural orientations: The spatial distribution of strain anomalies and the orientations of thrust faults and grabens should be visualized more clearly. Interpolated strain maps and structural orientations (e.g., structural map and even better with rose diagrams) relative to the NR would allow readers to evaluate whether the observed deformation patterns are consistent with the predicted tectonic model rather than random or controlled by other factors, such as large impacts. See the line-by-line comment for Figure 2.

Overall, it is a well-written and informative contribution to Mercury studies and deserves to be published once a few aspects are expanded upon. Thus, my recommendation is that the paper should be acceptable after pending suitable major-to-moderate revision.

Jupiter (Cheng) Hansen

Line-by-line comments:

Line 2: "offers as a unique window"

Line 7: It should be added as "tectonic" forces to be more specific

Lines 14 – 17: I totally agree with this. It is worth noting that current work estimates the 2 to 3.5 km of radial contraction using a sample-size independent model, resulting in a lower upper bound strain than the previous sample-size dependent models (i.e., 7.6 km by Watters 2021 and 8.4 km by Byrne et al. 2014).
Loveless, S. R., & Klimczak, C. (2025). Several kilometers of global contraction on Mercury: A sample-size independent assessment of fault strain. AGU Advances, 6(4), e2025AV001715.

Line 22 – 27: It is a very long sentence with a lot of information. I suggest rewriting it into two like: Previous studies have proposed volcanic loading as an additional driving mechanism [5, 8]. However, the pronounced spatial clustering of contractional landforms in the NSP [7, 16] is inconsistent with the expected uniformity of deformation from volcanic loading, as is the absence of significant spatial variations in lava flow thickness [17], which would be necessary to generate localized loading and associated tectonic deformation. Also, I suggest starting as a new paragraph with "Previous studies…"

Lines 59 – 78: The logic is solid, but the paragraph organization is somewhat unclear due to the mixing of multiple ideas in a single block. I suggest keeping the first paragraph from "Our results indicate that the contractional …" through "across different sampling sizes." Then starting a new paragraph with "This interpretation assumes that scarps in the NSP and other terranes have the same average dip angle."

Figure 1: This is a strong and well-designed figure, but a few aspects could be improved.
(1) Because the figure is intended to demonstrate the dependence of estimated contractional strain on sampling size, it would be helpful to include the sample sizes used in previous studies. In panel (c), strain estimates from earlier work are ordered from small to large, but their corresponding sample sizes are not shown. For example, Byrne et al. (2014) analyzed 5,934 shortening landforms, whereas Watters et al. (2021) considered 653. Displaying these values, or another quantitative measure of sampling size, either on the bars or along the x-axis would provide important context for interpreting the differences among studies.
(2) Several previous studies focus on localized regions rather than the entire "other terrane." For instance, Di Achille et al. (2012) covers approximately 21% of the planetary surface. Outlining the study areas of each prior work in panel (a), while retaining the full "other terrane" in blue, would help clarify whether differences in estimated strain also reflect study area selection. This would also avoid the potential misinterpretation that all published strain estimates represent the entire "other terrane."
(3) As noted in my comments for line 14-17, the sample-size independent result from Loveless & Klimczak (2025) should be included in the figure and related main text.
(4) It is probably me being nitpicky but the max in "Dmax" looks like the same size as D in the block diagram in panel (b) when it should be smaller since it is in subscript.

Figure 2: The statement in line 102 that "the strain anomaly demonstrates a concentric distribution of strain, with negative anomalies at the central area (NR) and positive anomalies outward" is plausible, but this pattern is difficult to evaluate from Figure 2 as currently presented. An interpolated strain map would help more clearly visualize this spatial distribution. Because the concentric pattern is central to the manuscript's interpretation, explicitly illustrating it would substantially strengthen the argument.

Line 99 – 101: Related to the above point, impact-related tectonism may be a major reason the strain pattern appears less clear. To test this interpretation, it would be useful to plot thrust fault scarps and grabens across the NSP using the mapping data already shown in Figure 1a, ideally overlain on the interpolated strain map suggested above. This would allow readers to assess whether strain anomalies are indeed disrupted near large impact structures and to evaluate the relationship between shortening and extension versus positive and negative anomalies.

More importantly, such an approach could also illustrate the orientations of these structures relative to the central NR area. As discussed later in the manuscript (particularly in Figure 6), the waned mantle upwelling hypothesis predicts not only higher deformation concentrated in the NSP but also preferred deformation orientations with respect to NP. Specifically, extension (Figure 6a) and compression (Figure 6b) are expected to radiate outward from NP, producing grabens and thrust faults concentric around the center. I would even suggest plotting rose diagrams projected around the center of the NSP, if possible, to directly visualize these orientation patterns. Providing this visualization and analysis is essential to demonstrate that the observed deformation is not simply controlled by variations in crustal properties or global contraction, which would more likely produce random orientations, and to ensure that the spatial distribution within the NSP is not biased by large impacts near its margins.

To accommodate the additional visualization, it may be preferable to show only the 50 km bin-size panel in the main figure and place the 25 km and 100 km panels in the Extended Data, particularly since the differences mainly reflect local variability, as noted in the manuscript. The final choice of presentation, of course, remains with the authors.

Caption of Figure 3: "Results from the mantle dynamic loading model in orthographic projection centered on the Northern Rise (NR) (30∘E, 70∘N), where (c) Mantle mass sheet, (d) Predicted dynamic topography, (e) Predicted dynamic gravity, and

(f) Predicted dynamic pressure." | This sentence is ungrammatical, as "where" cannot be used to introduce a list of panels without a verb. Replacing "where" with "showing" would resolve this issue.

Lines 141 – 142: "Therefore, we largely expand the parameters space configuring numerical simulations." | The phasing in this sentence is unclear and unidiomatic. Perhaps rewrite it as: "Therefore, we consider a wide range of parameters used to configure the numerical simulations."
Lines 149 – 150: Revise this phase as "followed by gradual subsidence after several hundred million years"

Figure 4: The figure caption is somewhat confusing, as panel (b) includes a thick blue line labeled "Model" as well as a blue-to-green color scale representing maximum extensional strain. I assume these two elements refer to the same model. If so, I suggest removing the thick blue line from the legend and instead labeling the color scale as the model output. If I have misunderstood and the thick blue line represents a different model, then using a color distinct from blue would help avoid confusion. In either case, additional clarification in the figure caption would be helpful.
Line 169: The "–" is a bit awkward here. I think it is better to use commas here and same as in line 170, such as "… parameter, the pseudo-Rayleigh number (pRa; Fig.5), which…"
Line 178: The sentence reads awkwardly and appears to be a sentence fragment. Please rewrite for clarity.

Section 6: I think this section (and probably section 5 as well) contains substantial methodological detail, much of which is described elsewhere in the Materials and Methods section. I suggest referring to Materials and Methods section when suitable in these sections.
Figure 6: It is a very nice schematic illustration.

However, the relative thickness of the lithosphere and mantle appear similar in some locations, which makes the figure look slightly odd. I suggest making the lithosphere thinner than the mantle, especially given the relatively thin lithosphere assumed in this study. The figure could also be more tightly zoomed into the lithosphere–mantle region, as the core occupies nearly half of the illustration but is not the focus on this study.

Also, seeing the predicted tectonic orientations in this illustration makes me feel strongly that a map showing the observed structure projected in NSP is needed on top of the spatial distribution of strain anomalies in Figure 2. See my comments for lines 99-101.

Line 212: I suggest replacing "–" with ";"

Line 215: I suggest deleting "Then," and starting the sentence with "A key question is whether…", which would strengthen the statement.

Line 217 – 221: What could explain why the proposed large impact produces results so different from those of the Caloris basin? One or two sentences of justification would be useful here.

Methods (Lines 235 to 402): I do not have any major comments or suggestions for this section and the extended data, only minor typos (see below). As mentioned in Section 6, this section should be referenced in the main text, particularly where the results rely on the methodology.

Line 351: "we" should be "We"?

Line 371: "we have constructed" should probably be "we construct" to keep it consistent with present tense throughout the method section.

Line 540: What is the "???" in the reference?

Line 612: This reference seems incomplete.

(Remarks on code availability)

Version 1:

Reviewer comments:

Reviewer #1

(Remarks to the Author)
I have read the revised manuscript titled Wanted mantle upwelling contributes to a heavily deformed northern smooth plains on Mercury by Xie et al. In this manuscript, strain, gravity, and structural observations are used to determine that global contraction by itself, is insufficient to explain the unique structural anomalies that occur on the Northern Smooth Plains on Mercury. The manuscript describes a mantle upwelling model which demonstrates that the regional deformation of Mercury's NSP can be explained due to an underlying mantle upwelling.

I am pleased to share that the authors of this manuscript did a phenomenal job with their revisions. Their reasoning, and justification for their research is clearly stated, and the manuscript flows very well, allowing the reader to anticipate the research objectives of each subsequent section. The results of their work have a substantial impact on the Mercury tectonics community. For these reasons, I believe this manuscript should be accepted for publication following taking into account minor revisions (listed line-by-line below).

Best,

Dr. Stephan Loveless

Introduction

Line 10: change “as the product” to “to be the product”

Line 15: change “commonly used computational method extrapolates” to “commonly used computational methods extrapolate”

Line 18: true Loveless and Klimczak (2025) found lower values of radial contraction than Byrne et al. (2014) did using “optimal physical parameters”, but these parameters were relatively conservative. I am not sure how strong of an argument can be made in this respect.

Stronger contractional strain in Mercury’s Northern Smooth Plains

Line 51: I question a bit the choice to use the word “strong” with strain since strain is not necessarily a force, or a parameter that is related to force. Perhaps “greater contractional strain” would be more appropriate.

Extended Data Figure 2 is a little confusing to read since the “other” contractional landforms are flipped on the “number of points access”. Provided that this is a histogram and that a logarithmic scale is being used, a different display would be better. Maybe an overlay. Of the two histograms in the same direction.

Figure 1c) (and others) it is implied with how the figure is portrayed that the data set with 100 shortening landforms was the optimal data set for Loveless & Klimczak (2025), or Loveless & Klimczak (2025) produced the 100 shortening landform data set, which is incorrect. Loveless & Klimczak used optimal parameters on all 3 data sets used in the paper to provide “most-likely conservative estimates” for Mercury’s radial contraction.

The work from Loveless & Klimczak (2025) did not have a data set of just 100 shortening landforms. Instead, it calculated global contraction using 3 different data sets each with different sample sizes of contractional landforms: 1) Byrne et al. (2014) (n ≈ 6000),
2) Watters (2021) (n ≈ 600), and Loveless et al., (2024, 2025) (n = 100).

Line 96: I would hesitate in using the words that claim that the data set is “ours” as this work applied a methodology on a data set captured by Klimczak et al. (2025). Perhaps instead of “ours” use the term “the data set utilized here”

Line 115: The difference in strain partitioning from lobate scarps and wrinkle ridges was not a result of Loveless & Klimczak (2025). In Loveless et al (2025) (the fault modeling paper) there was an overlap in the folded partitioned shortening strain, indicating that perhaps the difference between lobate scarps and wrinkle ridges were just the size of the shortening landform. It was also a result that lobate scarp shortening landforms accumulated more strain from folding than wrinkle ridges, but this did not account for how the strain was partitioned, it just was that lobate scarps had more strain likely because they were larger structures and were therefore faults that had matured longer.

Loveless et al. (2025) calculated the modeled strain from folding as well as modeled the heave from the fault, but how these were partitioned differently from lobate scarps and wrinkle ridges was not a result of this paper. There may be some suggestions of differences in fault dip, but the other statements of this sentence were not results of these papers. In particular, none of these statements are related to the results of the global contraction estimate paper form Loveless & Klimczak (2025).

Lines 121-125: true, but if there were pre-existing faults underneath the smooth plains that continued to grow slowly (as suggested by the basement-activated thin-skinned tectonics of Loveless et al. (2025)) then contraction may still be present. Perhaps the work presented here works in conjunction with this model?

Controls on the upwelling-induced surface deformation

Figure 5b – perhaps this caption should be part of the methods section.

Tectonic evolution of the NSP and its implications to Mercury’s interior

Paragraph between 342-347: conceptually, could it also be possible that there was early mantle upwelling that has since locked in place, causing a topographic high that of which stresses from global contraction coagulated on/around? Similar to the localization of stresses around buried crater rims?

Methods

Expression of contractional strain

Line 392: add the word “be” between the words “to” and “related”. And add the word “to” between the words “related” and “D”. The sentence should read: “…a scaling factor (γ) to be related to D and L…”

Line 392: it is a little confusing if the elliptical fault geometries that is typically observed is applied to all of the faults in this analysis as it is discussed after small scale faults. Is this taken into account for all faults or just small structures? If just small structures, why?

(Remarks on code availability)
I have accessed the website which allows one to download the relevant data.

Reviewer #2

(Remarks to the Author)
I want to thank the authors for making the effort in revising the manuscript. It was very good to see the improvement during the revision. The answers to my comments, questions and requests for small additions were certainly adequate. A minor justification is still essential with the new Fig. 2 (see comment for line 155).

Below are the line-by-line comments where I highlighted a few remaining typos or slightly awkward sentences. From my perspective, after adding the justification and fixing the few remaining typos or wordings, this is essentially good to go.

Best regards,
Jupiter (Cheng) Hansen

Line-by-line comments:

Lines 15-16: Did you mean "extrapolates strain across the study area from"? "To" implies the strain is being projected into the region rather than calculated from structures within it.

Lines 95-96: “Although a difference of nearly 4,000 mapped faults between our dataset and that of Byrne et al….” reads awkwardly. The sentence starts with "Although" but lacks a proper contrast clause. Probably rewrite it as “"despite a difference of nearly 4,000 mapped faults..."

Line 155: Instead of “perpendicular to the radial”, may be just directly say concentric? Unless I totally misunderstood the meaning of the strike direction. Also, it seems that the far-field faults match the elastic model prediction, but the proximal ones do not. Why is that? This is worth a sentence of clarification.

Fig 2 caption: “anomaly versus Fault orientation pattern” The “fault” should not be capitalized.
Line 343: The phasing “later in its history than is often assumed because its relatively thin thickness” is awkward and incomplete. Should probably be something like "because of its relatively thin (crustal?) thickness"

Line 366: “reorganized the regional stresses field” should be “reorganized the regional stress field”

Line 451: “are then subjected statistical analysis” should be “"are then subjected to statistical analysis"

Line 466: “In this cases” should be “In this case” as singular

Line 496: “in this cases” should be “in this case”

(Remarks on code availability)

Open Access This Peer Review File is licensed under a Creative Commons Attribution 4.0 International License, which permits use, sharing, adaptation, distribution and reproduction in any medium or format, as long as you give appropriate credit to the original author(s) and the source, provide a link to the Creative Commons license, and indicate if changes were made.
In cases where reviewers are anonymous, credit should be given to 'Anonymous Referee' and the source.
The images or other third party material in this Peer Review File are included in the article's Creative Commons license, unless indicated otherwise in a credit line to the material. If material is not included in the article's Creative Commons license and your intended use is not permitted by statutory regulation or exceeds the permitted use, you will need to obtain permission directly from the copyright holder.
To view a copy of this license, visit https://creativecommons.org/licenses/by/4.0/

Response to Reviewer Comments:

Waned mantle upwelling contributes to a heavily deformed northern smooth plains on Mercury

Text in black: Reviewer comment/suggestion/question

Text in blue: Author response

Reviewer #1 (Dr. Stephan Loveless):

I have read the manuscript titled Waned mantle upwelling contributes to a heavily deformed northern smooth plains on Mercury by Xie et al. In this manuscript, the areal strain from thrust fault-related landforms was found to be unique in the Northern Smooth Plains, particularly around the Northern Rise. Mantle upwelling was then modeled as a way to explain the existence of the Northern Rise, and as a way to account for the strain anomaly in this area. I had found the results of this manuscript very interesting and relevant and thus warrants consideration for publication. However, there were many aspects of this manuscript, especially in the methodology, or justification of methodology, that had substantial holes that required further explanation. For this reason, I believe that the manuscript should be potentially reconsidered for publication following major revisions.

I have provided an in-depth review below, starting with general comments, followed by line-by-line comments. I have tried to be as constructive and helpful as possible.

Thank you very much for your careful and thoughtful review of our manuscript. We sincerely appreciate the time and effort you invested in providing such a detailed and constructive assessment. We are encouraged that you found the study interesting, relevant, and potentially worthy of publication. At the same time, we fully acknowledge your concerns regarding aspects of the methodology and the justification for some of our approaches. Your comments have been very helpful in showing us where the manuscript was unclear or insufficiently supported.

General Comments:

1). It is hard to tell what exactly the methodology is. In a Nature formatted paper, the detailed methods come at the end, but nearly no information is provided as to what is done so that the reader distinctly understands the goal of the paper. Aspects like, preferred models (figures 4 and 5), or the reliable strain estimates referenced in the section titled Stronger Contractional Strain in Mercury's Northern Smooth Plains, are not defined, sometimes even in the methods section, leaving the reader to wonder what is truly being used to constrain the models, a crucial point of the paper. I suggest that the organization of the manuscript be reviewed as at this stage it feels that the main text relies on the reader jumping to the methods at the end of the paper too much.

Thank you for this important comment. We agree that the method remains necessarily technical, but we have tried to make the main logic readable without requiring the reader to move immediately to the Methods. In response, we revised the main text to make the workflow more accessible in the main text by summarizing the three main steps of the analysis, explaining the equal-area sampling strategy, and clarifying the rationale for the adopted fault-length threshold and convergence tests.

Specifically, we now introduce the strain-calculation approach in the main text before presenting the interpretation. In brief, contractional strain is estimated by sampling Mercury's surface with a Fibonacci-sphere scheme, in which evenly distributed sampling circles of different sizes are used to calculate the weighted cumulative fault length per unit area. We also explain that strain estimates are evaluated as a function of sampling size, and that the large-scale, stable results are used to characterize the background contractional strain.

We further clarify two terms that were insufficiently defined in the original manuscript. First, by "reliable strain estimates," we mean strain values obtained at sampling sizes for which the estimates become stable with increasing sampling size. Second, we clarified in the manuscript that our "preferred models" are those that reproduce both the wavelength and amplitude of the Northern Rise topography together with the observed radial contraction pattern. This definition is now stated explicitly near the model–data comparison.

To improve readability, we have also revised the organization of the section *Stronger contractional strain in Mercury's northern smooth plains* so that the methodological context is presented before the main results and interpretations, while the full technical details remain in the Methods and Extended Data. We provide additional technical discussion of the strain-calculation framework, including comparisons with other fault datasets, in our responses to comment #16.

2). More generally, the results presented in the section Stronger Contractional Strain in Mercury's Northern Smooth Plains, are simply just stated, without any succinct, general comments about what was actually done in the study. The reader should have a general idea of the methodology without getting into the details presented in the methods section.

Thank you for this helpful comment. We agree that the original version of the section *Stronger contractional strain in Mercury's northern smooth plains* presented the main results too directly, without first giving readers a sufficiently clear overview of what was done. In the revised manuscript, we have therefore added a short methodological summary to the main text so that readers can understand the basis of the analysis without needing to rely immediately on the Methods section.

Specifically, we now explain in the main text that contractional strain was estimated by sampling Mercury's surface using a Fibonacci-sphere approach, in which evenly distributed sampling circles of different sizes are used to calculate the cumulative weighted fault length per unit area. We also clarify that the strain estimates are evaluated as a function of sampling size, and that the median strain at sufficiently large sampling sizes is used to characterize the background level of global contractional deformation. In addition, we now define more clearly what is meant by

"reliable strain estimates" and how these estimates are used to constrain the preferred models shown in Figs.4 and 5.

To improve readability, we have also revised the organization of this section so that the methodological context is introduced before the main interpretations, while the full technical details remain in the Methods and Extended Data. These revisions are described in more detail in our responses to comments #14–20.

3). Stress and strain terms are used intermixed throughout the paper. Extension and contraction are strain terms. Tension and Compression are stress terms. The authors calculate both stress and strain throughout the paper, but it is unclear what is being referenced to and where. Furthermore, the signs for stress and strain must be defined as they can be different depending on the methodology. For example, is compression positive or negative? Is contraction positive or negative? How does this play a role in the calculation of the strain anomaly in the Northern rise?

Thank you for this important comment. We agree that the original manuscript did not distinguish stress and strain terminology clearly enough, and that the sign conventions were not explicitly defined. In the revised manuscript, we have corrected the misuse of terms throughout the main text and figures so that **compression/tension** are used only for **stress**, whereas **contraction/extension** are used only for **strain**.

We have also added an explicit statement of the sign conventions used in the study. In our formulation, **compressive stress is positive** and **tensile stress is negative**; similarly, **contractional strain is positive** and **extensional strain is negative**. We now state this clearly in the manuscript where stress, strain, and anomaly calculations are introduced.

In addition, we clarify how this convention is applied in the calculation of the strain anomaly in the northern smooth plains. Specifically, the anomaly is defined relative to the average contractional strain, so that **positive anomaly values indicate stronger contraction than the average level**, whereas **negative anomaly values indicate weaker contraction or relatively extensional behavior**. These revisions should make the terminology and interpretation much clearer throughout the paper.

4). Additionally, lower dip angle does not physically mean that a fault experiences more contraction, an argument this paper makes with respect to shortening landforms in the northern smooth plains. This is an artifact of the previous method used to estimate Mercury's global contraction when just comparing a simple-shear fault geometry to the relief of a shortening landform. The correlation between the two are an artifact of this equation. Loveless & Klimczak (2025) in AGU Advances had found greater estimates for radial contraction with steeper dip angles.

Thank you for this important comment. We agree that the original wording could have suggested an overly direct physical link between lower fault dip angle and greater contraction, which was not our intention. In the revised manuscript, we now clarify that the dependence of inferred

contraction on dip angle in our analysis is a consequence of the geometric assumptions built into the strain-estimation method, rather than a general physical relationship.

We also revised the discussion to acknowledge more fully the results of Loveless & Klimczak (2025), which demonstrate that alternative treatments of fault geometry and deformation partitioning can lead to different dip angle–contraction relationships. This highlights that such relationships are model dependent and should be interpreted with caution.

Accordingly, we have moderated the relevant text and now present the adopted dip angles (25, 30, and 35 degrees) as endmember assumptions used to evaluate the sensitivity of our results. Our main conclusions are based primarily on the relative spatial pattern of contractional strain and the stability of the large-scale sampling results, rather than on the absolute contraction value associated with any single dip-angle assumption.

5). The relationship between fault dip and displacement, is sort of a chicken-before-the-egg argument. Provided one trigonometric relationship, it has been widely accepted that low dip = greater displacement and therefore, greater strain. However, just because a fault has a low dip, does not guarantee that it has larger displacement. Displacement is more so correlated and physically aligned with the size of the fault (i.e., length). A fault will mature, grow in length, and continue to experience displacement. Other factors will influence the dip of the fault but does not necessarily guarantee that a fault with low dip means you have a large amount of displacement.

Thank you for this helpful comment. We agree that the relationship between fault dip and displacement should not be presented as a simple one-to-one physical correspondence. In particular, a lower dip angle does not by itself require a larger displacement, and displacement is influenced by the broader structural evolution of a fault, including its growth, geometry, and deformation history.

In the revised manuscript, we have therefore moderated our wording to avoid implying that low dip necessarily means greater displacement or strain. We now clarify that, within our analysis, dip angle enters as a geometric assumption in the strain-estimation framework rather than as a direct predictor of fault displacement. We also acknowledge that fault displacement is more generally related to the development and scale of the structure, and that dip angle alone is not sufficient to determine the amount of shortening.

Accordingly, we treat the adopted dip angles as endmember assumptions used to test the sensitivity of the strain estimates, and we focus our interpretation primarily on relative spatial variations in contractional strain rather than on a strict physical inference from dip angle alone.

6). On a last note, this paper is focused on the northern smooth plains but does not include its formal name throughout the entire manuscript: the Borealis Planitia.

Thank you for this helpful comment. We agree that the formal name of the northern smooth plains should be introduced clearly and used consistently in the manuscript. In the revised

version, we now identify the northern smooth plains as **Borealis Planitia** at first mention and have revised the text accordingly to ensure the terminology is clear throughout.

Specifically, the opening paragraph now reads:

Mercury, the smallest and innermost planet in our solar system, offers a unique window into planetary tectonics and thermal evolution. Among its geological features, the northern smooth plains (NSP), formally named Borealis Planitia, stands out as a vast volcanic expanse covering approximately 7% of Mercury's surface [1–3]. Dated to about 3.7 Ga [4], the NSP preserves well-defined contractional landforms such as lobate scarps and wrinkle ridges [5,6], making it a key area for understanding the tectonic forces shaping Mercury's surface over time.

The rest of the comments are line by line notes:

Introduction

7). Line 1: get rid of the word "as". Should read as "… offers a unique window…"

Thank you for catching this. We agree and have removed "as" so the sentence now reads, "...offers a unique window...".

8). Paragraph one is missing some key information. Mainly the second sentence is quite broken up when it doesn't need to be & terms like "relatively" young and "tectonic" structures are not provided good examples.

Thank you for this helpful comment. We agree that the opening paragraph needed clearer context and more concrete examples. We have therefore rewritten it to improve the flow, clarify the age and significance of the northern smooth plains, and provide specific examples of the contractional landforms discussed in the paper.

The revised paragraph now reads:

Mercury, the smallest and innermost planet in our solar system, offers a unique window into planetary tectonics and thermal evolution. Among its geological features, the northern smooth plains (NSP), formally named Borealis Planitia, stands out as a vast volcanic expanse covering approximately 7% of Mercury's surface [1–3]. Dated to about 3.7 Ga [4], the NSP preserves well-defined contractional landforms such as lobate scarps and wrinkle ridges [5,6], making it a key area for understanding the tectonic forces shaping Mercury's surface over time.

9). Line 9: this may be a bit pedantic, but grammatically, it would read better as "thrust fault related, scarps" as opposed to "thrust-fault scarps".

Thank you for this suggestion. We have revised the text accordingly and replaced "thrust-fault scarps" with "thrust fault-related scarps."

10). Line 18: it is more appropriate to use the term “contractional” than “compressive” as deformation is what is being described (i.e., strain). Compression is a stress not a strain term. Same with line 19, line 55, many of the figures. Extensional features (i.e., normal faulting) form in compressive stress regimes, it is only the configuration of principal stresses that differs between contractional (thrust faulting) and extensional (normal faulting) strain domains. (this is referred to correctly in line 32)

Thank you for pointing this out. We agree that “contractional” is the appropriate term when referring to deformation (strain), whereas “compressive” refers to stress. In the revised manuscript, we have corrected the terminology throughout the main text and figures to distinguish stress and strain more consistently.

11). Line 21: I would suggest removing “on the other hand” as it is later stated “if the latter stands” making referencing the alternate possibility redundant. Furthermore, the grammar of this sentence is incorrect. Perhaps reword to “If the latter is true, one must then ask, ‘what might these mechanisms be?’.

Thank you for this helpful suggestion. We have therefore removed “on the other hand” and rephrased the sentence for clarity.

12). Line 22 to 27: this is an extremely long, run-on sentence. This should be separated into 3 or 4 different sentences.

Thank you for your suggestion. We have started a new paragraph with “Previous studies” and broken this sentence into several different sentences.

13). Line 27: it is written that “Most importantly, the volcanic loading is unlikely to support sustained geological activity” but is not suggested why that may be. I am assuming that there are two primary reasons why this is being suggested: first, lithospheric flexure from loading on Earth predominately forms extensional features, so we would not expect contraction. Second, Mercury is cold, its mantle is likely extremely rigid at this state, so subsidence would only be possibly in its ancient history, making it difficult to argue that recent lithospheric subsidence is likely to have occurred.

Thank you for these thoughtful comments. We agree that the original statement was too brief and did not explain clearly why volcanic loading is unlikely to be the primary driver of the observed deformation in the northern smooth plains.

In the revised manuscript, we now clarify that volcanic loading is indeed an important potential source of compressive stress on Mercury and has been invoked in previous studies to explain wrinkle-ridge formation, particularly in the Caloris basin (e.g., Kennedy et al., 2008; Watters et al., 2009; Cunje and Ghent, 2016). We therefore do not exclude volcanic loading as a contributing factor in the NSP. However, several observations suggest that it is unlikely to be the dominant mechanism there.

First, the loading-induced stress field is expected to depend strongly on the thickness and distribution of the plains materials. In the NSP, however, the observed heterogeneous spatial distribution of thrust-fault-related landforms (Crane and Klimczak, 2019; Cardinale et al., 2023) contrasts with the estimated distribution of lava flows, which does not show comparably strong spatial heterogeneity (Du et al., 2020). Second, Peterson et al. (2020) argued that the inferred depth of faulting in the NSP is greater than would be expected if volcanic loading were the primary cause, and also greater than the estimated thickness of the NSP lava sequence (Du et al., 2020), implying that larger-scale horizontal compressive stresses are required. Third, as you noted, Mercury's lithosphere likely became increasingly rigid through time. To reflect this point, we now cite Fleury et al. (2024), whose self-consistent three-dimensional thermal models indicate a substantial and rapid increase in elastic thickness in the NSP, making long-lived or geologically recent loading-related subsidence less likely.

We have therefore revised the text to clarify that volcanic loading may have contributed to deformation in the NSP, but that the available evidence does not support it as the primary driver of the regional contractional structures.

Stronger Contractional Strain in Mercury's Northern Smooth Plains

14). Line 43: the method descried by Byrne et al. (2014); Watters et al. (1998) and Watters (2021) is not a kinematic model. These approaches referenced here estimate global contraction based on the inferred cumulative strain of a population of observed thrust faults. An important note is that the relationship in all of these studies between dip angle and the amount of estimated global contraction is an artifact of the method in relating the estimated displacement to the vertical relief. Physically, lower dip angles do not mean a greater amount of global contraction. Loveless & Klimczak (2025), a study that resolved the discrepancy of the fault population studies mentioned in this line, showed that greater dips actually predict larger amounts of contraction.

Thank you for this important comment. We agree that our original wording was imprecise. In the revised manuscript, we no longer refer to these previous approaches as **kinematic models**. Instead, we describe them more accurately as methods that estimate global contraction from the inferred cumulative strain of mapped populations of contractional faults.

We have also revised the discussion of fault dip angle to avoid implying that lower dip angles physically correspond to greater global contraction. As you pointed out, the dependence of inferred contraction on dip angle in earlier fault-population approaches arises from the geometric assumptions used to relate fault relief to displacement, rather than from a direct physical relationship between dip angle and planetary contraction.

To reflect this more clearly, we now cite and discuss **Loveless & Klimczak (2025)**, whose analysis shows that alternative structural interpretations and strain partitioning can lead to different dip angle–contraction relationships. In light of this, we have moderated our wording throughout the manuscript and now treat the adopted dip angles in our study as **endmember**

assumptions used to test the sensitivity of the strain estimates, rather than as direct physical indicators of contraction magnitude.

Because our main conclusions focus on the **relative spatial pattern** of contractional strain and the **stability of the large-scale sampling results**, rather than on the absolute contraction value associated with any single dip-angle assumption, we consider this simplified framework adequate for the purposes of the present study. The manuscript has been revised accordingly.

15). Lines 50-51: how does a minimum length threshold "avoid the incompleteness" since applying a threshold is essentially constraining the data. This sentence needs to be revisited for clarity.

Thank you for this helpful comment. We agree that the original wording was unclear. Our intention was not to suggest that applying a minimum length threshold removes data incompleteness in a general sense, but rather that it reduces the likelihood of **incompletely capturing the topographic expression** of very short faults in our relief-extraction procedure.

In our method, each mapped fault is sampled using a series of parallel transects oriented perpendicular to the overall fault strike. The length of each transect is defined as $1.5 \times \min(L, 50)$ km, where L is the mapped fault length. Tests show that when faults are very short ($L < 10$ km), the transects are often too short to capture the full fault-related topography reliably. We therefore exclude faults shorter than 10 km to avoid introducing poorly constrained relief measurements into the strain analysis.

We also now note that Peterson et al. (2019) found that structures shorter than 10 km have only a minor effect on estimates of Mercury's global contraction, so this threshold is unlikely to affect our main conclusions. We have revised the manuscript to clarify this point.

16). Line 59: "our results indicate", What has been done? At this stage, the distinct methods that were used aren't being referenced, only what data sets were accessed, making it a bit confusing.

Thank you for this helpful comment. We agree that the original wording was too abrupt and did not provide enough methodological context before introducing the results. In the revised manuscript, we have added a brief description of the analytical workflow to the main text so that readers can understand what was done at this stage of the paper.

In brief, we estimate contractional strain by sampling Mercury's surface using a Fibonacci-sphere scheme. In this approach, evenly distributed sampling circles of different sizes are used to calculate the weighted cumulative fault length per unit area, analogous to a line-density calculation. We then examine how contractional strain varies with sampling size and use the stable large-scale behavior of these estimates to characterize the background contractional strain.

We have also clarified that the strain calculations are evaluated for assumed dip angles of 25, 30, and 35 degrees, and that the representative large-scale value is taken from the median of the strain distribution once the estimates stabilize.

To test robustness, we have also applied the same sampling framework to previously published global faults datasets, including Loveless & Klimczak (2025), Byrne et al. (2014), and Watters (2021), as shown below.

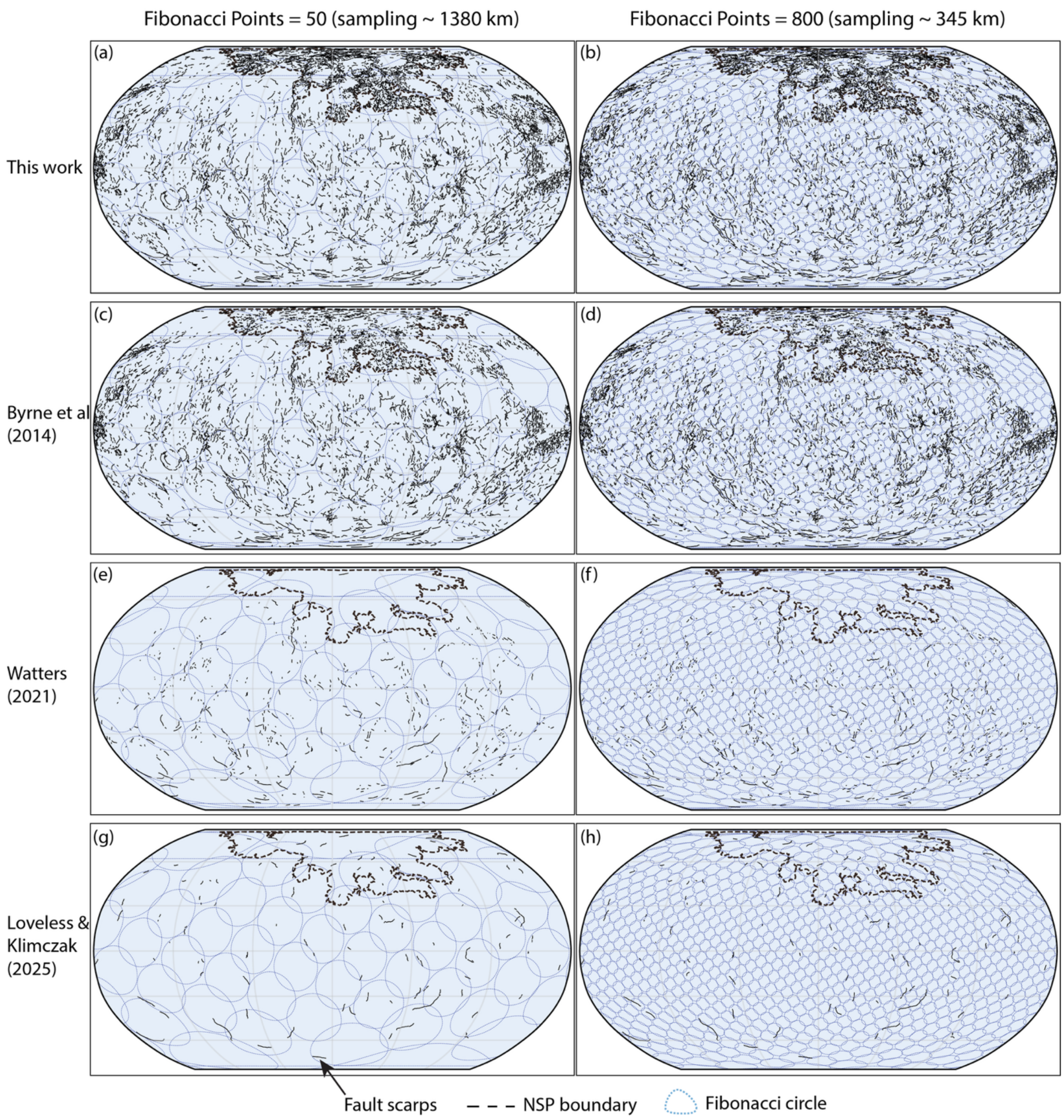

Extended Data Fig.3 *Tectonic maps of contractional structures across different fault datasets, and examples of Fibonacci circles corresponding to different sampling points.*

Despite a difference of nearly 4,000 mapped faults between our dataset and that of Byrne et al. (2014), the resulting global strain estimates are similar and fall within the range of earlier studies. By contrast, the datasets of Watters (2021) and Loveless & Klimczak (2025) do not

converge because their fault populations are too spare in number and spatial coverage (**Extended Data Fig.4a,b**). In these cases, once the sampling size exceeds the lengths of the vast majority of faults (e.g., the 85th percentiles of fault length; **Extended Data Fig.4c**), the resulting strain continuously decreases toward zero because the sampling area grows much faster than the cumulative weighted fault length per unit area. We have quantified this phenomenon using sampling coverage versus non-zero contractional strain shown in **Extended Data Fig.4d**. Taking Loveless & Klimczak (2025) as an example, when the sampling coverage exceeds 0.1 (corresponding to a sampling size of around 200 km; **Extended Data Fig.4c**), the contractional strain decreases rapidly and approaches zero. These findings indicate that for datasets with an insufficient number of faults and sparse spatial coverage, an uneven sampling scheme is a better option.

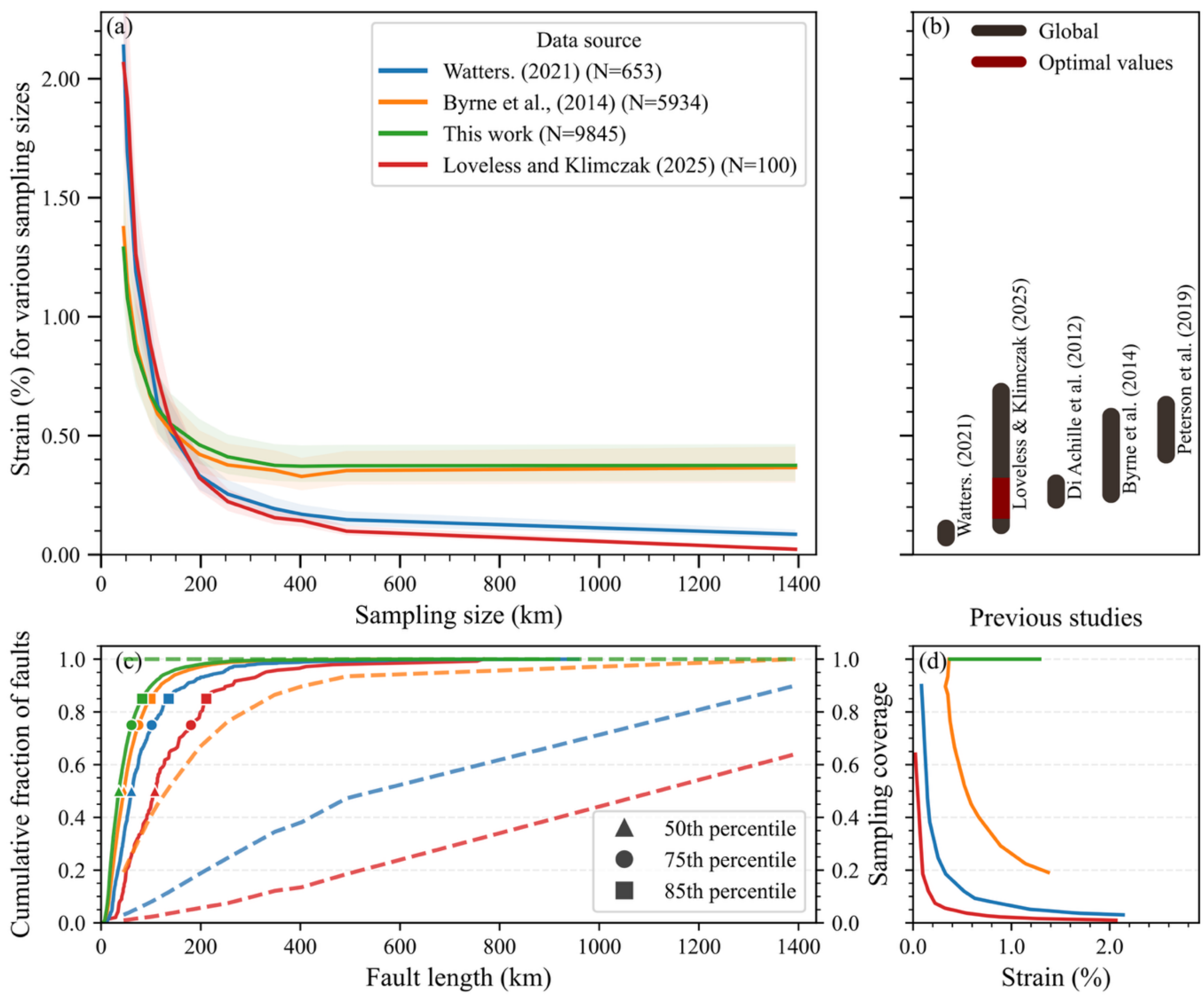

__Extended Data Fig.4__ (a) Mercury's global contraction strain across different fault datasets for various sampling sizes with a dip of 30°. (b) Previous results of Mercury's global contraction. (c) Fault length distributions with sampling coverage. Step curves show the fraction of faults shorter than a given length, with markers indicating the 50th, 75th, and 85th percentiles of fault length. Overlaid dashed-lines (right y-axis) give sampling coverage—the share of sampling circles with non-zero contractional strain—plotted against sampling size. (d) Sampling coverage

versus non-zero contractional strain estimates shown in panel (a). This panel illustrates how sampling completeness varies with the inferred strain across different sampling sizes.

We therefore have attempted to estimate the global contraction across different fault datasets under a simplified, uneven sampling scheme assuming a dip of 30° (**Extended Data Fig.5**). The key assumption of this scheme is that larger faults produce greater deformation, but this is regularized by their large sampling area. The presented results provide first-order estimates of strain attributable to individual faults. Although the average strain of Loveless & Klimczak (2025) remains lower than of the other datasets, it is broadly consistent to first order, and its large uncertainty likely due to its limited fault number and sparse spatial coverage.

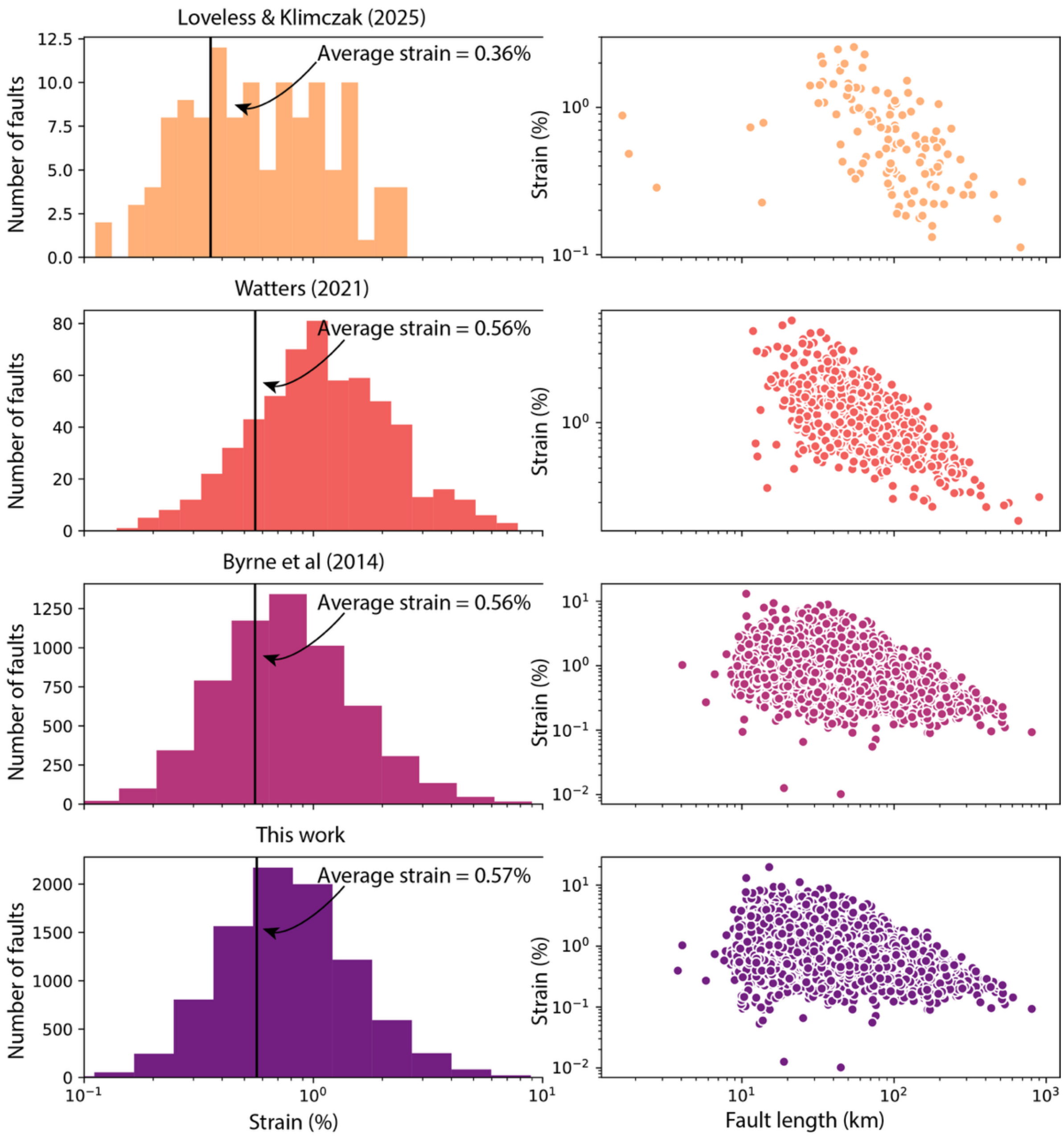

Extended Data Fig.5 *Comparison of contractional strain across different datasets assuming a dip of 30°. Left column: Histograms showing the distribution of strain on a logarithmic scale. The vertical line marks the area-weighted average strain for each dataset. Right column: Scatter of strain versus fault length on logarithmic axes, with each point representing and individual fault. Contractional strain is treated as a first-order estimate, calculated by* $\epsilon = \frac{HL}{sin(30°)A}$*, where* H *is the fault relief,* L *is the fault length.* A *is the faulted region for each fault, defined as* $A = \pi L^2$*. Accordingly, the area-weighted average contractional strain is obtained by* $\epsilon_a = \sum \epsilon_i A_i / \sum A_i$*.*

Overall, these findings suggest that the Fibonacci sampling scheme developed in this study requires fault datasets with sufficiently large sample sizes and broad spatial coverage. This also indicates that the fault-number-independent method adopted by Loveless & Klimczak (2025) has an advantage for small-sample datasets. The two approaches are not contradictory but complementary.

These revisions are now incorporated directly into the subsection *Stronger contractional strain in Mercury's northern smooth plains* and Extended Data.

17). Line 69: "when the sampling size is sufficiently large… …the median strain values reliably represent overall contraction…" the term reliably is used here, but there is no metric stated as to what strain estimates are reliable or not.

Thank you for this helpful comment. We agree that the term "reliably" was not defined clearly enough in the original manuscript. In the revised version, we now explain explicitly that "reliable strain estimates" refer to strain values obtained at sampling sizes for which the results become stable with increasing sampling size. In other words, once increasing the sampling size no longer produces substantial changes in the median strain estimate, we interpret the result as representative of the large-scale contractional signal rather than local variability.

We have revised the text accordingly so that this definition is given directly in the main text, rather than being left implicit.

18). Lines 72 to 78: the results of the Loveless et al. (2025) study are 1) of lobate scarp and wrinkle ridge endmember structures, 2) indeed, it was found that lobate scarp endmember structures had greater dips than that of wrinkle ridge endmembers, but this is just a correlation. Using simple trigonometry and just relating the relief and the fault dip to estimate the displacement, one would find that greater fault dips require less displacement. However, this is one relationship. In the study referenced, displacement and folding along the fault plane were partitioned differently. Additionally, on average, shortening landforms in the intercrater plains were found to have 1) greater amounts of displacement (i.e., almost double), and 2) greater amount strain partitioned to folding than those in the NSP. These arguments of this paper directly counter the arguments made here. Especially since, generally, larger shortening landforms were found to have faults that had greater dip.

Thank you for this important comment. We agree that the discussion in the original manuscript was overly simplified and did not adequately reflect the distinctions highlighted by Loveless et al. (2025), including the roles of structural endmembers and strain partitioning between fault slip and folding.

In the revised manuscript, we have clarified that the relationship between fault dip and inferred contraction is model dependent and should not be interpreted as a simple physical rule. As you noted, in approaches based only on geometric conversion between relief and displacement, steeper dips may imply smaller displacements, whereas Loveless et al. (2025) showed that when deformation partitioning and structural style are considered explicitly, different dip angle–contraction relationships can emerge. We now acknowledge this more clearly in the text.

We have therefore moderated our interpretation and no longer present dip angle as a direct physical predictor of contraction magnitude. Instead, the dip angles used in our study are treated as endmember assumptions for sensitivity testing within a simplified strain-estimation framework. Because our main conclusions concern the **relative spatial distribution** of contractional strain and the associated strain anomaly, rather than the absolute contraction value for any single structural model, we consider this approach appropriate for the scope of the present study.

The manuscript has been revised accordingly to reflect these points more clearly.

19). Why the distribution of fault lengths provides a comprehensive sample of the fault population is not discussed.

Thank you for this helpful comment. We agree that the original manuscript did not explain clearly enough why the fault-length distribution is informative for assessing whether the mapped dataset provides a sufficiently representative sample of the fault population.

In the revised manuscript, we now clarify that the fault-length distribution is used as a practical check on sampling completeness and representativeness within our strain-estimation framework. Because contractional strain in our approach is calculated from the cumulative weighted fault length within sampling areas, the estimated strain can be biased if the mapped dataset is dominated by only a limited subset of structures or does not capture the broader range of fault sizes. Showing the fault-length distribution therefore helps demonstrate that the dataset includes a broad spectrum of contractional landforms rather than being restricted to only the largest or most easily recognized features.

We also clarify that representativeness in our analysis is evaluated not from fault length alone, but from the combination of **fault-length distribution**, **broad spatial coverage**, and the **stability of strain estimates with increasing sampling size**. In this sense, the fault-length distribution provides one line of evidence that the sampled population is sufficiently comprehensive for large-scale strain analysis.

20). Almost nothing provided regarding the methodology to the point where the paper is hard to follow at this stage. A very general, short outline needs to be provided for some context.

Thank you for pointing this out. We agree that the original manuscript did not provide enough methodological context at this stage of the paper. In the revised version, we have added a brief general outline of the analytical approach to the main text so that readers can understand what was done before encountering the detailed results. This summary now explains, in concise terms, how contractional strain was estimated, how the sampling strategy was implemented, and how the resulting strain estimates were used to evaluate the models. The full technical details remain in the Methods and Extended Data.

Spatial Distribution of Deformation in the NSP

21). Line 95: "most regions of the NSP exhibit [a] negative strain anomaly". If it's contraction, shouldn't this strain be negative to begin with? What is the sign convention here? Meaning a negative anomaly would be more contraction, unless all of the contractional strain values have been converted to be positive which needs to be stated.

Thank you for this important comment. We agree that the sign convention for strain and strain anomaly was not stated clearly enough in the original manuscript. In our analysis, **contractional strain is treated as positive**, and the strain anomaly is calculated by subtracting the **mean contractional strain of the NSP** from the strain value at each sampling point.

Under this definition, a **positive strain anomaly** indicates contraction that is stronger than the NSP average, whereas a **negative strain anomaly** indicates contraction that is weaker than the NSP average. Thus, the statement that most regions of the NSP exhibit a negative strain anomaly means that contraction is present but generally weaker than the regional mean, not that the strain is extensional.

We have revised the manuscript accordingly to define the sign convention explicitly and to clarify the interpretation of the strain anomaly in both the text and the figure description. We have also rewritten this subsection to improve the presentation of the spatial distribution of deformation in the NSP.

Numerical Modeling of Mantle Upwelling Beneath The NSP

22). Lines 155-156: "Because mantle upwelling likely generated melt that formed the NSP lavas, the contractional deformation must have occurred after lava emplacement". Not all contractional deformation occurred after emplacement. Some faulting may have occurred prior and then reactivated into the smooth plains units. Especially since Loveless et al. (2025) found faults in the smooth plains to form with the bedrock.

Thank you for pointing this out. We agree that the original statement was too strong. In the revised manuscript, we no longer state that all contractional deformation must have occurred after emplacement of the NSP lavas. Instead, we now clarify that the temporal relationship between lava emplacement and contractional deformation remains uncertain, and that some faults may have initiated in the underlying basement and later been reactivated within the smooth plains, consistent with Loveless et al. (2025).

The revised text now reads (line 227-234):

Mantle upwelling likely generated the melts that formed the NSP lavas, but the relative timing of contractional deformation and lava emplacement remains uncertain. Because the mapped contractional structures likely record cumulative deformation and may include reactivated pre-existing structures (Loveless et al., 2025), the observed strain field is interpreted as a time-integrated measure of regional shortening rather than the product of a single instantaneous stress. We therefore integrate the horizontal strain under the assumption that most deformation postdates lava emplacement, marked by the onset of the topographic rise decline.

23). Figure 4: Compressional should be changed to contractional. What are the preferred models? How are they defined? This is not stated.

Thank you for pointing this out. We have corrected "compressional" to "contractional" in Fig.4.

We also agree that the term "preferred models" was not defined clearly enough in the original manuscript. In the revised version, we now state explicitly in the main text that the preferred models are those model configurations whose computed contractional strain is most consistent with the observations. In practice, these are the models that best reproduce the observed strain pattern, magnitude, and topography within the NSP.

We further clarify that a common characteristic of these preferred models is that the prescribed mass anomaly is close to, or equivalent to, the mass sheet derived from our mantle-dynamic loading model. This definition is now stated more clearly in the revised manuscript.

Controls on Surface Deformation

24). Lines 166-173: doesn't this method assume that the lithosphere is behaving viscously? And isn't the goal of this paper to relate mantle upwelling to the brittle deformation of the lithosphere? These assumptions should be stated (unless they are stated later in the methods), unless it is assumed that this upwelling occurred so early that much of the lithosphere is behaving in a viscous or ductile manner. Either way, these need to be stated.

Thank you for this important comment. We agree that the rheological assumptions of the model and their relationship to the observed brittle deformation should be stated more clearly.

In the present study, the lithosphere in the numerical model is treated as **viscous**. We adopt this simplification for three main reasons. First, the modelled mantle upwelling is assumed to have occurred early in the evolution of the NSP, broadly associated with the volcanic event that formed the plains at approximately 3.7 Ga. At that stage, the lithosphere may have been warmer and mechanically weaker than at present, so a viscous or ductile approximation is reasonable as a first-order representation. Second, the timing at which deformation in the NSP transitioned from ductile/viscous lithospheric response to dominantly brittle faulting remains uncertain. Third, explicitly modelling brittle failure would require a much more complex treatment of lithospheric structure, thermal state, material properties, and yield criteria, which is beyond the scope of the present parameter-survey study.

We also clarify in the revised manuscript that our goal is not to simulate individual brittle faults directly, but rather to test whether mantle upwelling can generate a first-order deformation pattern and accumulated strain consistent with the observed contractional structures. In this sense, the modelled viscous strain field is used as a large-scale proxy for the distribution and magnitude of lithospheric deformation that may later be expressed in brittle form. By comparing model-predicted accumulated strain with the observed areal strain, we assess whether mantle upwelling is a plausible driver of the deformation pattern in the NSP.

We have revised the manuscript accordingly to make these assumptions and limitations explicit in the main text and Methods. We also note in the discussion that future work should incorporate more realistic rheology, including brittle yielding and melt migration.

25). Lines 178 to 180: this should all be one sentence: "Using the inversion-derived areal density of the mass sheet together with the surface strain estimates, we apply pRa to constrain Mercury's lithospheric properties."

Thank you for pointing this out. We agree and have revised the sentence to read (line 311-312):

Combing the inversion-derived areal density of the mass sheet with the surface strain estimates, pRa can further constrain Mercury's lithospheric properties.

26). Figure 5: strain is contractional. Compression is a stress term. Furthermore, the equation should be introduced in the main text of the paper not here.

Thank you for pointing this out. We have replaced "compressional" with "contractional" in Fig.5 to use stress and strain terminology consistently. We also appreciate your suggestion regarding the placement of the equation. In the revised manuscript, we have clarified the physical meaning of pRa more explicitly in the main text, while retaining the formal definition in Fig.5 for ease of reference. We hope this presentation improves readability without interrupting the flow of the discussion.

Tectonic Evolution of the NSP and its Implications to Mercury's interior

27). Line 193: deformation is already plural in this sense. Remove the "s" from the end of the word.

Thank you for pointing out this error. We have revised the manuscript accordingly.

28). Lines 189-198: this is an interesting finding of the paper. If extension preceded contraction, then much of these thrusts in the NSP can be re-activated normal faults (inverted into thrusts). A topic that should be touched on.

Thank you for your positive comment. We have revised the manuscript accordingly.

29). Lines 199-203: I am hesitant to agree with persistent mantle convection. There is a rheological assumption of the mantle convection model shown here, assuming that the mantle behaves viscously. However, if Mercury is sufficiently cold, which it is as it has undergone

global contraction, then its mantle would be too rigid for further convection. However, a remnant upwelling bulge perhaps could provide something structural which stresses can coalesce on (similar to the formation of ghost craters) and therefore, enhance the localized brittle deformation due to ongoing global contraction (just a thought, and I now see that mantle convection is unlikely is stated in line 204).

Thank you for this thoughtful comment. We agree that the phrase "persistent mantle convection" may overstate what can be inferred, particularly given the uncertainty in Mercury's present-day mantle rheology and thermal state. As you note, if Mercury's mantle has cooled sufficiently, sustained ongoing convection may be unlikely. In the revised manuscript, we therefore moderate our wording and clarify that our interpretation does not require present-day active mantle convection. Rather, the observed deformation pattern may reflect the effects of an earlier mantle upwelling event, or a waning/relict upwelling-related structure, that helped localize later deformation within the NSP. In this sense, a long-lived mantle-related topographic or lithospheric anomaly could have influenced where stresses were concentrated, even if vigorous convection had already diminished.

30). Figure 6: change compression in the right side of the figure into contraction. Also, in panel b), wrinkle ridges are shown to be only penetrating the smooth plains, however, they likely host faults that penetrate deeper than the smooth plains emplacement.

Thank you for these helpful comments. We have replaced **"compression"** with **"contraction"** in Fig.6 to maintain consistent stress–strain terminology throughout the manuscript.

We also agree that the original schematic may have implied an overly shallow geometry for the faults associated with wrinkle ridges. In the revised figure, we have adjusted the thrust faults so that they extend beneath the smooth-plains unit. At the same time, because the subsurface fault geometry is not well constrained, we now show the fault planes in cross section using **dashed lines** to indicate their schematic and interpretive nature.

In addition, as noted in our response to Reviewer #2 comment #18, we have revised Fig.6 more broadly to improve its clarity. Specifically, we now depict the lithosphere as thinner relative to the mantle, and we have cropped the figure to focus more closely on the lithosphere–mantle region rather than the core.

31). Line 225-226: upwelling is likely regional influencing alongside global contraction; however, I fail to see how this study shows that global contraction is being overridden. Yes, there may be localized deformation due to upwelling, but there is still contraction in the northern rise, implying that there may be a combination of events, but not necessarily one overriding the other. Global contraction is still likely the dominant stress source.

Thank you for this important comment. We agree that the term **"overridden"** was too strong and did not accurately reflect our interpretation. Our results do not indicate that regional mantle upwelling replaced global contraction as the dominant tectonic driver on Mercury. Rather, they

suggest that mantle upwelling and its subsequent evolution may have locally modified the tectonic expression of the broader global contractional stress field within the NSP.

We have therefore revised the text to make this point clearer.

Methods

Expression of contractional Strain

32). Line 243: the term "strain" should be used instead of "stress". And if it is "known to accommodate" a reference is needed here.

Thank you for pointing out this error and your suggestion. We have revised the manuscript accordingly.

33). Line 248: why are "geochemical" properties being used as a reference here? This should not have anything to do with "geochemical" properties.

You are right, and we have deleted the term 'geochemical'.

34). Line 258-262: there are many studies that look into the lithospheric thickness of Mercury (not just the one referenced here). Furthermore, Loveless et al. (2025) found depths of faulting well below 30 km (some up to 40-45 km).

Thank you for this helpful comment. We agree that the original text did not sufficiently reflect the broader range of published estimates for Mercury's lithospheric thickness and faulting depth. In the revised manuscript, we have added additional references, including Loveless et al. (2025), to better represent this range.

We also clarify that although published estimates of faulting depth vary substantially, our main conclusions are not sensitive to whether the brittle–ductile transition depth is taken to be 30 km or 40 km. We therefore use 30 km as a representative first-order value for the present analysis, while acknowledging the uncertainty and wider range of possible faulting depths discussed in the literature.

35). Line 265-266: "accordingly, we propose a quantitative classification scheme for contractional landforms: fault's length extending to or beyond this depth are categorized… " what exactly is being compared here? The length is the observed, map-view length of the fault (or surface break), while the depth of the fault is how deep the fault is penetrated. Why is it that the length is being compared to the depth of faulting? How is the "fault's length extending to or beyond this depth"?

Thank you for this important comment. We agree that the original wording was unclear and could incorrectly suggest that fault length and fault depth were being treated as physically equivalent dimensions. That was not our intention.

In the revised manuscript, we now explain more clearly that this comparison is used only as a **practical geometric criterion** for classifying faults relative to the thickness of the brittle layer. As faults grow, both length (L) and down-dip height (H) increase until H hits the base of the brittle layer. Beyond that point, further growth is primarily along strike, while down-dip height remains constant (i.e., $H \approx T$, where T is the full thickness of the brittle crust or the maximum faulting depth. This expression valid if we ignore corrections for dip for simplicity) (Scholz and Cowie, 1990; Schultz. 2003; Schultz et al., 2006; Polit et al., 2009). These through-going (restricted) faults accommodate disproportionately large fractions of total strain and seismic moment, featuring $L \gg H \approx T$. Therefore, in some literature (e.g., Schultz. 2003; Watters. 2021), faults with a length (L) greater than the maximum faulting depth (T) are defined as large faults.

We have rewritten this methodological section for clarify.

Extraction of thrust fault's relief

36). Lines 289-291: what does "the minimum value between the local fault length (L) and 50 km" mean? It is hard to picture what exactly is being described here. Shouldn't the transections be roughly perpendicular to the length of the fault? how is the local fault length defined?

Thank you for this helpful comment. We agree that the original wording was unclear. In the revised manuscript, we now state this more explicitly as:

"the length of each transect is defined as $1.5 \times min\ (L_c, 50) km,$*"*

where L_c is the locally measured fault length.

To clarify the method further, this quantity refers to the **length of each individual topographic transect** used to extract fault relief. In our approach, the transects are generated approximately **perpendicular to the local fault strike**, rather than simply perpendicular to the overall map-view length of the entire fault. We adopt this approach because fault traces are commonly curved or segmented, and relief extraction is more robust when profiles are referenced to the **local strike geometry**.

Specifically, we assume that the local deformation is best characterized in a direction normal to the local fault strike, which we define using the local end-to-end azimuth of the mapped segment. Based on this geometry, we generate a series of equally spaced, parallel topographic transects for each fault. The local fault length L_c is then calculated in a locally projected coordinate system centered on the fault centroid. We have clarified this procedure in the revised Methods section, and the full workflow is also summarized in **Extended Data Fig.1**.

Calculation of areal strain

37). Lines 318 – 322: is there an appropriate reference regarding why faults should be weighted towards the strain? Is this really representative of areal strain?

Thank you for this important comment. We agree that the rationale for the weighting scheme and its connection to areal strain needed to be explained more clearly.

In previous studies, contractional strain is commonly estimated by summing fault-related shortening and normalizing by the total area of the terrane (e.g., Peterson et al., 2019). While this provides a bulk strain estimate, it does not capture how strain varies spatially. In our study, we extend this framework by discretizing Mercury's surface using a Fibonacci-sphere scheme and calculating strain within sampling circles of different sizes. This allows us to evaluate the spatial distribution of contractional strain while minimizing latitudinal sampling bias on the sphere (González, 2010).

Within each sampling circle, the strain estimate is computed from the cumulative fault contribution normalized by the sampled area. In this sense, the weighting does not introduce a different physical definition of strain; rather, it is a spatial reformulation of the conventional fault-based strain calculation so that the contribution of mapped structures can be evaluated locally. This is analogous to weighted line-density methods commonly used in GIS-based spatial analysis (e.g., Silverman, 2018). We have revised the manuscript to clarify this point and added the relevant references.

38). Lines 323-326: what is the defining point between a small and large structure? This is an important definition.

Thank you for this helpful comment. We agree that this definition should be stated more clearly. In the manuscript, we follow the fault-scale criterion of Watters (2021) and other previous studies (e.g., Schultz. 2003), in which faults are classified as large when their mapped length is greater than or equal to the maximum depth of faulting. We now make this dividing criterion more explicit in the subsection Expression of contractional strain and cross-reference the related methodological explanation.

39). Line 351: the "w" in we need to be capitalized.

Thank you for pointing out this error. We have revised the manuscript accordingly.

Extended Data figures

40). Extended Data figure 2: there appears to be no difference between the three panels.

Thank you for pointing this out. We found that an incorrect dataset had been used in plotting this figure. We have now replotted Extended Data Fig.2 using the correct data. Although the differences among dip angles and sampling sizes are modest, the revised figure now correctly reflects those variations and does not affect our conclusions.

41). Extended Data figure 4: if this is stress, then the term extensional should be changed to

Tensile.

Thank you for pointing this out. You are correct that Sxx is a stress term. We have therefore replaced "extensional" with "tensile" in the figure to maintain consistent stress–strain terminology.

References:

Cunje, A. B., & Ghent, R. R. (2016). Caloris basin, Mercury: History of deformation from an analysis of tectonic landforms. Icarus, 268, 131-144.

Crane, K. T., & Klimczak, C. (2019). Tectonic patterns of shortening landforms in Mercury's northern smooth plains. Icarus, 317, 66-80.

Cardinale, M., Vaz, D. A., D'Incecco, P., Mari, N., Filiberto, J., Eggers, G. L., & Di Achille, G. (2023). Morphostructural mapping of Borealis Planitia, Mercury. Journal of Maps, 19(1), 2223637.

Du, J., Wieczorek, M. A., & Fa, W. (2020). Thickness of lava flows within the northern smooth plains on Mercury as estimated by partially buried craters. Geophysical Research Letters, 47(20), e2020GL090578.

Fleury, A., Plesa, A. C., Tosi, N., Walterová, M., & Breuer, D. (2024). Variations of heat flux and elastic thickness of Mercury from 3-D thermal evolution modeling. Geophysical Research Letters, 51(21), e2024GL110622.

González, Álvaro. "Measurement of areas on a sphere using Fibonacci and latitude–longitude lattices." Mathematical geosciences 42.1 (2010): 49-64.

Kennedy, P. J., Freed, A. M., & Solomon, S. C. (2008). Mechanisms of faulting in and around Caloris basin, Mercury. Journal of Geophysical Research: Planets, 113(E8).

Peterson, G. A., Johnson, C. L., Byrne, P. K., & Phillips, R. J. (2019). Distribution of areal strain on Mercury: Insights into the interaction of volcanism and global contraction. Geophysical Research Letters, 46(2), 608-615.

Peterson, G. A., Johnson, C. L., Byrne, P. K., & Phillips, R. J. (2020). Fault structure and origin of compressional tectonic features within the smooth plains on Mercury. Journal of Geophysical Research: Planets, 125(7), e2019JE006183.

Polit, A. T., Schultz, R. A., & Soliva, R. (2009). Geometry, displacement–length scaling, and extensional strain of normal faults on Mars with inferences on mechanical stratigraphy of the Martian crust. Journal of Structural Geology, 31(7), 662-673.

Silverman, B. W. (2018). Density estimation for statistics and data analysis. Routledge.

Schultz, R. A. (2003). A method to relate initial elastic stress to fault population strains. Geophysical Research Letters, 30(11).

Schultz, R. A., Okubo, C. H., & Wilkins, S. J. (2006). Displacement-length scaling relations for faults on the terrestrial planets. Journal of Structural Geology, 28(12), 2182-2193.

Scholz, C. H., & Cowie, P. A. (1990). Determination of total strain from faulting using slip measurements. Nature, 346(6287), 837-839.

Watters, T. R., Murchie, S. L., Robinson, M. S., Solomon, S. C., Denevi, B. W., André, S. L., & Head, J. W. (2009). Emplacement and tectonic deformation of smooth plains in the Caloris basin, Mercury. Earth and Planetary Science Letters, 285(3-4), 309-319.

Watters, T. R., Selvans, M. M., Banks, M. E., Hauck, S. A., Becker, K. J., & Robinson, M. S. (2015). Distribution of large‐scale contractional tectonic landforms on Mercury: Implications for the origin of global stresses. Geophysical Research Letters, 42(10), 3755-3763.

Watters, T. R. (2021). A case for limited global contraction of Mercury. Communications Earth & Environment, 2(1), 9.

Reviewer #2 (Dr. Jupiter (Cheng) Hansen):

This manuscript is very interesting. It presents a novel, paradigm-changing hypothesis for the tectonic evolution of NSP, a new perspective on Mercury. The authors employ a powerful combination of geophysical and tectonic modeling and observations.

I have extensive line-by-line comments below but believe the fundamental conclusions that are presented here. I think most of my comments can be addressed with minor edits to the manuscript (e.g., changes to wording, editing existing figures, paragraph organization, etc.)

We sincerely thank you for your positive, thoughtful, and constructive review. We are encouraged by your assessment that the manuscript presents a novel perspective on the tectonic evolution of Mercury's northern smooth plains (NSP), and we appreciate your many detailed suggestions. We have revised the manuscript extensively in response to your comments. Below, we address each point in turn.

I have two major comments:

1). Inclusion of sample-size independent contraction estimates: The manuscript should incorporate the recently published sample-size independent contraction estimates by Loveless and Klimczak (2025), particularly in Figure 1c and the related discussion. Explicitly clarifying how these results integrate with the authors' methodology will strengthen the interpretation of NSP strain anomalies. See the line-by-line comment for lines 14 – 17.

Thank you for this important suggestion. We agree, and we have now incorporated the results of Loveless & Klimczak (2025) into both the revised manuscript and Fig.1c. We also expand the related discussion to clarify how their fault-number-independent estimates compare with our sampling-based strain framework.

In the revised manuscript, we now emphasize that the approach of Loveless & Klimczak (2025) and our approach are complementary rather than directly interchangeable. Their study provides robust global contraction estimates using a fault-number-independent framework based on representative contractional structures, whereas our analysis evaluates how contractional strain varies with spatial sampling scale using broadly distributed fault maps. Reassuringly, our strain estimates remain within the range reported by Loveless & Klimczak (2025).

This point is discussed in more detail in our responses to comments #5, #8, and #17.

2). Visualization of strain anomalies and structural orientations: The spatial distribution of strain anomalies and the orientations of thrust faults and grabens should be visualized more clearly. Interpolated strain maps and structural orientations (e.g., structural map and even better with rose diagrams) relative to the NR would allow readers to evaluate whether the observed deformation patterns are consistent with the predicted tectonic model rather than random or controlled by other factors, such as large impacts. See the line-by-line comment for Figure 2.

Thank you for this excellent suggestion. In response, we have substantially revised Fig.2 and the related discussion in the manuscript to improve the visualization of the strain-anomaly pattern and its relationship to mapped tectonic structures.

The revised figure now includes:

a) the strain-anomaly map,
b) the corresponding radial average anomaly for a 50-km bin size,
c) fault orientation pattern versus strain anomaly, and
d) dominant fault strikes relative to the radial direction from the Northern Rise (NR).

We also now clarify explicitly that the strain anomaly is defined relative to the mean contractional strain of the NSP, such that:

- positive anomaly values indicate stronger-than-average contraction, and
- negative anomaly values indicate weaker-than-average contraction.

Therefore, negative anomalies do not imply extensional strain. The revised figure and discussion now make the concentric deformation pattern easier to evaluate, allow clearer comparison with the distribution of contractional and extensional structures, and provide stronger support for the interpretation that mantle upwelling contributed significantly to deformation within the NSP.

This is discussed further in our responses to comments #9 and #10.

Overall, it is a well-written and informative contribution to Mercury studies and deserves to be published once a few aspects are expanded upon. Thus, my recommendation is that the paper should be acceptable after pending suitable major-to-moderate revision.

We are grateful for your overall positive assessment of the manuscript and for your recommendation that it is suitable for publication after revision. Below we respond to your line-by-line comments.

Line-by-line comments:

3). Line 2: "offers as a unique window".

Thank you for catching this. We have corrected the text to read "offers a unique window."

4). Line 7: It should be added as "tectonic" forces to be more specific.

We agree with you and have revised the manuscript accordingly.

5). Lines 14 – 17: I totally agree with this. It is worth noting that current work estimates the 2 to 3.5 km of radial contraction using a sample-size independent model, resulting in a lower upper bound strain than the previous sample-size dependent models (i.e., 7.6 km by Watters 2021 and 8.4 km by Byrne et al. 2014).

Loveless, S. R., & Klimczak, C. (2025). Several kilometers of global contraction on Mercury: A sample-size independent assessment of fault strain. AGU Advances, 6(4), e2025AV001715.

Thank you for this helpful suggestion. We have added the results of Loveless & Klimczak (2025) and expanded the discussion accordingly.

The revised text now reads (line 14-23):

There may be two possibilities: (1) the contractional strain of the NSP has been overestimated because commonly used computational method extrapolates strain to the study area from limited populations of representative structures (Watters et al., 1998; Di Achille et al., 2012; Peterson et al., 2019; Watters, 2021), a concern supported by a recent fault-number-independent analysis that yields lower estimates of Mercury's global contraction (Loveless & Klimczak, 2025) than earlier assessments (Byrne et al., 2014; Peterson et al., 2019); or (2) mechanisms other than global contraction have also contributed to the contractional deformation in this region. If the former is true, it remains difficult to explain the persistence of contractional activity into recent geological times, as evidenced by the identified young grabens (~300 Myr) (Man et al., 2023) and small-scale scarps (~50 Myr) (Watters et al., 2016) in the NSP. If the latter stands, one must then ask, what might these mechanisms be?

We have also moderated the wording so that the discussion better reflects the model dependence of dip-angle-based strain estimates.

6). Line 22 – 27: It is a very long sentence with a lot of information. I suggest rewriting it into two like: Previous studies have proposed volcanic loading as an additional driving mechanism [5, 8]. However, the pronounced spatial clustering of contractional landforms in the NSP [7, 16] is inconsistent with the expected uniformity of deformation from volcanic loading, as is the absence of significant spatial variations in lava flow thickness [17], which would be necessary to generate localized loading and associated tectonic deformation. Also, I suggest starting as a new paragraph with "Previous studies…"

Thank you for this helpful suggestion. We agree that this part of the introduction was overly long and difficult to follow. We have revised the manuscript accordingly and improved the paragraph structure.

7). Lines 59 – 78: The logic is solid, but the paragraph organization is somewhat unclear due to the mixing of multiple ideas in a single block. I suggest keeping the first paragraph from "Our results indicate that the contractional …" through "across different sampling sizes." Then starting a new paragraph with "This interpretation assumes that scarps in the NSP and other terranes have the same average dip angle."

Thank you for this suggestion. We agree and have reorganized this section to separate the methodological explanation from the interpretation more clearly.

8). Figure 1: This is a strong and well-designed figure, but a few aspects could be improved.

(1) Because the figure is intended to demonstrate the dependence of estimated contractional strain on sampling size, it would be helpful to include the sample sizes used in previous studies. In panel (c), strain estimates from earlier work are ordered from small to large, but their corresponding sample sizes are not shown. For example, Byrne et al. (2014) analyzed 5,934 shortening landforms, whereas Watters et al. (2021) considered 653. Displaying these values, or another quantitative measure of sampling size, either on the bars or along the x-axis would provide important context for interpreting the differences among studies.

(2) Several previous studies focus on localized regions rather than the entire "other terrane." For instance, Di Achille et al. (2012) covers approximately 21% of the planetary surface. Outlining the study areas of each prior work in panel (a), while retaining the full "other terrane" in blue, would help clarify whether differences in estimated strain also reflect study area selection. This would also avoid the potential misinterpretation that all published strain estimates represent the entire "other terrane."

(3) As noted in my comments for line 14-17, the sample-size independent result from Loveless & Klimczak (2025) should be included in the figure and related main text.

(4) It is probably me being nitpicky but the max in "Dmax" looks like the same size as D in the block diagram in panel (b) when it should be smaller since it is in subscript.

Thank you for these very helpful suggestions. We have redrawn **Fig.1** accordingly.

Specifically:

- We now additionally plot global strain as a function of sampling size and recalculate global strain using the same dataset as **Byrne et al. (2014)** to facilitate direct comparison.
- In panel **(c)**, we add the **maximum, minimum, and optimal** strain values reported by **Loveless & Klimczak (2025)**.

- We also annotate the number of mapped faults used in earlier studies, to provide clearer sampling context.
- Because several previous studies estimated **global contraction** even when using geographically restricted study areas, we relabeled panel **(c)** to refer to **global strain** rather than "other terrane strain."
- Finally, we corrected the formatting of the subscript in D_{max}.

The revised figure and figure caption now reads:

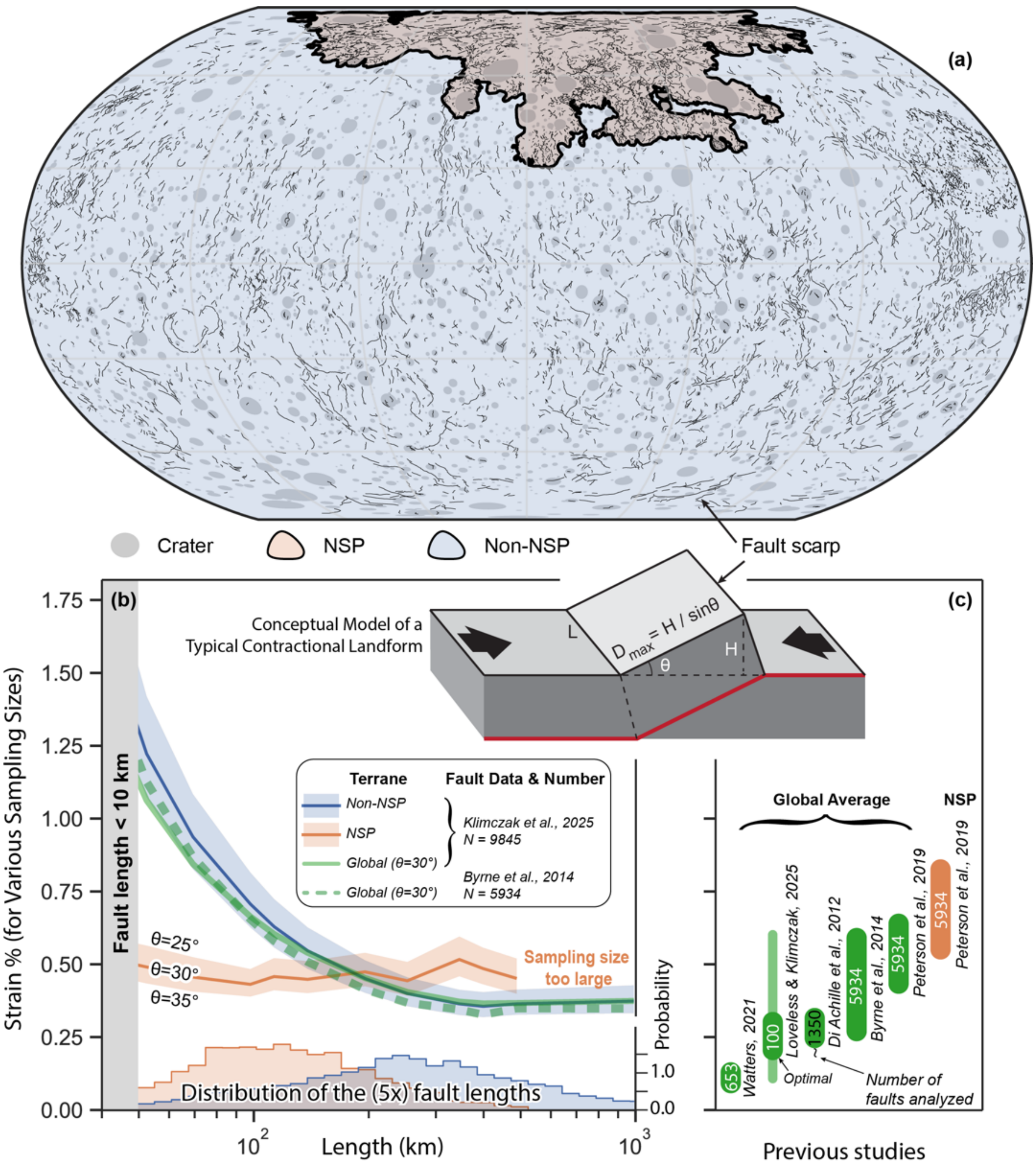

Fig.1 *(a) Global distribution of contractional landforms and craters across Mercury. (b) Contractional strain for various sampling sizes, which converges toward a stable value representative of the overall contraction of each terrane. The distribution of* $5\times$*fault lengths suggests that the largest sampling windows adequately capture the mapped fault population. The inset depicts a conceptual model of a typical contractional landform, where* H, θ, *and* L *are the fault relief, dip angle, and length, respectively, and* D_{max} *is the maximum displacement. (c) Comparison of Mercury's contractional strain estimates from previous studies.*

9). Figure 2: The statement in line 102 that "the strain anomaly demonstrates a concentric distribution of strain, with negative anomalies at the central area (NR) and positive anomalies outward" is plausible, but this pattern is difficult to evaluate from Figure 2 as currently presented. An interpolated strain map would help more clearly visualize this spatial distribution. Because the concentric pattern is central to the manuscript's interpretation, explicitly illustrating it would substantially strengthen the argument.

Thank you for this helpful suggestion. We agree that the original version of Fig.2 did not show the spatial pattern clearly enough. We have therefore redrawn the figure to better illustrate the distribution of strain anomaly across the NSP.

We now clarify in both the main text and figure description that the anomaly is defined relative to the mean contractional strain of the NSP, so that **positive** values indicate **stronger**-than-**average** contraction and **negative** values indicate **weaker**-than-**average** contraction. Under this definition, the inferred concentric pattern reflects spatial variation in contractional intensity within the NSP, rather than a transition from contraction to extension.

10). Line 99 – 101: Related to the above point, impact-related tectonism may be a major reason the strain pattern appears less clear. To test this interpretation, it would be useful to plot thrust fault scarps and grabens across the NSP using the mapping data already shown in Figure 1a, ideally overlain on the interpolated strain map suggested above. This would allow readers to assess whether strain anomalies are indeed disrupted near large impact structures and to evaluate the relationship between shortening and extension versus positive and negative anomalies.

More importantly, such an approach could also illustrate the orientations of these structures relative to the central NR area. As discussed later in the manuscript (particularly in Figure 6), the waned mantle upwelling hypothesis predicts not only higher deformation concentrated in the NSP but also preferred deformation orientations with respect to NP. Specifically, extension (Figure 6a) and compression (Figure 6b) are expected to radiate outward from NP, producing grabens and thrust faults concentric around the center. I would even suggest plotting rose diagrams projected around the center of the NSP, if possible, to directly visualize these orientation patterns. Providing this visualization and analysis is essential to demonstrate that the observed deformation is not simply controlled by variations in crustal properties or global contraction, which would more likely produce random orientations, and to ensure that the spatial distribution within the NSP is not biased by large impacts near its margins.

To accommodate the additional visualization, it may be preferable to show only the 50 km bin-size panel in the main figure and place the 25 km and 100 km panels in the Extended Data, particularly since the differences mainly reflect local variability, as noted in the manuscript. The final choice of presentation, of course, remains with the authors.

Thank you for these helpful suggestions. We agree that the role of impact-related tectonism, as well as the spatial relationship among contractional structures, extensional structures, and strain anomaly, should be illustrated more clearly. In response, we have substantially revised **Fig.2** and the corresponding discussion in the manuscript.

In the revised figure, we retain only **(a)** the strain-anomaly map and **(b)** the corresponding radial average anomaly for different assumed fault dip angles using a 50-km bin size. The other panels from the original figure have been removed. In their place, we add **(c)** fault orientation pattern versus strain anomaly and **(d)** dominant fault strikes relative to the radial direction centered on the Northern Rise (NR).

We now clarify explicitly that the **strain anomaly is defined relative to the mean contractional strain of the NSP**, such that **positive anomaly values indicate stronger-than-average contraction**, whereas **negative anomaly values indicate weaker-than-average contraction**. Thus, negative anomalies do not imply extensional strain. With this definition, most of the central NSP, corresponding to the NR, is characterized by negative strain anomalies and is largely devoid of large impact craters, whereas positive anomalies are concentrated mainly in the surrounding regions. This pattern is further supported by the density-kernel contours, which shows a concentration of negative anomaly values over the NR.

When contractional and extensional structures are overlaid on the revised strain-anomaly map, extensional structures are concentrated mainly within the NR and nearby areas, whereas contractional structures are more commonly associated with regions of stronger contractional deformation. This relationship is consistent with the interpretation that some extensional structures may represent secondary features related to the continued deformation of pre-existing contractional landforms (Man et al., 2023). The revised figure also makes it easier to assess whether large impacts locally disturb the broader deformation pattern.

Following your suggestion, we also examine the orientation of thrust-faults relative to the radial direction from the center of the NR. Near the NR, the dominant fault strikes are approximately perpendicular to the radial direction. Farther from the NR, except in regions influenced by large impacts and in the area north of the NSP that may record a distinct tectonic overprint, the dominant strikes tend to become subparallel to the radial direction. This systematic orientation pattern is consistent with a concentric compressive stress field centered on the NR, rather than deformation controlled solely by random crustal heterogeneity or background global contraction.

We also add a new three-dimensional elastic model to test whether localized loading at the Northern Rise can explain the observed radial fault orientations (We paste it below; **Extended Data Fig.9**). The new analysis shows that outside the NR the maximum horizontal compressive stress if concentric about the load center, implying radial contractional faults because reverse-fault strike is expected to be perpendicular to the maximum compression. We further show that

this first-order pattern is controlled mainly by the geometry of the NR load and is only weakly sensitive to the position of the imposed outer constraint. We have added this discussion to the end of Section 6 and the model description to the **Methods (Three-dimensional elastic model)** and **Extended Data Table.3**, and we present the results in **Extended Data Fig.9**.

Taken together, these revisions improve the visualization of the deformation pattern within the NSP and provide additional support for the interpretation that mantle upwelling contributed significantly to deformation in this region.

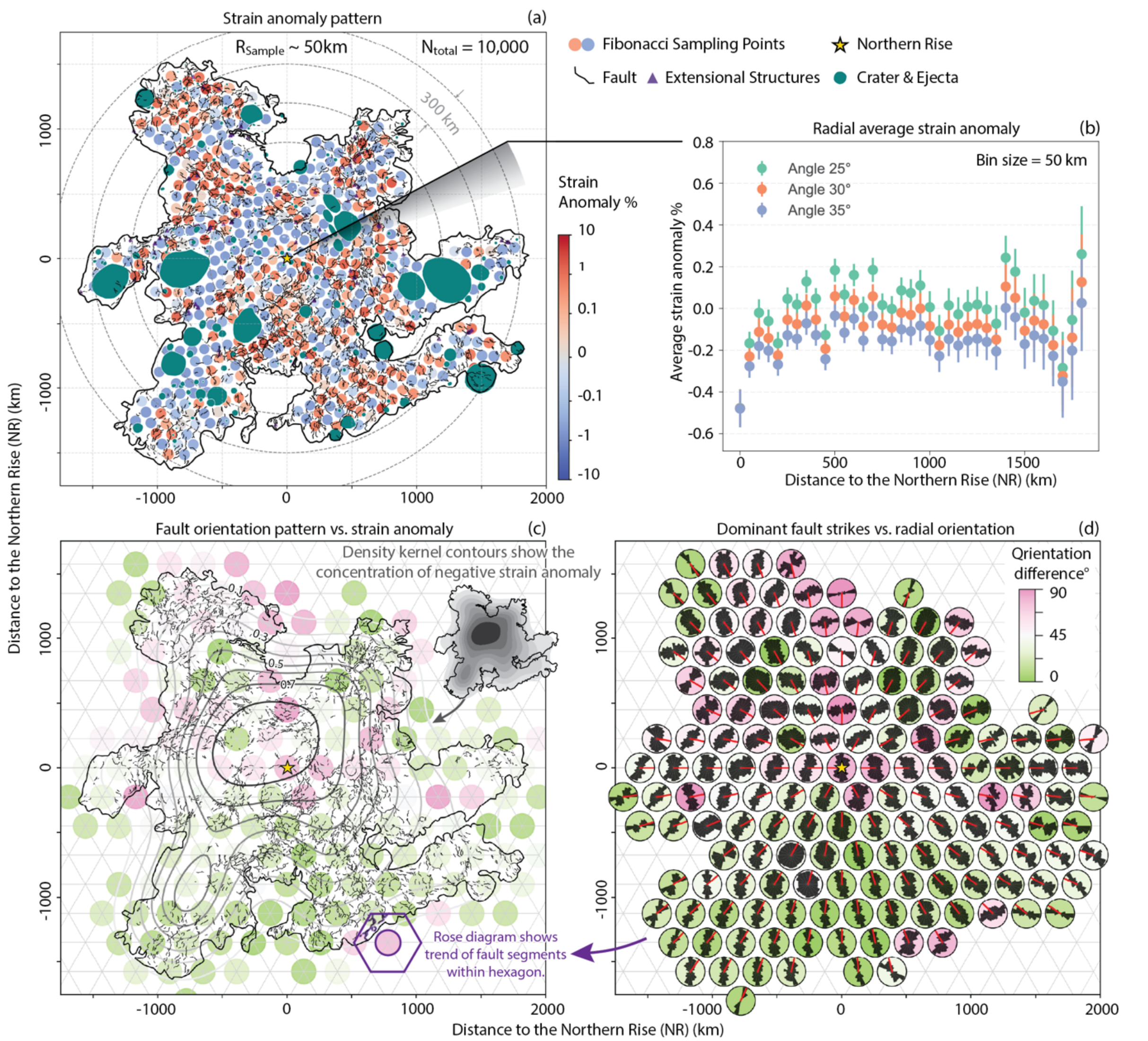

Fig.2 (a) Strain anomaly map of the NSP for Fibonacci sampling points of 10,000. (b) Corresponding radial average strain anomalies at different fault's dip angle with a bin size of 50 km. (c) Strain anomaly versus Fault orientation pattern, and (d) Dominant fault strikes relative to the radial direction from the center of the Northern Rise. A positive strain anomaly indicates stronger-than-average contraction. Maps are reprojected into a local coordinate reference system centered on the Northern Rise. Extensional structures are indicated by equilateral

triangles (Men et al., 2023); crater and ejecta distributions follow (Crane and Klimczak, 2019; Hall et al., 2021).

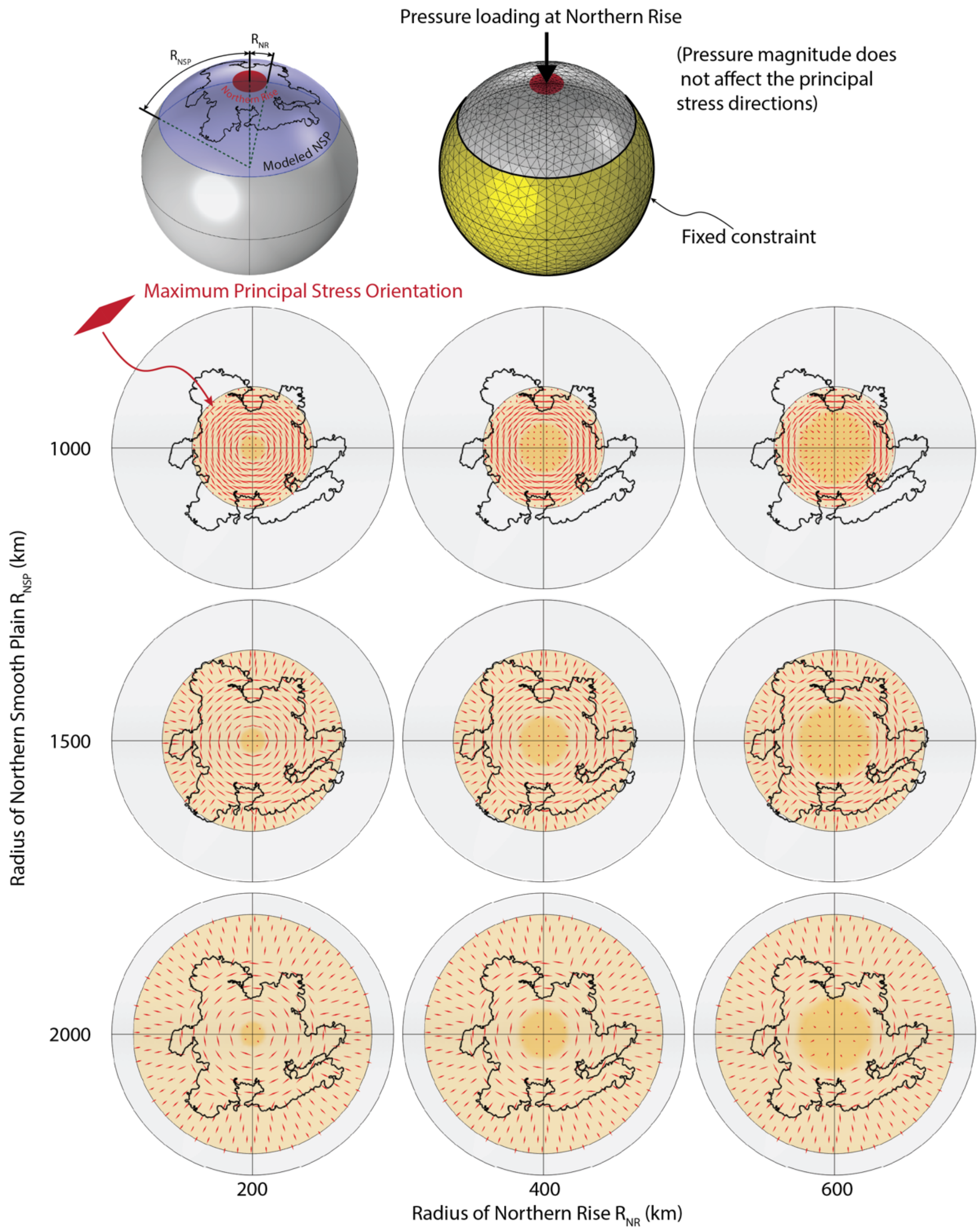

Extended Data Fig.9. Predicted maximum principal stress orientations under pressure loading at the Northern Rise.

11). Caption of Figure 3: "Results from the mantle dynamic loading model in orthographic projection centered on the Northern Rise (NR) (30◦E, 70◦N), where (c) Mantle mass sheet, (d) Predicted dynamic topography, (e) Predicted dynamic gravity, and (f) Predicted dynamic

pressure." | This sentence is ungrammatical, as "where" cannot be used to introduce a list of panels without a verb. Replacing "where" with "showing" would resolve this issue.

We agree with you and have revised the caption of Figure 3. The caption now reads:

(a) Topography and (b) free-air gravity anomalies at spherical harmonic degrees of 15. Results from the mantle dynamic loading model in orthographic projection centered on the Northern Rise (NR) (30E, 70N), showing (c) Mantle mass sheet, (d) Predicted dynamic topography, (e) Predicted dynamic gravity, and (f) Predicted dynamic pressure. The black solid and dashed line respectively frame the boundary of the Northern smooth plains the NR.

12). Lines 141 – 142: "Therefore, we largely expand the parameters space configuring numerical simulations." | The phasing in this sentence is unclear and unidiomatic. Perhaps rewrite it as: "Therefore, we consider a wide range of parameters used to configure the numerical simulations."

Thank you for this helpful suggestion. We have revised the sentence accordingly.

13). Lines 149 – 150: Revise this phase as "followed by gradual subsidence after several hundred million years"

Thank you for this helpful suggestion. We have revised the sentence accordingly.

14). Figure 4: The figure caption is somewhat confusing, as panel (b) includes a thick blue line labeled "Model" as well as a blue-to-green color scale representing maximum extensional strain. I assume these two elements refer to the same model. If so, I suggest removing the thick blue line from the legend and instead labeling the color scale as the model output. If I have misunderstood and the thick blue line represents a different model, then using a color distinct from blue would help avoid confusion. In either case, additional clarification in the figure caption would be helpful.

Thank you for this helpful suggestion. We have redrawn Fig.4 and clarified the caption and legend to avoid ambiguity.

15). Line 169: The "–" is a bit awkward here. I think it is better to use commas here and same as in line 170, such as "…parameter, the pseudo-Rayleigh number (pRa; Fig.5), which…"

Thank you. We have revised the manuscript accordingly.

16). Line 178: The sentence reads awkwardly and appears to be a sentence fragment. Please rewrite for clarity.

Thank you for pointing this out. We have revised the text to read (line 308-312):

This constrained pRa establishes a critical connection between the geophysical inversion results (Fig.3) and the surface deformation recorded in geological structures, in a manner consistent

with conservation of the mantle mass anomaly. Combining the inversion-derived areal density of the mass sheet with surface strain estimates, pRa can further constrain Mercury's lithospheric properties.

17). Section 6: I think this section (and probably section 5 as well) contains substantial methodological detail, much of which is described elsewhere in the Materials and Methods section. I suggest referring to Materials and Methods section when suitable in these sections.

Thank you for this helpful suggestion. We agree that some parts of Sections 5 and 6 included more methodological detail than necessary in the Results. In the revised manuscript, we have streamlined these sections and added clearer references to the Materials and Methods where appropriate.

18). Figure 6: It is a very nice schematic illustration. However, the relative thickness of the lithosphere and mantle appear similar in some locations, which makes the figure look slightly odd. I suggest making the lithosphere thinner than the mantle, especially given the relatively thin lithosphere assumed in this study. The figure could also be more tightly zoomed into the lithosphere–mantle region, as the core occupies nearly half of the illustration but is not the focus on this study. Also, seeing the predicted tectonic orientations in this illustration makes me feel strongly that a map showing the observed structure projected in NSP is needed on top of the spatial distribution of strain anomalies in Figure 2. See my comments for lines 99-101.

Thank you for this helpful suggestion. We have revised Fig.6 so that the lithosphere is shown thinner relative to the mantle and the figure is more tightly focused on the lithosphere–mantle region, with less emphasis on the core. We also incorporated your suggestion to investigate fault orientations relative to the deformation pattern, and this analysis is now included in the revised Fig.2 and related discussion.

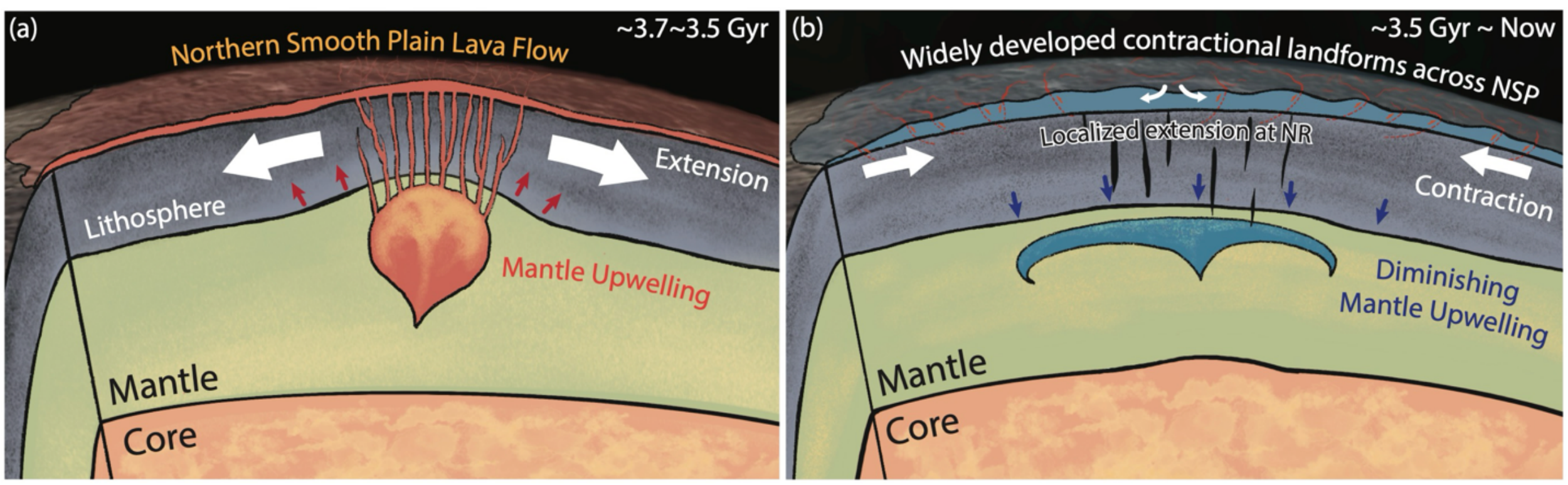

19). Line 212: I suggest replacing "–" with ";"

Thank you. We agree with you and have revised the manuscript accordingly.

20). Line 215: I suggest deleting "Then," and starting the sentence with "A key question is whether…", which would strengthen the statement.

Thank you for this suggestion. We have revised the wording accordingly. Please also see our response to comment #21.

21). Line 217 – 221: What could explain why the proposed large impact produces results so different from those of the Caloris basin? One or two sentences of justification would be useful here.

Thank you for this important question. We agree that the original manuscript did not explain this distinction clearly enough.

A reasonable explanation is that the NSP is interpreted as an indirect product of a large-impact event, rather than the direct tectonic and thermal expression of the impact basin itself. Numerical simulations suggest that impacts comparable in scale to those that formed the Caloris and Rembrandt basins could have introduced large thermal anomalies into Mercury's lower mantle far from the impact site, thereby promoting mantle upwelling and volcanism (Roberts and Barnouin, 2012; Padovan et al., 2017). This view was later adopted by Wang et al. (2021), who proposed that the NSP formed during a global volcanic episode likely triggered by large impacts.

Accordingly, the tectonic evolution of the NSP would differ substantially from that of an impact basin such as Caloris, because the former reflects a more indirect mantle-mediated response rather than direct impact modification.

However, in our revised manuscript, we compact some technical details and therefore remove the extensive lines regarding the impact origins. The revised words (line 334-338) now read:

This interpretation implies that the NSP is not simply a passive site of lava emplacement on a cooling planet. Instead, plains emplacement and later deformation are likely linked to the same mantle process, rooted in a global volcanic episode potentially triggered by large impacts comparable in scale to those that formed Caloris or Rembrandt basins.

22). Methods (Lines 235 to 402): I do not have any major comments or suggestions for this section and the extended data, only minor typos (see below). As mentioned in Section 6, this section should be referenced in the main text, particularly where the results rely on the methodology.

Thank you for this helpful comment. We have revised the manuscript to add clearer references to the Materials and Methods in the main text, particularly in sections where the interpretation depends strongly on the methodological framework.

23). Line 351: "we" should be "We"?

Thank you for catching this. We have corrected the capitalization.

24). Line 371: "we have constructed" should probably be "we construct" to keep it consistent with present tense throughout the method section.

Thank you for pointing this out. We have revised the tense for consistency in the Methods section.

25). Line 540: What is the "???" in the reference?

Thank you for catching this error. The citation has been corrected.

26). Line 612: This reference seems incomplete.

Thank you for pointing out this issue. We have re-checked the citations and ensured their completeness.

References:

Byrne, P. K., Klimczak, C., Celâl Şengör, A. M., Solomon, S. C., Watters, T. R., & Hauck, II, S. A. (2014). Mercury's global contraction much greater than earlier estimates. Nature Geoscience, 7(4), 301-307.

Crane, Kelsey T., and Christian Klimczak. "Tectonic patterns of shortening landforms in Mercury's northern smooth plains." Icarus 317 (2019): 66-80.

Di Achille, G., Popa, C., Massironi, M., Epifani, E. M., Zusi, M., Cremonese, G., & Palumbo, P. (2012). Mercury's radius change estimates revisited using MESSENGER data. Icarus, 221(1), 456-460.

Hall, G. P., et al. "The distribution of peak-ring basins on Mercury and their correlation with the high-Mg/Si terrane." Journal of Geophysical Research: Planets 126.9 (2021): e2021JE006839.

Man, B., Rothery, D. A., Balme, M. R., Conway, S. J., & Wright, J. (2023). Widespread small grabens consistent with recent tectonism on Mercury. Nature Geoscience, 16(10), 856-862.

Padovan, S., Tosi, N., Plesa, A. C., & Ruedas, T. (2017). Impact-induced changes in source depth and volume of magmatism on Mercury and their observational signatures. Nature communications, 8(1), 1945.

Peterson, G. A., Johnson, C. L., Byrne, P. K., & Phillips, R. J. (2019). Distribution of areal strain on Mercury: Insights into the interaction of volcanism and global contraction. Geophysical Research Letters, 46(2), 608-615.

Roberts, J. H., & Barnouin, O. S. (2012). The effect of the Caloris impact on the mantle dynamics and volcanism of Mercury. Journal of Geophysical Research: Planets, 117(E2).

Wang, Y., Xiao, Z., Chang, Y., Xu, R., & Cui, J. (2021). Short-term and global-wide effusive volcanism on Mercury around 3.7 Ga. Geophysical Research Letters, 48(20), e2021GL094503.

Watters, T. R., Robinson, M. S., & Cook, A. C. (1998). Topography of lobate scarps on Mercury: New constraints on the planet's contraction. Geology, 26(11), 991-994.

Watters, T. R., Daud, K., Banks, M. E., Selvans, M. M., Chapman, C. R., & Ernst, C. M. (2016). Recent tectonic activity on Mercury revealed by small thrust fault scarps. Nature Geoscience, 9(10), 743-747.

Response to Reviewer Comments:

Waned mantle upwelling contributes to a heavily deformed northern smooth plains on Mercury

Text in black: Reviewer comment/suggestion/question

Text in blue: Author response

Reviewer #1 (Dr. Stephan Loveless):

I have read the revised manuscript titled Wanted mantle upwelling contributes to a heavily deformed northern smooth plains on Mercury by Xie et al. In this manuscript, strain, gravity, and structural observations are used to determine that global contraction by itself, is insufficient to explain the unique structural anomalies that occur on the Northern Smooth Plains on Mercury. The manuscript describes a mantle upwelling model which demonstrates that the regional deformation of Mercury's NSP can be explained due to an underlying mantle upwelling.

I am pleased to share that the authors of this manuscript did a phenomenal job with their revisions. Their reasoning, and justification for their research is clearly stated, and the manuscript flows very well, allowing the reader to anticipate the research objectives of each subsequent section. The results of their work have a substantial impact on the Mercury tectonics community. For these reasons, I believe this manuscript should be accepted for publication following taking into account minor revisions (listed line-by-line below).

Thank you very much for your positive and encouraging evaluation of our revised manuscript. We are delighted that you found our revisions to be through, our reasoning clear, and our conclusions impactful for the Mercury tectonics community.

We greatly appreciate your time and expertise in providing constructive feedback. We have revised the manuscript in response to your comments. Below, we address each point in turn.

Line-by-line comments:

Introduction

1). Line 10: change "as the product" to "to be the product"

Thank you for your help suggestion. We have revised the manuscript accordingly.

2). Line 15: change "commonly used computational method extrapolates" to "commonly used computational methods extrapolate"

Thank you for your helpful suggestion. We have also revised the wording in response to Reviewer #2's comment (#1).

These words (line 14-20) now read:

There may be two possibilities: (1) the contractional strain of the NSP has been overestimated because the common method calculates strain using measurements (e.g., displacement-length ratios) derived from limited populations of representative structures [7,10-12], with the outcomes sensitive to the measurements and fault number. This concern is supported by a recent fault-number-independent analysis that yields lower estimates of Mercury's global contraction than earlier assessments [7,8].

3). Line 18: True. Loveless & Klimczak (2025) found lower values of radial contraction than Byrne et al. (2014) did using "optimal physical parameters", but these parameters were relatively conservative. I am not sure how strong of an argument can be made in this respect.

Thank you for your helpful comment, and we understand your concern. We view a key contribution of Loveless & Klimczak (2025) are their use of a fault-number-independent approach to assess Mercury's global contraction, yielding lower but self-consistent results relative to prior estimates. This outcome clearly indicates that earlier inferences were highly sensitive to the assumed fault number. This is the point we wish to emphasize: due to methodological limitations, contractional strain in the NSP may have been overestimated.

Stronger contractional strain in Mercury's Northern Smooth Plains

4). Line 51: I question a bit the choice to use the word "strong" with strain since strain is not necessarily a force, or a parameter that is related to force. Perhaps "greater contractional strain" would be more appropriate.

Thank you for your helpful suggestion, and we agree with it. We have revised this subsection's title accordingly.

5). Extended Data Figure 2 is a little confusing to read since the "other" contractional landforms are flipped on the "number of points access". Provided that this is a histogram and that a logarithmic scale is being used, a different display would be better. Maybe an overlay. Of the two histograms in the same direction.

Thank you for your helpful suggestion. We agree that histograms can facilitate comparisons between terranes. However, our goal here is to visualize the full distribution while also comparing strains between the NSP and other terranes. Volin plots provide a nonparametric view of the distribution and, with quartiles shown, allow direct comparison of numerical values. To improve readability, we revised the original figure by splitting the violins for each group with the same number of points.

The revised Extended Data Figure.2 (renamed to: Supplementary Fig.2) now reads:

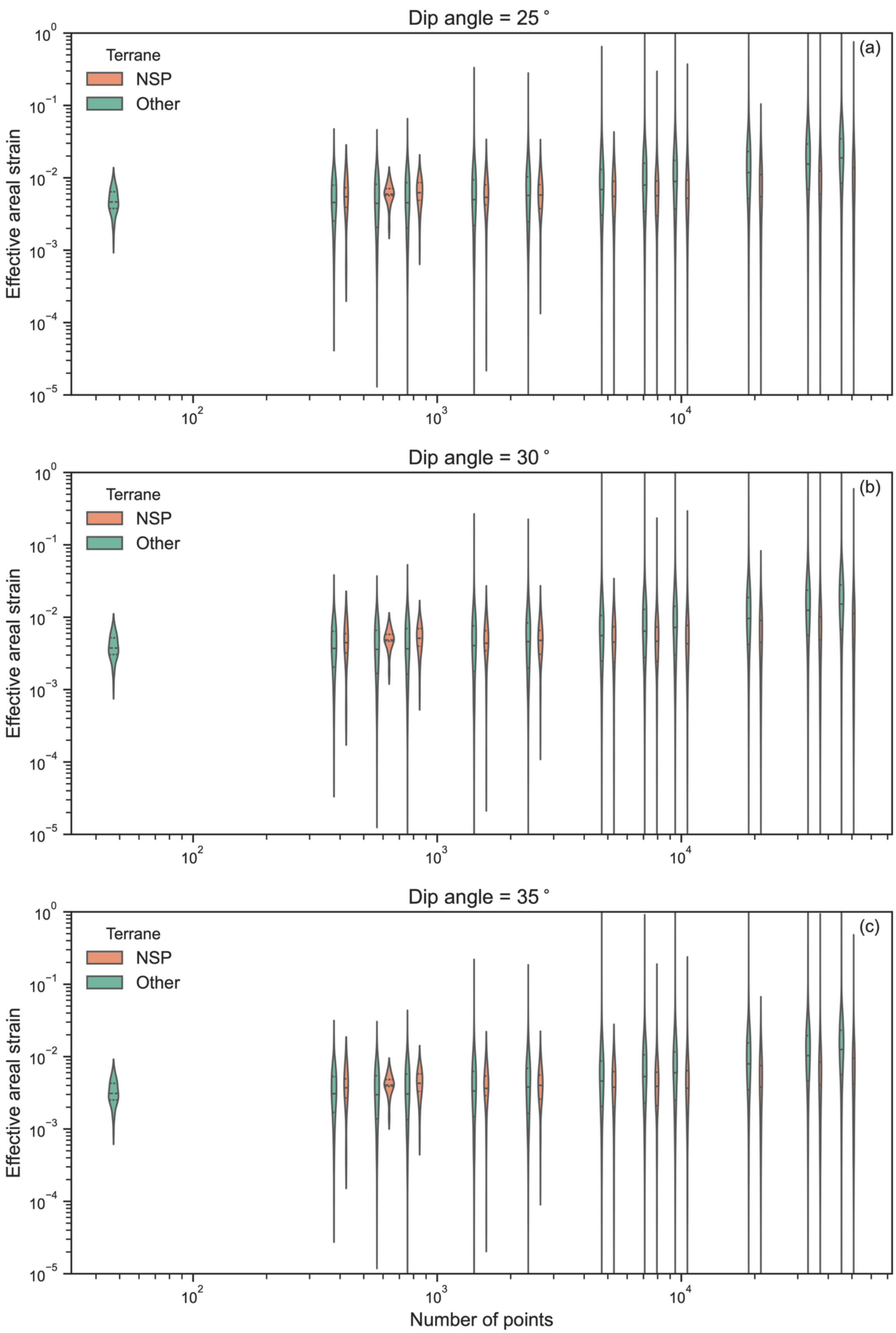
Dip angle = 25 °
(a)
Terrane
NSP
Other
Effective areal strain
Dip angle = 30 °
(b)
Dip angle = 35 °
(c)
Number of points

Supplementary Fig.2 The distribution of areal strains in logarithmic scale of the NSP and other terranes for various Fibonacci sampling points and dip angles.

6). Figure 1c) (and others) it is implied with how the figure is portrayed that the data set with 100 shortening landforms was the optimal data set for Loveless & Klimczak (2025), or Loveless & Klimczak (2025) produced the 100 shortening landform data set, which is incorrect. Loveless & Klimczak used optimal parameters on all 3 data sets used in the paper to provide "most-likely conservative estimates" for Mercury's radial contraction.

The work from Loveless & Klimczak (2025) did not have a data set of just 100 shortening landforms. Instead, it calculated global contraction using 3 different data sets each with different sample sizes of contractional landforms: 1) Byrne et al. (2014) ($n \approx 6000$), 2) Watters (2021) ($n \approx 600$), and Loveless et al., (2024, 2025) ($n = 100$).

Thank you for the clarification. In Fig.1c, we have removed the number indicating the faults used to calculate the results from Loveless & Klimczak (2025). Instead, the figure caption now provides additional details on the data used by Loveless & Klimczak (2025).

The revised Figure 1 now reads:

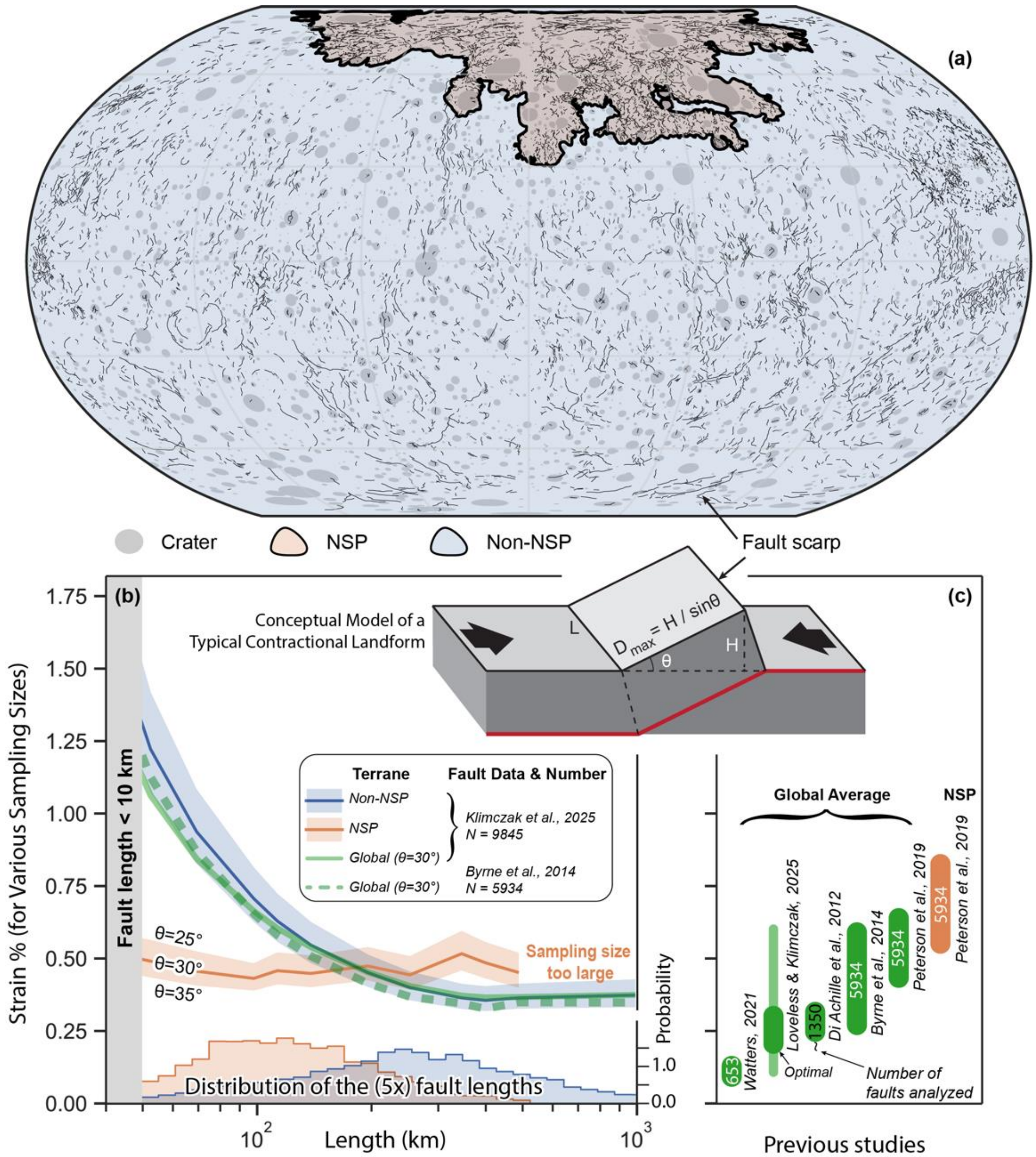

Fig1. (a) Global distribution of contractional landforms and craters across Mercury. (b) Contractional strain for various sampling sizes, which converges toward a stable value representative of the background contraction of each terrane. The distribution of 5x fault lengths suggests that the largest sampling windows adequately capture the mapped fault population. The inset depicts a conceptual model of a typical contractional landforms, where H, θ and L are the fault relief, dip and length, respectively, and D_{max} is the maximum displacement. (c) Comparison of Mercury's contractional strains from previous studies. Contractional structures are identified from a recently published global tectonic map of Mercury [22], and the crater database is after [5,23]. **Loveless and Klimczak [13] calculated global contraction using three different datasets [8,12,18] each with different sample sizes of contractional structures (n ≈6000, 600, 100, respectively**).

7). Line 96: I would hesitate in using the words that claim that the data set is "ours" as this work applied a methodology on a data set captured by Klimczak et al. (2025). Perhaps instead of "ours" use the term "the data set utilized here"

Thank you for you helpful suggestion. We have revised the manuscript accordingly.

8). Line 115: The difference in strain partitioning from lobate scarps and wrinkle ridges was not a result of Loveless & Klimczak (2025). In Loveless et al (2025) (the fault modeling paper) there was an overlap in the folded partitioned shortening strain, indicating that perhaps the difference between lobate scarps and wrinkle ridges were just the size of the shortening landform. It was also a result that lobate scarp shortening landforms accumulated more strain from folding than wrinkle ridges, but this did not account for how the strain was partitioned, it just was that lobate scarps had more strain likely because they were larger structures and were therefore faults that had matured longer.

Loveless et al. (2025) calculated the modeled strain from folding as well as modeled the heave from the fault, but how these were partitioned differently from lobate scarps and wrinkle ridges was not a result of this paper. There may be some suggestions of differences in fault dip, but the other statements of this sentence were not results of these papers. In particular, none of these statements are related to the results of the global contraction estimate paper form Loveless & Klimczak (2025).

Thank you for your thoughtful comments and clarification. We have revised the manuscript accordingly.

These words (line 115-124) now read:

Absolute shortening remains uncertain because key parameters (e.g., fault dip) and the partitioning between folding and fault slip at depth are not adequately constrained for individual landforms. Forward modeling indicates overlapping folding-accommodated shortening across lobate scarps and wrinkle ridges, and larger cumulative strain in lobate scarps likely reflects size or maturity rather than distinct partitioning (Loveless et al., 2025). Given these non-unique parameterizations, a fault-by-fault revision remains beyond our scope. We therefore retain a standard fault-based framework as a first-order estimator and focus on relative differences between terranes. Under these assumptions, the conclusion is robust: the NSP experienced stronger contractional deformation than the non-NSP terrane.

9). Lines 121-125: True, but if there were pre-existing faults underneath the smooth plains that continued to grow slowly (as suggested by the basement-activated thin-skinned tectonics of Loveless et al. (2025)) then contraction may still be present. Perhaps the work presented here works in conjunction with this model?

This is a good point. We agree that slow, continued slip on pre-existing basement thrusts beneath the smooth plains is plausible. Our strain mapping does not distinguish fault initiation from reactivation; therefore, basement-activated thin-skinned behavior is not mutually exclusive with our analysis. The key inference from our calculations is the NR-centered, anomalous high strain within the NSP, which is difficult to obtain from global contraction or basement reactivation alone. Our upwelling model supplies an additional driving force that explains both the strain

elevation and spatial pattern, which likely preferentially reactivates inherited faults beneath the NSP. We have addressed this point in line 233-234.

Controls on the upwelling-induced surface deformation

10). Figure 5b – perhaps this caption should be part of the methods section.

Thank you for your helpful suggestion. We consider it more appropriate to retain Fig.5b in the main text, as the pseudo-Rayleigh number (*pRa*) is introduced there and its potential role in investigating lithospheric properties (e.g., viscosity structure) is examined.

Tectonic evolution of the NSP and its implications to Mercury's interior

11). Paragraph between 342-347: conceptually, could it also be possible that there was early mantle upwelling that has since locked in place, causing a topographic high that of which stresses from global contraction coagulated on/around? Similar to the localization of stresses around buried crater rims?

This is an excellent point, and we agree it represents a plausible end-member for stress localization. In principle, a fossil upwelling could generate variations in topography that cause subsequent global contraction to preferentially localize, analogous to stress focusing around buried crater rims. A similar case comes from Watters et al. (2021), who explored the relationship between crustal thickness and contractional strain and proposed that mantle downwelling promoted crustal thickness and strain localization on Mercury.

Overall, we believe that these hypotheses will be better tested with high-resolution gravity and topography fields acquired by the BepiColombo mission in the near future.

Methods

Expression of contractional strain

12). Line 392: add the word "be" between the words "to" and "related". And add the word "to" between the words "related" and "D". The sentence should read: "…a scaling factor (γ) to be related to D and L…"

Thank you for your helpful suggestion. We have revised the manuscript accordingly.

13). Line 392: it is a little confusing if the elliptical fault geometries that is typically observed is applied to all of the faults in this analysis as it is discussed after small scale faults. Is this taken into account for all faults or just small structures? If just small structures, why?

Thank you for your thoughtful comment. In our study, the displacement-length scaling is applied to all mapped faults. We do not impose any specific per-fault displacement profile on individual faults. The reference to 'elliptical geometries' and a typical aspect ratio ($L/h_h \approx 2-3$) is used only in the small-scale case to eliminate the fault's down-dip height (h_h) in the Kostrov's

relation; it is not applied to large, through-going faults for which the down-dip height has saturated at approximately the brittle-ductile transition depth ($h_h \approx T$).

References:

Watters, T. R., James, P. B., & Selvans, M. M. (2021). Mercury's crustal thickness and contractional strain. Geophysical Research Letters, 48(17), e2021GL093528.

Reviewer #2 (Dr. Jupiter (Cheng) Hansen):

I want to thank the authors for making the effort in revising the manuscript. It was very good to see the improvement during the revision. The answers to my comments, questions and requests for small additions were certainly adequate. A minor justification is still essential with the new Fig. 2 (see comment for line 155).

Below are the line-by-line comments where I highlighted a few remaining typos or slightly awkward sentences. From my perspective, after adding the justification and fixing the few remaining typos or wordings, this is essentially good to go.

We sincerely thank you for your constructive feedback and for acknowledging the improvement in our revision. We are grateful that you found our previous answers adequate. We have revised the manuscript in response to your comments. Below, we address each point in turn.

Line-by-line comments:

1). Lines 15-16: Did you mean "extrapolates strain across the study area from"? "To" implies the strain is being projected into the region rather than calculated from structures within it.

We apologize for the confusion. We didn't mean that. The common strain (ϵ) calculation method (Eq.1) is expressed as (e.g., Byrne et al., 2014; Watters. 2021):

$$\epsilon = \frac{cos(\theta)}{A}\sum DL$$

Where θ is the fault dip, D is the (maximum) displacement, and L is the length. A is the study area. The displacement can be estimated by $D = H/sin(\theta)$, with H the relief.

The displacement and length are related by (Eq.2):

$$D = \gamma L$$

Where γ is the displacement-length ratio. Consequently, Eq.1 can be reformulated as (Eq.3):

$$\epsilon = \frac{\gamma cos(\theta)}{A}\sum L^2$$

Therefore, we only need to measure the fault length (L) and relief (H) and calculate the displacement-length ratio (γ) to estimate strain. Theoretically, every structure should be measured. However, because the number of structures can reach the hundreds or thousands or even more, in practice, a representative population of structures (e.g., some large ones) is selected to measure their lengths and relief, and the displacement-length ratio is obtained by fitting (e.g., Watters. 2021). Finally, it is assumed that the remaining structures share the same displacement-length ratio. In this way, only the lengths of those structures are needed (which are easy to measure) to estimate the overall strain in the study area.

This method only extrapolates the displacement-length ratio derived from a limited set of structures to the rest, while the overall strain is still calculated from all structures. The outcomes are therefore sensitive to the measurements and fault numbers.

For clarity, these words (line 14-20) now read:

There may be two possibilities: (1) the contractional strain of the NSP has been overestimated because the common method calculates strain using measurements (e.g., displacement-length ratios) derived from limited populations of representative structures [7,10-12], with the outcomes sensitive to the measurements and fault number. This concern is supported by a recent fault-number-independent analysis that yields lower estimates of Mercury's global contraction than earlier assessments [7,8].

2). Lines 95-96: "Although a difference of nearly 4,000 mapped faults between our dataset and that of Byrne et al…." reads awkwardly. The sentence starts with "Although" but lacks a proper contrast clause. Probably rewrite it as “"despite a difference of nearly 4,000 mapped faults..."

Thank you for your helpful suggestion. We have revised the manuscript accordingly.

3). Line 155: Instead of "perpendicular to the radial", may be just directly say concentric? Unless I totally misunderstood the meaning of the strike direction. Also, it seems that the far-field faults match the elastic model prediction, but the proximal ones do not. Why is that? This is worth a sentence of clarification.

In Fig.2c&d, we visualize the dominant thrust-fault strikes and their angular differences from the radial trend centered on the NR. According to Anderson's fault theory, thrust-fault strikes are perpendicular to the maximum principle stress orientation, we can therefore infer the maximum principle stress orientation given the fault strike. For example, if the thrust-fault strike is parallel to the radial trend, implying a concentric compressive stress field around the NR. Accordingly, we use 'concentric' to describe the inferred stress field, whereas it would be inaccurate to apply 'concentric' to the fault strikes themselves. We have addressed this point in line 163-165.

Our result (Fig.2) shows that the dominant strikes of the far-field fields are mainly parallel to the radial trend, while those in the proximal region are mainly perpendicular to the radial trend. This indicates that the stress field is concentric around the NR, whereas the maximum principle stress orientation in the proximal region should be parallel (or 'vertical' in a 3D perspective) to the radial trend. This pattern is consistent with our 3D model prediction (line 265-274; Extended Data Fig.9 (renamed as: Supplementary Fig.9)).

4). Fig 2 caption: "anomaly versus Fault orientation pattern" The "fault" should not be capitalized.

Thank you for pointing out this error. We have revised the caption accordingly.

5). Line 343: The phasing “later in its history than is often assumed because its relatively thin thickness” is awkward and incomplete. Should probably be something like "because of its relatively thin (crustal?) thickness"

We intended to imply that Mercury’s mantle may have remained regionally active into its late evolutionary stage. This implication departs from our prior understanding. However, as noted in the main text, this inference does not require vigorous global or large-scale convection at late times.

In general, because Mercury’s mantle (or more broadly, its silicate shell) is thin, approximately 400 km, progressive cooling makes sustained or large-scale convective activity unlikely in the late evolutionary stage. This view is supported by thermodynamic studies of Mercury (Hauck et al., 2013; Margot et al., 2018; Knibbe & van Westrenen, 2018; Xie et al., 2022). The longevity of mantle convection itself is more likely controlled by mantle and deeper inside the planet rather than the crust.

For clarity, these words (line 346-348) now reads:

More broadly, the results imply that Mercury’s mantle remains regionally dynamic later in its history. This inference challenges our prior understanding of an inactive mantle because of its relatively thin thickness [37,40-42].

6). Line 366: “reorganized the regional stresses field” should be “reorganized the regional stress field”

We agree with you and have revised the manuscript accordingly.

7). Line 451: “are then subjected statistical analysis” should be “"are then subjected to statistical analysis"

Thank you for catching this. We have revised the manuscript accordingly.

8). Line 466: “In this cases” should be “In this case” as singular

Thank you for pointing out this error. We have revised the manuscript accordingly.

9). Line 496: “in this cases” should be “in this case”

Thank you for pointing out this error. We have revised the manuscript accordingly.

References:

Byrne, P. K., Klimczak, C., Celâl Şengör, A. M., Solomon, S. C., Watters, T. R., & Hauck, II, S. A. (2014). Mercury’s global contraction much greater than earlier estimates. Nature Geoscience, 7(4), 301-307.

Hauck, S. A., Margot, J. L., Solomon, S. C., Phillips, R. J., Johnson, C. L., Lemoine, F. G., ... & Zuber, M. T. (2013). The curious case of Mercury's internal structure. Journal of Geophysical Research: Planets, 118(6), 1204-1220.

Margot, J. L., Hauck II, S. A., Mazarico, E., Padovan, S., & Peale, S. J. (2018). Mercury's internal structure. arXiv preprint arXiv:1806.02024.

Knibbe, J. S., & van Westrenen, W. (2018). The thermal evolution of Mercury's Fe–Si core. Earth and Planetary Science Letters, 482, 147-159.

Watters, T. R. (2021). A case for limited global contraction of Mercury. Communications Earth & Environment, 2(1), 9.

Xie, J. C., Zhang, M., & Huang, C. L. (2022). Influence of megaregolith on the thermal evolution of Mercury’s silicate shell. Research in Astronomy and Astrophysics, 22(3), 035026.

I have read the manuscript titled *Waned mantle upwelling contributes to a heavily deformed northern smooth plains on Mercury* by Xie et al. In this manuscript, the areal strain from thrust fault-related landforms was found to be unique in the Northern Smooth Plains, particularly around the Northern Rise. Mantle upwelling was then modeled as a way to explain the existence of the Northern Rise, and as a way to account for the strain anomaly in this area. I had found the results of this manuscript very interesting and relevant and thus warrants consideration for publication. However, there were many aspects of this manuscript, especially in the methodology, or justification of methodology, that had substantial holes that required further explanation. For this reason, I believe that the manuscript should be potentially **reconsidered for publication following major revisions.**

I have provided an in-depth review below, starting with general comments, followed by line-by-line comments. I have tried to be as constructive and helpful as possible.

Best,

Dr. Stephan Loveless

General Comments

It is hard to tell what exactly the methodology is. In a Nature formatted paper the detailed methods come at the end, but nearly no information is provided as to what is done so that the reader distinctly understands the goal of the paper. Aspects like, *preferred models* (figures 4 and 5), or the *reliable strain estimates* referenced in the section titled *Stronger Contractional Strain in Mercury's Northern Smooth Plains*, are not defined, sometimes even in the **methods** section, leaving the reader to wonder what is truly being used to constrain the models, a crucial point of the paper. I suggest that the organization of the manuscript be reviewed as at this stage it feels that the main text relies on the reader jumping to the methods at the end of the paper too much.

More generally, the results presented in the section **Stronger Contractional Strain in Mercury's Northern Smooth Plains**, are simply just stated, without any succinct, general comments about what was actually done in the study. The reader should have a general idea of the methodology without getting into the details presented in the methods section.

Stress and strain terms are used intermixed throughout the paper. **Extension** and **contraction** are **strain** terms. **Tension** and **Compression** are **stress** terms. The authors calculate both stress and strain throughout the paper, but it is unclear what is being referenced to and where. Furthermore, the signs for stress and strain must be defined as they can be different depending on the methodology. For example, is compression positive or negative? Is contraction positive or negative? How does this play a role in the calculation of the strain anomaly in the Northern rise?

Additionally, lower dip angle does not *physically* mean that a fault experiences more contraction, an argument this paper makes with respect to shortening landforms in the northern smooth plains. This is an artifact of the previous method used to estimate Mercury's global contraction when just comparing a simple-shear fault geometry to the relief of a shortening landform. The correlation between the two are an artifact of this equation. Loveless & Klimczak (2025) in AGU Advances had found greater estimates for radial contraction with steeper dip angles.

The relationship between fault dip and displacement, is sort of a chicken-before-the-egg argument. Provided one trigonometric relationship, it has been widely accepted that low dip = greater displacement and therefore, greater strain. However, just because a fault has a low dip, does not guarantee that it has larger displacement. Displacement is more so correlated and physically aligned with the size of the fault (i.e., length). A fault will mature, grow in length, and continue to experience displacement. Other factors will influence the dip of the fault but does not necessarily guarantee that a fault with low dip means you have a large amount of displacement.

On a last note, this paper is focused on the northern smooth plains but does not include its formal name throughout the entire manuscript: the Borealis Planitia.

The rest of the comments are line by line notes:

Introduction

Line 1: get rid of the word "as". Should read as "… offers a unique window…"

paragraph one is missing some key information. Mainly the second sentence is quite broken up when it doesn't need to be & terms like "relatively" young and "tectonic" structures are not provided good examples.

Line 9: this may be a bit pedantic, but grammatically, it would read better as "thrust fault-related, scarps" as opposed to "thrust-fault scarps".

Line 18: it is more appropriate to use the term "contractional" than "compressive" as deformation is what is being described (i.e., strain). Compression is a *stress* not a *strain* term. Same with line 19, line 55, many of the figures. Extensional features (i.e., normal faulting) form in compressive stress regimes, it is only the configuration of principal stresses that differs between contractional (thrust faulting) and extensional (normal faulting) strain domains. (this is referred to correctly in line 32)

Line 21: I would suggest removing "on the other hand" as it is later stated "if the latter stands" making referencing the alternate possibility redundant. Furthermore, the grammar of this

sentence is incorrect. Perhaps reword to “If the latter is true, one must then ask, ‘what might these mechanisms be?’.

Line 22 to 27: this is an extremely long, run-on sentence. This should be separated into 3 or 4 different sentences.

Line 27: it is written that “Most importantly, the volcanic loading is unlikely to support sustained geological activity” but is not suggested why that may be. I am assuming that there are two primary reasons why this is being suggested: first, lithospheric flexure from loading on Earth predominately forms extensional features, so we would not expect contraction. Second, Mercury is cold, its mantle is likely extremely rigid at this state, so subsidence would only be possibly in its ancient history, making it difficult to argue that recent lithospheric subsidence is likely to have occurred.

Stronger Contractional Strain in Mercury’s Northern Smooth Plains

Line 43: the method descried by Byrne et al. (2014); Watters et al. (1998) and Watters (2021) is *not* a kinematic model. These approaches referenced here estimate global contraction based on the inferred cumulative strain of a population of observed thrust faults. An important note is that the relationship in all of these studies between dip angle and the amount of estimated global contraction is *an artifact of the method* in relating the estimated displacement to the vertical relief. Physically, lower dip angles do not mean a greater amount of global contraction. Loveless & Klimczak (2025), a study that resolved the discrepancy of the fault population studies mentioned in this line, showed that greater dips actually predict larger amounts of contraction.

Lines 50-51: how does a minimum length threshold “avoid the incompleteness” since applying a threshold is essentially constraining the data. This sentence needs to be revisited for clarity.

Line 59: “our results indicate”, What has been done? At this stage, the distinct methods that were used aren’t being referenced, only what data sets were accessed, making it a bit confusing.

Line 69: “when the sampling size is sufficiently large… …the median strain values reliably represent overall contraction…” the term *reliably* is used here, but there is no metric stated as to what strain estimates are *reliable* or not.

Lines 72 to 78: the results of the Loveless et al. (2025) study are 1) of lobate scarp and wrinkle ridge *endmember* structures, 2) indeed, it was found that lobate scarp endmember structures had greater dips than that of wrinkle ridge endmembers, but this is just a correlation. Using simple trigonometry, and just relating the relief and the fault dip to

estimate the displacement, one would find that greater fault dips requires less displacement. However, this is one relationship. In the study referenced, displacement and folding along the fault plane were partitioned differently. Additionally, on average, shortening landforms in the intercrater plains were found to have 1) greater amounts of displacement (i.e., almost double), and 2) greater amount strain partitioned to folding than those in the NSP. These arguments of this paper directly counter the arguments made here. Especially since, generally, larger shortening landforms were found to have faults that had greater dip.

Why the distribution of fault lengths provides a comprehensive sample of the fault population is not discussed.

Almost nothing provided regarding the methodology to the point where the paper is hard to follow at this stage. A very general, short outline needs to be provided for some context.

Spatial Distribution of Deformation in the NSP

Line 95: "most regions of the NSP exhibit [**a**] negative strain anomaly". If it's contraction, shouldn't this strain be negative to begin with? What is the sign convention here? Meaning a negative anomaly would be more contraction, unless all of the contractional strain values have been converted to be positive which needs to be stated.

Numerical Modeling of Mantle Upwelling Beneath The NSP

Lines 155-156: "Because mantle upwelling likely generated melt that formed the NSP lavas, the contractional deformation must have occurred after lava emplacement". Not all contractional deformation occurred after emplacement. Some faulting may have occurred prior and then reactivated into the smooth plains units. Especially since Loveless et al. (2025) found faults in the smooth plains to form with the bedrock

Figure 4: Compressional should be changed to contractional. What are the preferred models? How are they defined? This is not stated.

Controls on Surface Deformation

Lines 166-173: doesn't this method assume that the lithosphere is behaving viscously? And isn't the goal of this paper to relate mantle upwelling to the *brittle* deformation of the lithosphere? These assumptions should be stated (*unless they are stated later in the methods*), unless it is assumed that this upwelling occurred so early that much of the lithosphere is behaving in a viscous or ductile manner. Either way, these need to be stated.

Lines 178 to 180: this should all be one sentence: *"Using the inversion-derived areal density of the mass sheet together with the surface strain estimates, we apply pRa to constrain Mercury's lithospheric properties."*

Figure 5: strain is *contractional*. Compression is a *stress* term. Furthermore, the equation should be introduced in the main text of the paper not here.

Tectonic Evolution of the NSP and its Implications to Mercury's interior

Line 193: deformation is already plural in this sense. Remove the "s" from the end of the word.

Lines 189-198: this is an interesting finding of the paper. If extension preceded contraction, then much of these thrusts in the NSP can be re-activated normal faults (inverted into thrusts). A topic that should be touched on.

Lines 199-203: I am hesitant to agree with persistent mantle convection. There is a rheological assumption of the mantle convection model shown here, assuming that the mantle behaves viscously. However, if Mercury is sufficiently cold, which it is as it has undergone global contraction, then its mantle would be too rigid for further convection. However, a remnant upwelling bulge perhaps could provide something structural which stresses can coalesce on (similar to the formation of ghost craters) and therefore, enhance the localized brittle deformation due to ongoing global contraction (just a thought, and I now see that mantle convection is unlikely is stated in line 204).

Figure 6: change compression in the right side of the figure into contraction. Also, in panel b), wrinkle ridges are shown to be only penetrating the smooth plains, however, they likely host faults that penetrate deeper than the smooth plains emplacement.

Line 225-226: upwelling is likely regional influencing *alongside* global contraction; however, I fail to see how this study shows that global contraction is being overridden. Yes, there may be localized deformation due to upwelling, but there is still contraction in the northern rise, implying that there may be a combination of events, but not necessarily one overriding the other. Global contraction is still likely the dominant stress source.

Methods

Expression of contractional Strain

Line 243: the term "strain" should be used instead of "stress". And if it is "known to accommodate" a reference is needed here.

Line 248: why are "geochemical" properties being used as a reference here? This should not have anything to do with "geochemical" properties.

Line 258-262: there are many studies that look into the lithospheric thickness of Mercury (not just the one referenced here). Furthermore, Loveless et al. (2025) found depths of faulting well below 30 km (some up to 40-45 km).

Line 265-266: “accordingly, we propose a quantitative classification scheme for contractional landforms: fault’s length extending to or beyond this depth are categorized…” what exactly is being compared here? The length is the observed, map-view length of the fault (or surface break), while the depth of the fault is how deep the fault is penetrated. Why is it that the length is being compared to the depth of faulting? How is the “fault’s length extending to or beyond this depth”?

Extraction of thrust fault’s relief

Lines 289-291: what does “the minimum value between the local fault length (*L*) and 50 km” mean? It is hard to picture what exactly is being described here. Shouldn’t the transections be roughly perpendicular to the length of the fault? how is the local fault length defined?

Calculation of areal strain

Lines 318 – 322: is there an appropriate reference regarding why faults should be weighted towards the strain? Is this really representative of areal strain?

Lines 323-326: what is the defining point between a small and large structure? This is an important definition.

Line 351: the “w” in we need to be capitalized.

Extended Data figure 2: there appears to be no difference between the three panels.

Extended Data figure 4: if this is stress, then the term extensional should be changed to Tensile
